# Association of early chloride-bicarbonate joint phenotypes with in-hospital mortality among intensive care unit patients with sepsis: a dual-database study

**DOI:** 10.3389/fmed.2026.1947296

**Published:** 2026-09-16

**Authors:** Liang Wang, Zhihua Xu

**Affiliations:** 1 School of Medicine, University of Electronic Science and Technology of China, Chengdu, China; 2 Mianyang Central Hospital, Mianyang, China

**Keywords:** acid-base balance, bicarbonate, chloride, eICU, intensive care unit, MIMIC-IV, mortality, sepsis

## Abstract

**Background:**

Associations of chloride and bicarbonate abnormalities with sepsis outcomes have been studied separately, but whether a prespecified paired classification provides reproducible and interpretable risk characterization remains uncertain.

**Methods:**

We included adults with sepsis in MIMIC-IV v3.1 and eICU v2.0. Chloride (<98, 98–107, >107 mmol/L) and bicarbonate (<22, 22–26, >26 mmol/L) defined a 3 × 3 phenotype. In-hospital mortality was analyzed using multivariable logistic regression and predictive mean matching multiple imputation. Robustness was assessed through analyses of temporal ordering, threshold selection, cross-database heterogeneity, external transportability, and first-day changes in chloride and bicarbonate.

**Results:**

The cohorts included 25,842 MIMIC-IV stays and 37,045 eICU stays, with mortality rates of 16.31% and 15.50%, respectively. In complete-case Model 3b, the phenotype was globally associated with mortality in both cohorts; the low-low contrast was individually significant in eICU but not MIMIC-IV. The low-low association was supported in multiple-imputation analyses (MIMIC-IV OR 1.276, 95% CI 1.066–1.527; eICU OR 1.507, 95% CI 1.277–1.779) and in strict pre-exposure analyses (OR 1.700 and 1.787, respectively). Formal 4-df categorical interaction tests were not significant (*P* = 0.095 and *P* = 0.109). MIMIC-IV-derived data-driven cut points did not significantly outperform the clinical 3 × 3 phenotype in eICU (CART vs. clinical ΔAUROC 0.0009, 95% CI −0.0010 to 0.0029). In the main MIMIC-IV-to-eICU validation, adding the clinical phenotype left discrimination essentially unchanged (ΔAUROC 0.0010, 95% CI −0.0013 to 0.0031), although the Brier score improved modestly (ΔBrier −0.0011, 95% CI −0.0016 to −0.0006); NRI and IDI did not support improved discrimination or reclassification. Incident and persistent low-low states were both associated with higher subsequent mortality in 24 h landmark analyses in both cohorts.

**Conclusions:**

Prespecified chloride-bicarbonate phenotypes were associated with mortality in both databases, and persistence or incident development of the low-low state during the first ICU day was associated with higher subsequent mortality. The 3 × 3 framework should therefore be interpreted primarily as a transparent, direction-preserving approach to descriptive risk characterization within a broader clinical assessment, rather than as a stand-alone prediction instrument, therapeutic threshold, or treatment guide.

## 1 Introduction

Sepsis is a life-threatening organ dysfunction caused by a dysregulated host response to infection (1). Clinical deterioration can be rapid, making routinely available laboratory measurements attractive for early bedside assessment when they can be interpreted within a physiologically meaningful framework.

Electrolyte and acid-base abnormalities are common in sepsis. Bicarbonate reflects metabolic acid-base status, whereas chloride contributes to extracellular strong-ion balance, renal electrolyte handling, and the acid-base effects of intravenous fluids (2, 3). Hyperchloremia, hypochloremia, nonlinear chloride-mortality relationships, and longitudinal chloride trajectories have been associated with adverse outcomes in septic or critically ill populations (4–8). Bicarbonate also shows a nonlinear relationship with mortality in sepsis, with increased risk at both low and high concentrations (9). However, most studies have evaluated chloride and bicarbonate separately.

Mixed acid-base states may be obscured when chloride and bicarbonate are interpreted separately. Low bicarbonate may occur with lactic acidosis, renal failure, gastrointestinal bicarbonate loss, or as part of a compensatory process, whereas chloride is influenced by renal or gastrointestinal losses, diuretic use, and chloride-containing fluids (3, 10, 11). Joint interpretation of the two measurements is further motivated by the different prognostic implications of distinct types of metabolic acidosis (12).

Data-driven phenotyping has also identified reproducible clinical subtypes in sepsis (13), and electrolyte-based phenotyping is feasible. Xiao et al. derived three critical-illness phenotypes using K-means clustering of distributional features from seven electrolytes in MIMIC-IV and validated them in eICU and AmsterdamUMCdb (14). Such methods can capture higher-dimensional latent patterns but depend on variable selection, standardization, and model derivation. Our 3 × 3 framework instead uses two physiologically linked measurements and fixed clinical cut points, intentionally trading statistical flexibility for transparent directionality and assignment without model retraining.

The remaining question, therefore, is not whether chloride or bicarbonate is a novel biomarker, but whether their paired, direction-preserving classification is reproducibly associated with mortality and retains useful information relative to continuous encodings and data-driven thresholds. A further clinically relevant question is whether persistence or development of a high-risk joint state during the first ICU day is associated with subsequent mortality.

We therefore examined a prespecified 3 × 3 chloride-bicarbonate phenotype in MIMIC-IV v3.1 and eICU v2.0, using normal chloride plus normal bicarbonate as the reference. Additional analyses evaluated CHBI, separate continuous chloride and bicarbonate terms and their interaction, multiple imputation, strict pre-exposure adjustment, threshold and spline sensitivity, harmonized cross-cohort heterogeneity, MIMIC-IV-to-eICU transportability, and longitudinal changes during the first ICU day.

## 2 Materials and methods

### 2.1 Data sources and ethical approval

This retrospective study used MIMIC-IV v3.1 and the eICU Collaborative Research Database v2.0 (15–18). Both are de-identified critical-care databases available through PhysioNet under institutional approvals and credentialed-access data-use agreements. Data extraction was performed by authors who had completed the required human-subjects research training and were operating under the applicable data-use agreements. Because only de-identified retrospective data were used, no additional individual informed consent was required.

The study investigated whether early paired chloride and bicarbonate values could define interpretable phenotypes associated with mortality in ICU patients with sepsis. Reporting followed the RECORD guidance for studies using routinely collected health data (19).

MIMIC-IV v3.1 contains single-center electronic health record data from Beth Israel Deaconess Medical Center in Boston, United States (2008–2022), whereas eICU v2.0 contains multicenter data from 335 units at 208 United States hospitals (2014–2015) (15–18). The databases were analyzed independently, and reproducible cohort-construction and validation scripts were developed for each database version.

### 2.2 Data extraction and quality control

Chloride and bicarbonate/total CO_2_ measurements obtained from 0 to 24 h after ICU admission were extracted from source laboratory tables using version-specific item identifiers. Measurement times were converted to minutes from ICU admission, and candidate chloride-bicarbonate pairs were required to be within 360 min of each other. We applied database-specific deterministic selection rules. In MIMIC-IV, exact-time pairs were prioritized, followed by the earliest eligible pair; in eICU, the earliest eligible pair was prioritized, followed by the smallest absolute time interval, with deterministic tie-breakers for measurement times. All selected pairs in the final MIMIC-IV paired cohort were exact-time pairs, and nearly all selected eICU pairs were exact-time pairs. The reproducibility package provides the complete item identifiers, tie-breaking rules, and extraction checks.

Exposure-level plausibility handling followed database-specific source-construction procedures. In MIMIC-IV, exact-time paired exposures outside 50–160 mmol/L for chloride or 2–60 mmol/L for bicarbonate were excluded before phenotype and CHBI derivation. In eICU, the primary paired exposure retained numeric source measurements; prespecified bounds of 70–140 mmol/L for chloride and 5–45 mmol/L for bicarbonate were used to flag implausible values in source QC and sensitivity outputs. Separate downstream descriptive/QC processing recoded values outside 70–140 mmol/L for chloride and 5–45 mmol/L for bicarbonate as missing for continuous source-variable summaries; this recoding did not alter the already-derived phenotype or CHBI and was not the analytic-sample definition for the shared-sample Model 2b representation comparisons. These rules and affected counts are documented in the reproducibility outputs.

Most clinical covariates were derived from a peri-admission window of −6 to +24 h, although timing and aggregation differed between databases. Laboratory covariates generally used the first available value. Severity scores and treatment indicators were defined according to database-specific conventions. Selected ventilation, renal replacement, and AKI variables were available only at the stay level. Model 3b was therefore regarded as a peri-admission explanatory association model rather than a strict pre-exposure prediction model. A separate strict pre-exposure analysis assessed temporal ordering.

### 2.3 Study population

Adults with sepsis were identified using database-specific derived cohorts that operationalized the Sepsis-3 criteria (1). Database-specific rules were applied to select ICU stays, and a valid paired chloride-bicarbonate exposure was required. Because a patient may contribute more than one eligible eICU stay, the primary eICU analysis included all eligible stays, with a sensitivity analysis restricted to the first eligible ICU stay per patient. The reproducibility package documents cohort construction, duplicate checks, deterministic selection rules, source-object provenance, and row-count validation.

The primary analysis included ICU stays with available paired chloride and bicarbonate values and known in-hospital mortality status. ICU stays without outcome data were excluded from the in-hospital mortality analysis. No *a priori* sample-size calculation was performed; study size was determined by all eligible ICU stays meeting these criteria in the specified database versions.

### 2.4 Exposure definitions

The primary exposure was the 3 × 3 chloride-bicarbonate joint phenotype. Chloride was classified as low (<98 mmol/L), normal (98–107 mmol/L), or high (>107 mmol/L), and bicarbonate as low (<22 mmol/L), normal (22–26 mmol/L), or high (>26 mmol/L). These categories yielded nine joint phenotypes. Normal chloride plus normal bicarbonate served as the reference phenotype.

As complementary quantitative exposures, CHBI was defined as MIMIC-IV-standardized chloride minus MIMIC-IV-standardized bicarbonate; the MIMIC-IV component means and SDs were also applied in eICU to preserve a common exposure scale. For within-cohort association analyses, CHBI and absolute CHBI were expressed per cohort-specific 1-SD increment. MIMIC-IV-derived CHBI quartile cut points and training-set standardization were used in transport analyses. Chloride, bicarbonate, CHBI, CHBI quartile, and joint-phenotype exposure variables were not imputed.

### 2.5 Outcome definitions

The primary outcome was in-hospital mortality. We also performed database-specific secondary analyses at 28 and 90 days. For MIMIC-IV, recorded death dates were evaluated relative to hospital admission. eICU captures only deaths occurring during the index hospitalization; therefore, its day-specific endpoints were not interpreted as conventional postdischarge all-cause mortality.

### 2.6 Statistical analysis

Cohort characteristics were summarized separately for MIMIC-IV and eICU. Continuous variables are presented as medians [interquartile ranges], and categorical variables as counts (percentages). Characteristics were also summarized across the nine chloride-bicarbonate joint-phenotype groups. Because the primary exposure comprised nine categories, covariate imbalance was evaluated using the maximum pairwise standardized mean difference across phenotype groups. Missingness in key clinical and laboratory variables was reported for each cohort.

Associations between chloride-bicarbonate exposures and mortality were assessed using multivariable logistic regression, with odds ratios (ORs) and 95% confidence intervals (CIs). Model 3b was the prespecified primary multivariable adjustment model. Covariate timing and aggregation were defined as described above. In MIMIC-IV, Model 3b adjusted for age, sex, race, ICU care unit, admission type, Charlson comorbidity index, dynamic SOFA score, dynamic AKI stage, vital-sign extremes, creatinine, blood urea nitrogen, lactate, invasive mechanical ventilation, renal replacement therapy, sodium bicarbonate therapy, and vasoactive therapy. In eICU, Model 3b adjusted for age, sex, race, geographic region, ICU unit type, Charlson comorbidity index, APACHE IV score, SOFA score, OASIS score, creatinine, blood urea nitrogen, lactate, invasive mechanical ventilation, sodium bicarbonate therapy, and vasoactive therapy. Differences in variable availability, scoring systems, and timing definitions required cohort-specific adjustment sets. Model 3b should therefore be regarded as a peri-admission explanatory association model rather than a model restricted to information available before measurement of the paired exposure.

Within the same modeling framework, we evaluated CHBI per 1-SD increment, absolute CHBI per 1-SD increment, CHBI quartiles, and the 3 × 3 chloride-bicarbonate joint phenotype. For categorical exposures with more than two levels, likelihood-ratio or global Wald tests were used, as appropriate, to assess overall associations. The 3 × 3 joint phenotype was the primary exposure of interest because it preserves the direction and clinical interpretability of both chloride and bicarbonate abnormalities.

For direct representation comparisons, cohort-specific Model 2b covariates (specified in the Supplementary analytical methods) were extended with standardized chloride, standardized bicarbonate, both continuous main effects, their product interaction, CHBI encodings, or the 3 × 3 phenotype in a shared analytic sample. Model comparisons used nested likelihood-ratio tests and 500 paired fixed-prediction bootstrap resamples. Apparent AUROC, Brier score, log loss, AIC, and BIC were evaluated as development-sample measures rather than internally validated estimates with optimism correction. In separate complete-case Model 3b samples, 2-df nested tests assessed incremental categorical main-effect information and a 4-df likelihood-ratio test assessed interaction beyond additive chloride and bicarbonate categories.

To assess robustness to additional acid-base adjustment, we fitted three prespecified extended sensitivity models. Model 4b-Full additionally adjusted for pH, arterial partial pressure of carbon dioxide (PaCO_2_), anion gap, and albumin. Model 4b-NoAG additionally adjusted for pH, PaCO_2_, and albumin, excluding anion gap. Model 4b-pH/PaCO_2_ added pH and PaCO_2_ to Model 3b. These models were treated as sensitivity analyses rather than primary analyses because acid-base variables may lie on the same physiologic pathway as chloride and bicarbonate, anion gap is mathematically coupled with chloride and bicarbonate, and several of these variables had substantial missingness.

A formal multiple-imputation sensitivity analysis was performed using donor-based predictive mean matching because several covariates had missing values. Twenty imputed datasets were created using chained equations with five iterations and five nearest donors. Only continuous standardized covariates required for Model 3b and Model 4b-NoAG were imputed. The outcome, chloride, bicarbonate, CHBI, CHBI quartile, and chloride-bicarbonate phenotype were not imputed. Logistic regression models were fitted in each of the 20 imputed datasets, estimates were combined using Rubin's rules (20), and convergence of the fitted regression models was checked in every imputed dataset.

Primary phenotype ORs were synthesized across cohorts using inverse-variance fixed-effect meta-analysis. Cochran's Q and *I*^2^ were reported as descriptive measures of cross-cohort heterogeneity and interpreted cautiously because only two cohorts were available. In addition to the original pooled exposure-by-cohort interaction analysis using database-specific adjustment blocks, we fitted a harmonized sensitivity model with the same covariate set in both databases: age, sex, harmonized race, Charlson index, SOFA, creatinine, blood urea nitrogen, lactate, septic shock, AKI, invasive ventilation, sodium bicarbonate therapy, and vasoactive therapy. Cell-specific cohort differences were tested on the log-OR scale, with *P* values adjusted using the false-discovery-rate procedure.

We assessed transportability by training a prespecified common-variable model in MIMIC-IV and applying its coefficients and MIMIC-IV-derived standardization parameters to eICU without refitting; the portable predictor set is specified in the Supplementary analytical methods. Transport analyses were reported according to TRIPOD principles (21). The base model and extensions incorporating the phenotype or alternative chloride-bicarbonate encodings were evaluated using AUROC, Brier score, log loss, calibration intercept offset (calibration-in-the-large), calibration slope (22), and decision-curve analysis (23). Calibration-in-the-large was estimated from a logistic recalibration model with the logit of predicted probability entered as an offset and the slope fixed at 1. Calibration slope was estimated from a recalibration model in which the intercept and coefficient of the predicted logit were jointly estimated. Differences were estimated using 500 paired eICU case-resampling bootstrap replicates while predictions were held fixed. For the prespecified base-vs.-phenotype comparison, we also calculated integrated discrimination improvement (IDI) and category-free net reclassification improvement (NRI), with paired bootstrap confidence intervals. Relevant updated TRIPOD + AI reporting principles were also considered (24).

Sensitivity analyses included variation in pairing intervals and exposure windows, restriction of eICU to the first ICU stay per patient, evaluation of database-specific secondary outcomes, and prespecified subgroup analyses. Subgroup interaction analyses were exploratory.

We assessed sensitivity to threshold choice and functional form using two alternative clinical threshold schemes and simultaneous 4-df natural cubic splines for chloride and bicarbonate. We also conducted an exploratory outcome-driven threshold analysis by fitting separate constrained classification trees in MIMIC-IV for continuous chloride and bicarbonate, with a maximum of three terminal intervals and a minimum terminal-node size of 10%. The resulting cut points were locked and the derived 3 × 3 representation was applied to eICU without re-estimation. These outcome-derived cut points were interpreted only as prognostic sensitivity thresholds. Finally, a strict pre-exposure complete-case model included fixed admission features and only time-stamped covariates available up to completion of the paired exposure, excluding untimed or stay-level information on dynamic severity, ventilation, and renal replacement therapy.

We also examined first-day changes using an exploratory 24 h landmark design. Exact-time chloride-bicarbonate pairs within prespecified physiologic ranges (chloride 70–140 mmol/L and bicarbonate 5–45 mmol/L) were identified, with the earliest pair selected from 0 to 6 h and the latest pair from 12 to 24 h. The landmark cohort required continued ICU stay through 24 h and known in-hospital mortality. Low-low was defined as chloride <98 mmol/L plus bicarbonate <22 mmol/L, and transitions were classified as never, resolved, persistent, or incident. Transition models used parsimonious fixed/admission covariates to minimize adjustment for information generated during the transition window. Complementary continuous-change models included early chloride, early bicarbonate, changes in chloride and bicarbonate, and the same covariates. Timing-adjusted sensitivity models additionally accounted for the early measurement time and the interval between measurements. These analyses were exploratory and were not interpreted causally.

All tests were two-tailed, and *P* < 0.05 was considered statistically significant. Analyses were conducted using reproducible Python 3.13.5 scripts.

## 3 Results

### 3.1 Cohort construction and outcome availability

The MIMIC-IV v3.1 source cohort comprised 26,067 ICU admissions. After construction of the merged wide-format table, no duplicate subject, hospital-admission, or ICU-admission identifiers remained. Early chloride values were available for 25,947 ICU stays, bicarbonate values for 25,946 stays, and paired chloride and bicarbonate values for 25,842 stays. The final MIMIC-IV v3.1 analysis cohort therefore included 25,842 ICU stays. In-hospital death occurred in 4,216 patients, corresponding to a crude mortality rate of 16.31%. All paired chloride and bicarbonate values in MIMIC-IV were recorded at the same time point (Table 1; Figure 1; Supplementary Tables S1, S3).

**Table 1:** Cohort construction, pairing quality, and outcome availability.

Item	MIMIC-IV	eICU
Source/master ICU stays	26,067	42,633
Paired early chloride and bicarbonate values	25,842	38,115
Exact-time paired measurements	25,842 (100%)	37,902 (99.44%)
Selected pair time from ICU admission, h, median [IQR]	2.52 [1.01, 5.05]	5.83 [2.00, 11.38]
After analytic master linkage	—	37,454
Final in-hospital mortality analysis cohort	25,842	37,045
In-hospital deaths	4,216	5,741
Crude in-hospital mortality	16.31%	15.50%
Complete-case Model 3b, *n*	13,168	21,414
Complete-case Model 3b, events	2,370	3,907
PMM Model 3b, *n*	25,842	37,041
PMM Model 3b, events	4,216	5,741

Pair time is measured from ICU admission to the selected chloride-bicarbonate pair. For eICU, 38,115 paired stays yielded 37,454 analysis rows after linkage with the analytic master cohort; 409 lacked in-hospital mortality status, leaving 37,045 for the primary mortality analysis. The eICU PMM sample differs from the final outcome cohort by four ICU stays because of model-specific covariate processing. Model 3b was the prespecified primary multivariable model.

eICU, eICU Collaborative Research Database; ICU, intensive care unit; IQR, interquartile range; MIMIC-IV, Medical Information Mart for Intensive Care IV; PMM, predictive mean matching multiple imputation.

**Figure 1:**
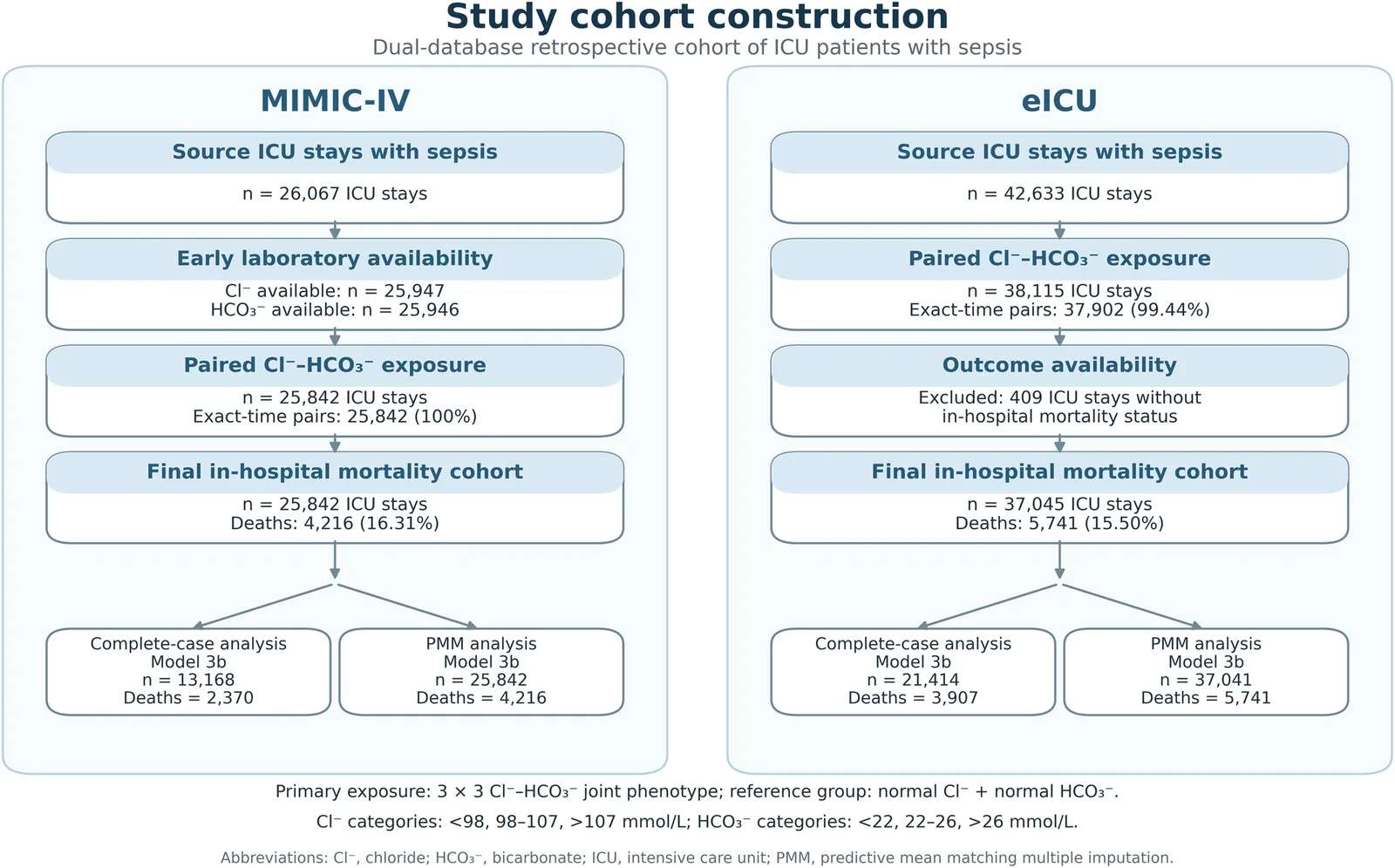
Study cohort construction. Flow diagram showing cohort assembly in MIMIC-IV v3.1 and eICU v2.0, early chloride and bicarbonate availability, pairing quality, outcome availability, and sample sizes for complete-case and predictive mean matching analyses. Cl⁻, chloride; $\mathrm{HC}O_{3}^{-}$ , bicarbonate; ICU, intensive care unit; PMM, predictive mean matching.

The eICU v2.0 master table contained 42,633 ICU admissions, of which 38,115 had early paired chloride and bicarbonate values. Among these, 37,902 (99.44%) had measurements recorded at the same time. After linkage with the analytic master cohort, 37,454 ICU stays remained; 409 lacked in-hospital mortality status, leaving 37,045 ICU stays in the final eICU v2.0 mortality cohort. There were 5,741 in-hospital deaths, corresponding to a crude mortality rate of 15.50% (Table 1; Supplementary Tables S2, S3). Median selected-pair times were 2.52 h [IQR 1.01–5.05] in MIMIC-IV and 5.83 h [IQR 2.00–11.38] in eICU (Table 1).

Missingness varied by variable and cohort. In MIMIC-IV, missingness was 61.70% for albumin, 42.89% for lactate, 36.16% for pH, and 36.19% for PaCO_2_. In the final eICU mortality cohort, missingness was 43.37% for pH, 43.33% for PaCO_2_, 35.51% for lactate, 26.81% for albumin, and 20.29% for anion gap. Complete-case Model 3b retained 51.0% of MIMIC-IV and 57.8% of eICU stays; predictive mean matching multiple imputation was therefore a key sensitivity analysis.

### 3.2 Crude mortality across chloride-bicarbonate phenotypes

Crude mortality varied across the nine phenotypes. In MIMIC-IV, mortality was highest in the low-chloride plus low-bicarbonate group (473/1,330; 35.56%), compared with 11.45% in the normal-normal reference group. The lowest mortality was observed in the high-chloride plus normal-bicarbonate group (7.08%) (Table 2; Figure 2; Supplementary Table S1).

**Table 2:** Crude in-hospital mortality across chloride-bicarbonate joint phenotypes.

Phenotype	MIMIC-IV, n	MIMIC-IV deaths	MIMIC-IV mortality (%)	eICU, n	eICU deaths	eICU mortality (%)
Low chloride + low bicarbonate	1,330	473	35.56	1,510	405	26.82
Low chloride + normal bicarbonate	1,177	273	23.19	1,628	248	15.23
Low chloride + high bicarbonate	1,021	203	19.88	2,416	330	13.66
Normal chloride + low bicarbonate	5,032	1,190	23.65	5,790	1,238	21.38
Normal chloride + normal bicarbonate	6,203	710	11.45	7,961	897	11.27
Normal chloride + high bicarbonate	2,203	266	12.07	5,617	624	11.11
High chloride + low bicarbonate	4,303	759	17.64	6,602	1,355	20.52
High chloride + normal bicarbonate	4,113	291	7.08	4,408	494	11.21
High chloride + high bicarbonate	460	51	11.09	1,113	150	13.48

Chloride categories: low <98 mmol/L, normal 98–107 mmol/L, and high >107 mmol/L. Bicarbonate categories: low <22 mmol/L, normal 22–26 mmol/L, and high >26 mmol/L.

eICU, eICU Collaborative Research Database; MIMIC-IV, Medical Information Mart for Intensive Care IV.

**Figure 2:**
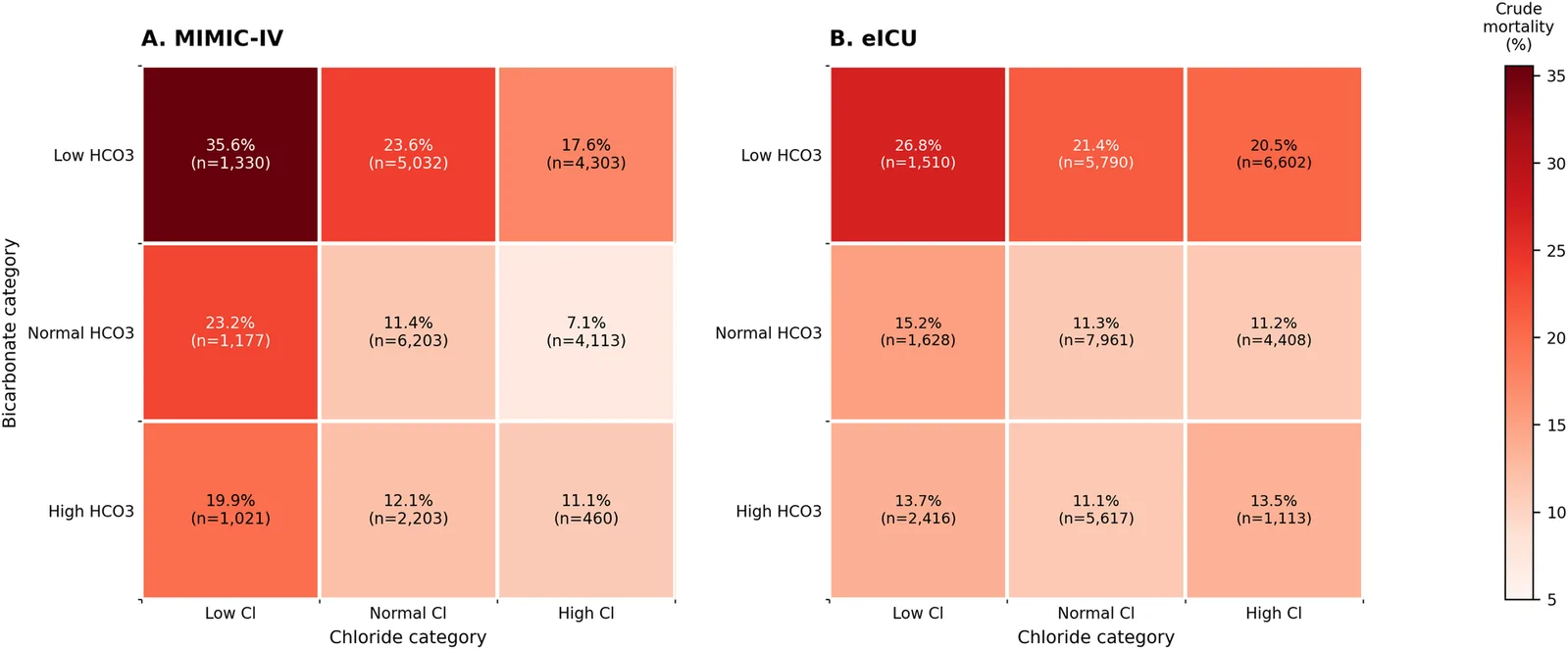
Crude in-hospital mortality across chloride-bicarbonate joint phenotypes in MIMIC-IV v3.1 and eICU v2.0. Heatmaps show crude mortality percentages for the nine combinations of chloride and bicarbonate categories; cell labels report mortality and sample size. Chloride was categorized as low (<98 mmol/L), normal (98–107 mmol/L), or high (>107 mmol/L), and bicarbonate as low (<22 mmol/L), normal (22–26 mmol/L), or high (>26 mmol/L).

A similar pattern was observed in eICU. The low-chloride plus low-bicarbonate group again had the highest crude mortality (405/1,510; 26.82%), compared with 11.27% in the reference group. The normal-chloride plus low-bicarbonate and high-chloride plus low-bicarbonate groups also had elevated crude mortality (Table 2; Supplementary Table S2).

### 3.3 Multiple imputation and multivariable analyses

In complete-case Model 3b, the 3 × 3 phenotype was globally associated with in-hospital mortality in both MIMIC-IV (*P* = 0.017) and eICU (*P* < 0.001).

However, the low-chloride plus low-bicarbonate contrast did not reach individual statistical significance in MIMIC-IV (OR 1.136, 95% CI 0.885–1.460; *P* = 0.317), whereas it was significant in eICU (OR 1.426, 95% CI 1.169–1.739; *P* < 0.001). Extended acid-base sensitivity models are reported in Supplementary Tables S4, S5.

Predictive mean matching generated 20 imputed datasets, and all fitted regression models converged. In both Model 3b and Model 4b-NoAG, the global phenotype association was significant in each cohort (all *P* < 0.001) (Supplementary Table S6; Figure S4).

In PMM Model 3b, low chloride plus low bicarbonate was associated with higher mortality relative to the normal-normal reference in both MIMIC-IV (OR 1.276, 95% CI 1.066–1.527) and eICU (OR 1.507, 95% CI 1.277–1.779) (Table 3; Supplementary Table S6). Low chloride plus normal bicarbonate was also associated with higher mortality in both cohorts, although several other cell-specific estimates differed in magnitude (Table 3; Supplementary Table S6).

**Table 3:** PMM model 3b associations between chloride-bicarbonate joint phenotype and in-hospital mortality.

Phenotype	MIMIC-IV OR (95% CI)	MIMIC-IV *P* value	eICU OR (95% CI)	eICU *P* value
Low chloride + low bicarbonate	1.276 (1.066–1.527)	0.008	1.507 (1.277–1.779)	<0.001
Low chloride + normal bicarbonate	1.487 (1.241–1.782)	<0.001	1.297 (1.094–1.537)	0.003
Low chloride + high bicarbonate	1.205 (0.992–1.464)	0.060	1.283 (1.107–1.487)	<0.001
Normal chloride + low bicarbonate	1.104 (0.977–1.248)	0.113	1.259 (1.127–1.406)	<0.001
Normal chloride + normal bicarbonate	Reference		Reference	
Normal chloride + high bicarbonate	0.899 (0.762–1.061)	0.209	1.044 (0.927–1.175)	0.479
High chloride + low bicarbonate	0.987 (0.865–1.126)	0.841	1.146 (1.029–1.275)	0.013
High chloride + normal bicarbonate	0.918 (0.781–1.080)	0.301	0.813 (0.715–0.924)	0.002
High chloride + high bicarbonate	1.032 (0.734–1.451)	0.858	0.921 (0.751–1.131)	0.434

The reference group was normal chloride plus normal bicarbonate. Estimates were pooled across 20 donor-based predictive mean matching imputed datasets using Rubin's rules. Model 3b was the prespecified primary multivariable model.

CI, confidence interval; eICU, eICU Collaborative Research Database; MIMIC-IV, Medical Information Mart for Intensive Care IV; OR, odds ratio; PMM, predictive mean matching.

### 3.4 CHBI analyses, pooled estimates, and interaction testing

CHBI provided complementary but less consistent information. In complete-case Model 3b, signed CHBI was associated with mortality in MIMIC-IV but not eICU, whereas absolute CHBI was associated with mortality in eICU but not MIMIC-IV. CHBI quartiles were globally associated with mortality in both cohorts (Supplementary Table S4; Figures S1–S3).

In the prespecified complete-case fixed-effect synthesis, low chloride plus low bicarbonate (pooled OR 1.306, 95% CI 1.118–1.526) and low chloride plus normal bicarbonate (pooled OR 1.444, 95% CI 1.221–1.708) were associated with higher mortality, whereas high chloride plus normal bicarbonate was associated with lower mortality (pooled OR 0.787, 95% CI 0.689–0.899) (Table 4; Figure 3).

**Table 4:** Complete-case pooled model 3b associations and between-cohort heterogeneity.

Phenotype	Pooled OR (95% CI)	*I*^2^ (%)	Heterogeneity *P* value
Low chloride + low bicarbonate	1.306 (1.118–1.526)	48.3	0.164
Low chloride + normal bicarbonate	1.444 (1.221–1.708)	0.0	0.639
Low chloride + high bicarbonate	1.104 (0.935–1.304)	0.0	0.359
Normal chloride + low bicarbonate	1.105 (0.993–1.229)	75.7	0.042
Normal chloride + high bicarbonate	1.041 (0.914–1.185)	33.8	0.219
High chloride + low bicarbonate	1.022 (0.919–1.137)	52.6	0.146
High chloride + normal bicarbonate	0.787 (0.689–0.899)	0.0	0.463
High chloride + high bicarbonate	0.974 (0.777–1.219)	12.5	0.285

Cohort-specific complete-case Model 3b estimates were combined using inverse-variance fixed-effect pooling. *I*^2^ should be interpreted cautiously because only two cohorts were available. A harmonized common-covariate heterogeneity analysis is provided in Supplementary Table S18.

CI, confidence interval; *I*^2^, percentage of total variation attributable to between-cohort heterogeneity; OR, odds ratio.

**Figure 3:**
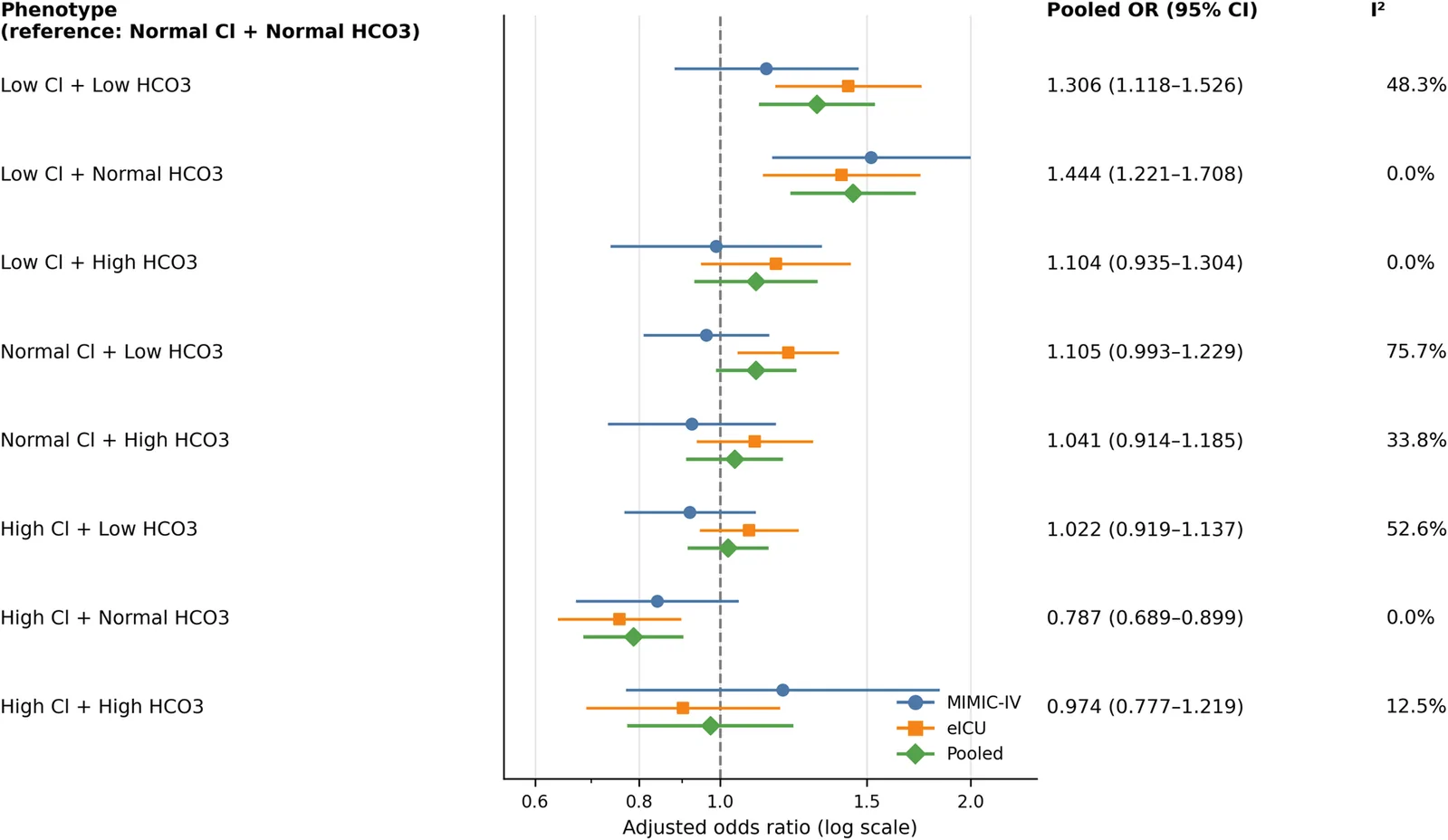
Cohort-specific and pooled associations between chloride-bicarbonate joint phenotypes and in-hospital mortality. Adjusted odds ratios and 95% confidence intervals are shown for MIMIC-IV v3.1, eICU v2.0, and inverse-variance fixed-effect pooled estimates. Normal chloride plus normal bicarbonate was the reference phenotype. CI, confidence interval; OR, odds ratio.

The original patient-level phenotype-by-cohort interaction analysis using database-specific adjustment blocks was not significant (*P* = 0.134). Because *I*^2^ was high for some phenotype cells, we added a harmonized common-covariate analysis. Under the harmonized adjustment, low chloride plus low bicarbonate had ORs of 1.43 in MIMIC-IV and 1.58 in eICU (pooled OR 1.52, 95% CI 1.31–1.75; *I*^2^ = 0%; cohort-difference *P* = 0.500), and normal chloride plus low bicarbonate also had *I*^2^ = 0%. Substantial *I*^2^ persisted in several low- and high-chloride cells; after false-discovery-rate correction, statistically significant cohort differences were confined to selected high-chloride cells. The global 8-df cross-cohort coefficient comparison remained significant (*P* < 0.001) (Supplementary Table S18). Formal 4-df categorical chloride-by-bicarbonate interaction tests were not significant in either MIMIC-IV (*P* = 0.095) or eICU (*P* = 0.109) (Supplementary Table S16).

### 3.5 Sensitivity analyses, external validation, and longitudinal findings

The common external validation cohort included 23,824 eICU stays and 4,305 deaths. The portable base model trained in MIMIC-IV had an AUROC of 0.7886 and a Brier score of 0.1234. Adding the clinical phenotype increased AUROC to 0.7896 (ΔAUROC 0.0010, 95% CI −0.0013 to 0.0031), reduced the Brier score to 0.1223 (ΔBrier −0.0011, 95% CI −0.0016 to −0.0006), and increased the calibration slope from 0.846 to 0.870. More flexible continuous representations likewise did not materially improve discrimination. IDI (−0.0035, 95% CI −0.0053 to −0.0017) and category-free NRI (−0.1149, 95% CI −0.1492 to −0.0826) did not support meaningful incremental predictive value, reinforcing that the phenotype is not a stand-alone prediction tool (Table 5; Figures 4, 5; Supplementary Tables S14, S19).

**Table 5:** External validation performance of MIMIC-IV v3.1-trained models in eICU v2.0.

Model	AUROC (95% CI)	Brier score (95% CI)	Calibration slope (95% CI)	Calibration intercept offset (95% CI)
Base	0.789 (0.781–0.795)	0.123 (0.121–0.126)	0.846 (0.816–0.878)	−0.200 (−0.240 to −0.162)
Chloride-bicarbonate phenotype	0.790 (0.782–0.796)	0.122 (0.120–0.125)	0.870 (0.840–0.904)	−0.159 (−0.198 to −0.122)
Change after adding phenotype	0.001 (−0.001 to 0.003)	−0.0011 (−0.0016 to −0.0006)	NA	NA

Portable common-variable models were trained in MIMIC-IV v3.1 and externally validated in the eICU v2.0 common validation cohort (*n* = 23,824; events = 4,305) without coefficient refitting. Uncertainty was estimated using 500 paired case-resampling bootstrap replicates of the validation cohort while the MIMIC-IV-trained coefficients were held fixed. A negative change in Brier score indicates lower prediction error. External NRI and IDI are reported in Supplementary Table S19.

AUROC, area under the receiver operating characteristic curve; CI, confidence interval; eICU, eICU Collaborative Research Database; MIMIC-IV, Medical Information Mart for Intensive Care IV.

**Figure 4:**
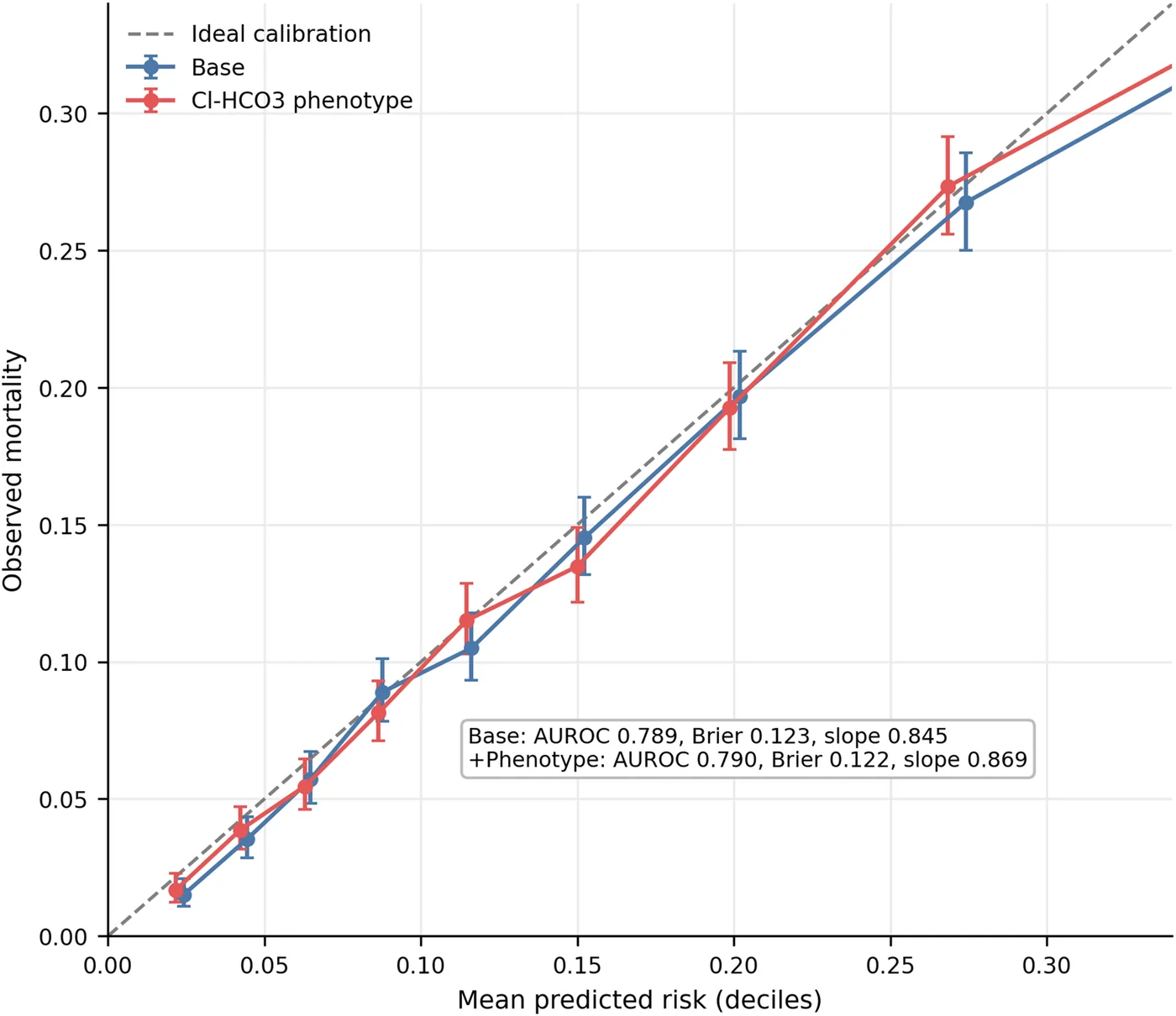
External calibration of MIMIC-IV v3.1-trained models in eICU v2.0. Observed mortality is plotted against mean predicted risk by decile for the portable base model and the model augmented with the chloride-bicarbonate phenotype. The dashed diagonal indicates ideal calibration, and error bars indicate 95% confidence intervals.

**Figure 5:**
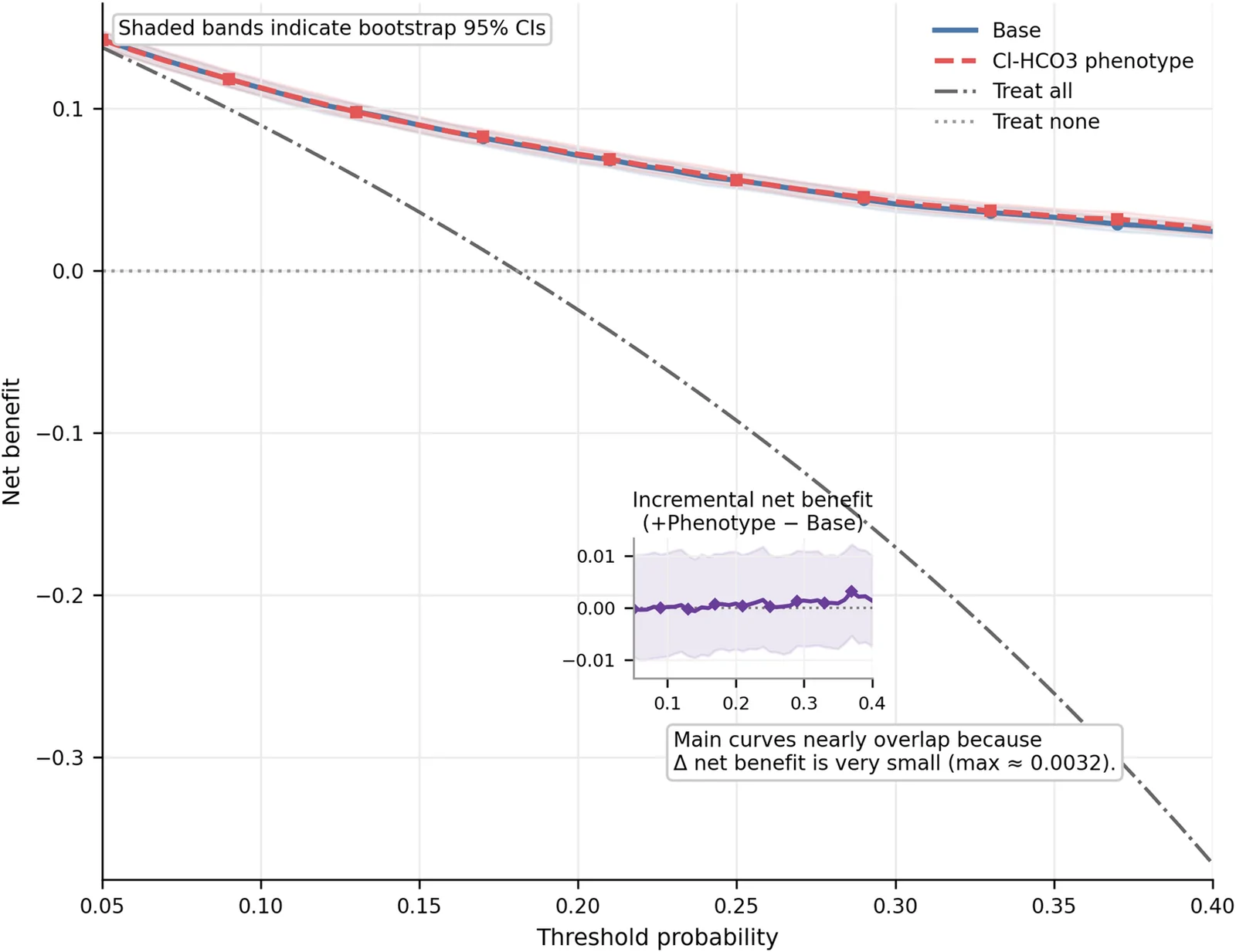
Decision-curve analysis of MIMIC-IV v3.1-trained models externally applied to eICU v2.0. Net benefit is shown across clinically relevant threshold probabilities for the portable base model, the phenotype-augmented model, and treat-all and treat-none strategies. The inset displays the incremental net benefit of the phenotype-augmented model relative to the base model.

Results were robust to alternative pairing rules, although associations were attenuated in earlier exposure-window analyses. Restricting eICU to the first eligible ICU stay per patient preserved both the global phenotype association (*n* = 19,173; 3,690 events; *P* < 0.001) and the low-low association (OR 1.483, 95% CI 1.208–1.822). In the 0–6 h window, the global phenotype test yielded *P* = 0.071 in MIMIC-IV and *P* = 0.014 in eICU, whereas the low-low contrast was not individually significant in either cohort. The global association remained significant in both cohorts in the 0–12 h window (Supplementary Figure S5). Prespecified subgroup analyses are presented in Supplementary Tables S8A,B and Supplementary Figure S6. Database-specific 28-day and 90-day analyses were also directionally consistent, with the caveat that eICU does not capture deaths after discharge (Table 6; Supplementary Tables S7A,B and S9A–C; Supplementary Figure S7).

**Table 6:** Sensitivity analyses of exposure definitions, patient-level restriction, and database-specific secondary mortality endpoints.

Cohort	Analysis or outcome	n	Events	Overall phenotype *P* value
MIMIC-IV	Primary analysis (0–24 h exposure window)	13,168	2,370	0.017
MIMIC-IV	Exact-time paired measurements only	13,168	2,370	0.017
MIMIC-IV	Paired measurements within 1 h	13,168	2,370	0.017
MIMIC-IV	Paired measurements within 3 h	13,168	2,370	0.017
MIMIC-IV	Exposure window: 0–6 h after ICU admission	11,321	2,034	0.071
MIMIC-IV	Exposure window: 0–12 h after ICU admission	12,775	2,289	0.036
eICU	Primary analysis (0–24 h exposure window)	21,414	3,907	<0.001
eICU	Exact-time paired measurements only	21,246	3,858	<0.001
eICU	Paired measurements within 1 h	21,295	3,875	<0.001
eICU	Paired measurements within 3 h	21,355	3,892	<0.001
eICU	Exposure window: 0–6 h after ICU admission	11,980	2,414	0.014
eICU	Exposure window: 0–12 h after ICU admission	17,249	3,296	<0.001
eICU	First ICU stay per unique patient	19,173	3,690	<0.001
Database-specific secondary mortality endpoints				
MIMIC-IV	In-hospital mortality	13,168	2,370	0.017
MIMIC-IV	Death within 28 days of hospital admission	13,168	2,462	0.014
MIMIC-IV	Death within 90 days of hospital admission	13,168	3,145	<0.001
eICU	In-hospital mortality	21,414	3,907	<0.001
eICU	In-hospital death by day 28 after hospital admission	21,414	3,747	<0.001
eICU	In-hospital death by day 90 after hospital admission	21,414	3,897	<0.001

Sensitivity analyses used complete-case Model 3b. Pairing interval indicates the maximum allowed absolute time difference between chloride and bicarbonate measurements. The eICU first-stay sensitivity retained only the first eligible ICU stay per unique patient. Because every MIMIC-IV pair retained in the primary cohort shared the same chart time, the primary, exact-time, ≤1 h, and ≤3 h analyses had identical samples and estimates. MIMIC-IV secondary inferential models used the recorded date of death relative to hospital admission. eICU inferential models captured deaths during the index hospitalization by day 28 or day 90 and did not include postdischarge deaths. Availability and event rates for 28-, 30-, 90-, and 365-day variables are provided in Supplementary Table S9A; the eICU variables are not equivalent to conventional postdischarge all-cause mortality. Records with missing eICU in-hospital mortality status were treated as missing for the derived day-specific endpoints; this correction did not change the complete-case Model 3b inferential samples or estimates.

eICU, eICU Collaborative Research Database; MIMIC-IV, Medical Information Mart for Intensive Care IV.

The phenotype remained globally associated with mortality under both alternative clinical threshold schemes in both cohorts; phenotype counts and crude mortality are shown in Supplementary Table S12A, and full cell-specific ORs are reported in Supplementary Table S12B. Simultaneous spline analyses supported nonlinearity, particularly for bicarbonate (Supplementary Table S13; Figures S8–S11). MIMIC-IV-derived constrained classification trees identified chloride split points of 97.5 and 101.5 mmol/L and bicarbonate split points of 17.5 and 21.5 mmol/L. Bootstrap resampling showed only moderate split-point stability, especially for the lower chloride threshold, supporting interpretation of the CART cut points as sample-dependent prognostic sensitivity thresholds. When locked and transported to eICU, the CART-derived 3 × 3 representation did not materially outperform the clinical 3 × 3 phenotype (ΔAUROC 0.0009, 95% CI −0.0010 to 0.0029; ΔBrier −0.00036, 95% CI −0.00075 to 0.00006) (Supplementary Tables S17A–C). In the strict pre-exposure model, low-low remained associated with higher mortality in MIMIC-IV (OR 1.700, 95% CI 1.143–2.528) and eICU (OR 1.787, 95% CI 1.480–2.158), with significant global phenotype tests in both cohorts (Supplementary Tables S15A,B).

In exploratory 24 h landmark analyses, 14,263 MIMIC-IV stays (2,414 deaths) and 8,295 eICU stays (1,553 deaths) had exact-time chloride-bicarbonate pairs in the 0–6 h and 12–24 h windows and remained in the ICU at 24 h. Compared with the never low-low category, persistent low-low status was associated with higher subsequent mortality in MIMIC-IV (OR 2.67, 95% CI 2.13–3.34) and eICU (OR 2.19, 95% CI 1.57–3.04). Incident low-low status was also associated with higher subsequent mortality, with ORs of 3.45 (95% CI 2.60–4.57) and 4.10 (95% CI 2.81–5.99), respectively. In continuous-change models, increasing bicarbonate was associated with lower subsequent mortality in both cohorts, whereas the association with chloride change was weaker and nonsignificant in MIMIC-IV but significant in eICU (Supplementary Tables S20A,B and S21A,B).

Timing-adjusted sensitivity models yielded similar conclusions across cohorts; all change estimates below are per cohort-specific 1-SD increase. In MIMIC-IV, the delta-chloride association remained attenuated (OR 0.949, 95% CI 0.899–1.002; *P* = 0.060), whereas increasing bicarbonate remained associated with lower subsequent mortality (OR 0.659, 95% CI 0.626–0.694; *P* < 0.001). In eICU, both delta chloride (OR 0.826, 95% CI 0.773–0.882; *P* < 0.001) and delta bicarbonate (OR 0.588, 95% CI 0.547–0.632; *P* < 0.001) remained inversely associated with subsequent mortality (Supplementary Table S21C).

### 3.6 Apparent performance of alternative chloride-bicarbonate representations

In direct Model 2b comparisons, the 3 × 3 phenotype contained more apparent prognostic information than CHBI, absolute CHBI, or CHBI quartiles, but less than retaining chloride and bicarbonate as separate continuous terms. Phenotype augmentation increased AUROC from 0.7344 to 0.7524 in MIMIC-IV, whereas continuous main effects plus their interaction reached 0.7593. In eICU, the corresponding AUROCs were 0.6208, 0.6617, and 0.6678. Adding chloride categories beyond bicarbonate improved fit in both cohorts (*P* = 0.008 and *P* < 0.001), whereas adding bicarbonate beyond chloride improved fit only in eICU (*P* = 0.571 and *P* < 0.001). The 4-df categorical interaction test remained nonsignificant. These findings support the 3 × 3 phenotype as an interpretable encoding rather than a statistically optimal or synergistic representation (Table 7; Supplementary Tables S10, S11 and S16).

**Table 7:** Selected direct model 2b comparisons of alternative chloride-bicarbonate representations.

Cohort	Model	n	Events	AUROC	ΔAUROC (95% CI)	Brier	ΔBrier (95% CI)	LRT P
MIMIC-IV	Baseline Model 2b	25,842	4,216	0.7344	—	0.1242	—	—
MIMIC-IV	+ chloride	25,842	4,216	0.7368	+0.0023 (+0.0006 to +0.0039)	0.1239	−0.0003 (−0.0005 to −0.0001)	<0.001
MIMIC-IV	+ bicarbonate	25,842	4,216	0.7523	+0.0179 (+0.0143 to +0.0216)	0.1215	−0.0027 (−0.0033 to −0.0021)	<0.001
MIMIC-IV	+ chloride+ bicarbonate	25,842	4,216	0.7579	+0.0235 (+0.0191 to +0.0278)	0.1206	−0.0036 (−0.0042 to −0.0029)	<0.001
MIMIC-IV	+ chloride + bicarbonate + interaction	25,842	4,216	0.7593	+0.0248 (+0.0204 to +0.0295)	0.1205	−0.0037 (−0.0044 to −0.0030)	<0.001
MIMIC-IV	+ CHBI	25,842	4,216	0.7380	+0.0036 (+0.0019 to +0.0054)	0.1237	−0.0005 (−0.0007 to −0.0002)	<0.001
MIMIC-IV	+ 3 × 3 joint phenotype	25,842	4,216	0.7524	+0.0180 (+0.0144 to +0.0218)	0.1219	−0.0023 (−0.0029 to −0.0018)	<0.001
eICU	Baseline Model 2b	37,041	5,741	0.6208	—	0.1280	—	—
eICU	+ chloride	37,041	5,741	0.6208	−0.0000 (−0.0001 to +0.0001)	0.1280	−0.0000 (−0.0000 to +0.0000)	0.843
eICU	+ bicarbonate	37,041	5,741	0.6624	+0.0416 (+0.0350 to +0.0481)	0.1244	−0.0036 (−0.0041 to −0.0031)	<0.001
eICU	+ chloride + bicarbonate	37,041	5,741	0.6654	+0.0446 (+0.0375 to +0.0512)	0.1240	−0.0040 (−0.0045 to −0.0034)	<0.001
eICU	+ chloride + bicarbonate + interaction	37,041	5,741	0.6678	+0.0470 (+0.0399 to +0.0538)	0.1239	−0.0041 (−0.0047 to −0.0036)	<0.001
eICU	+ CHBI	37,041	5,741	0.6390	+0.0182 (+0.0142 to +0.0226)	0.1268	−0.0012 (−0.0014 to −0.0009)	<0.001
eICU	+ 3 × 3 joint phenotype	37,041	5,741	0.6617	+0.0409 (+0.0345 to +0.0470)	0.1253	−0.0027 (−0.0031 to −0.0023)	<0.001

Δ values are relative to the cohort-specific Model 2b baseline. LRT *P* values compare each extension with baseline, except the continuous product-interaction model, which is compared with the two-main-effects model. Percentile 95% CIs are based on a fixed-prediction paired bootstrap with 500 case-resampling replicates and are not optimism corrected. Categorical incremental-information and interaction tests for the 3 × 3 framework are reported in Supplementary Table S16.

AUROC, area under the receiver operating characteristic curve; CHBI, chloride-bicarbonate index; CI, confidence interval; LRT, likelihood-ratio test.

## 4 Discussion

In this dual-database study, prespecified chloride-bicarbonate phenotypes were associated with in-hospital mortality in both databases among critically ill patients with sepsis. The low-low phenotype had the highest crude mortality. Although its cell-specific estimate was not individually significant in the MIMIC-IV complete-case primary model, the association was supported in eICU and by multiple-imputation and strict pre-exposure analyses in both cohorts. Additional analyses clarified the boundaries of these findings: formal categorical interaction tests did not provide evidence of chloride-by-bicarbonate interaction; data-driven cut points did not meaningfully outperform the clinical 3 × 3 thresholds in external validation; and NRI and IDI did not support meaningful incremental predictive value. In contrast, first-day landmark analyses showed that both persistent and incident low-low states were associated with higher subsequent mortality in both databases.

The contribution of this study is translational and incremental rather than the discovery of new biomarkers. Chloride and bicarbonate have each been associated with outcomes in sepsis in previous studies (4–9). Xiao et al. extended electrolyte phenotyping through K-means clustering of distributional features from seven electrolytes, deriving three phenotypes in MIMIC-IV and validating them in eICU and AmsterdamUMCdb (14). Our framework addresses a different use case: it deliberately restricts input to two physiologically linked measurements and fixed clinical categories so that the phenotype can be assigned without clustering, retraining, or latent-class interpretation.

This simplicity has both advantages and costs. The 3 × 3 phenotype is easy to communicate and preserves the direction of each component—low, normal, or high—but categorization discards within-category information, and the cut points are not statistically optimal. Consistent with this trade-off, continuous chloride and bicarbonate terms performed modestly better in the development samples, whereas MIMIC-IV-derived CART thresholds yielded only trivial numerical gains over the clinical cut points when locked and externally applied to eICU. The framework should therefore be judged primarily on interpretability and reproducibility rather than predictive superiority.

The low-low phenotype likely reflects a heterogeneous acid-base state rather than a single mechanism. Low bicarbonate may accompany lactic acidosis, renal dysfunction, impaired buffering, or gastrointestinal loss, whereas low chloride may reflect renal or gastrointestinal losses, diuretic exposure, volume status, or compensatory physiology. These mechanisms may coexist in sepsis. The observational design cannot determine which component is causal or whether either should be corrected solely because the phenotype is associated with high risk.

Cross-database heterogeneity also warrants nuanced interpretation. Some phenotype cells showed large *I*^2^ values; for example, normal chloride plus low bicarbonate was heterogeneous under the original cohort-specific adjustment sets. When both cohorts were refitted using the same common-covariate structure, heterogeneity for that cell and for the core low-low phenotype fell to *I*^2^ = 0%, indicating that the apparent heterogeneity was sensitive to the adjustment structure. However, substantial *I*^2^ persisted in several low- and high-chloride cells. After false-discovery-rate correction, statistically significant cohort differences were concentrated in selected high-chloride cells, and the global harmonized cross-cohort comparison remained significant. Differences in case mix, treatment, timing of laboratory testing, and unmeasured fluid exposure are plausible explanations, but these possibilities were not directly tested and should not be presented as established causes.

The interaction and threshold analyses help clarify what “joint” means in this study. Nested categorical tests showed that chloride categories added information beyond bicarbonate categories in both cohorts, but apparent discrimination gains were negligible and the 4-df categorical interaction test remained nonsignificant. The phenotype therefore should not be described as showing categorical synergy beyond separate main effects. Similarly, outcome-derived CART cut points were prognostic sensitivity thresholds rather than therapeutic boundaries. Their lack of a substantial external advantage supports retaining clinically familiar cut points for interpretability while recognizing that these thresholds are not biologically optimal.

External validation showed only modest incremental predictive value. Adding the phenotype to a portable MIMIC-IV-trained model left eICU AUROC essentially unchanged, although the Brier score and calibration slope improved modestly. NRI and IDI did not support improved discrimination or reclassification. Together with the parallel results for more flexible continuous encodings, these findings argue against treating the phenotype as a stand-alone prediction model or a major source of additional discrimination. Its more defensible role is descriptive risk characterization within a broader clinical assessment.

The longitudinal analyses extended the baseline phenotype assessment by incorporating repeat measurements during the first ICU day. In both databases, persistent and incident low-low states were associated with higher subsequent mortality, with little between-cohort heterogeneity for these contrasts. Changes in bicarbonate showed a consistent inverse association with subsequent mortality, whereas the chloride-change association was less consistent across cohorts. These findings suggest that repeated joint assessment may be informative as a descriptive strategy, but they do not establish that phenotype “resolution” is therapeutic. Trajectories and outcomes may be influenced by repeat testing, changing illness severity, fluid and drug exposures, and clinician responses.

Temporal robustness is important because the primary Model 3b intentionally incorporated peri-admission information and was not a real-time admission prediction model. The strict pre-exposure analysis preserved both the global phenotype association and the low-low signal in the two cohorts, reducing concern that the primary finding was driven entirely by post-exposure information. Multiple imputation, alternative clinical thresholds, and spline analyses also yielded supportive results. Collectively, these sensitivity analyses strengthen the evidence for an association but do not resolve residual confounding or causal ambiguity.

Subgroup findings should be considered hypothesis-generating. Most subgroup interaction tests did not show strong evidence of effect heterogeneity, and signals involving AKI, shock, or sodium bicarbonate therapy were not consistently reproduced across databases; one MIMIC-IV bicarbonate-therapy interaction could not be estimated reliably because of sparse data (Supplementary Tables S8A,B). These results should not be interpreted as evidence of phenotype-specific treatment effects.

The results should not be used to guide treatment based solely on chloride or bicarbonate categories. Sepsis guidelines and randomized evidence support selective, context-dependent use of sodium bicarbonate rather than routine correction of low bicarbonate, and fluid composition is only one determinant of chloride status (25–27). Our data identify associations and trajectories; they do not show that changing chloride, bicarbonate, or phenotype improves outcomes.

### 4.1 Clinical implications

At the bedside, the phenotype is best viewed as an interpretive prompt rather than a prediction or treatment rule. A low-low or evolving low-low pattern may prompt reassessment of renal function, lactate, volume status and fluid exposure, gastrointestinal losses, diuretic use, and other causes of acid-base disturbance. The study does not identify a chloride or bicarbonate level at which treatment should be initiated, nor does it show that correcting the biochemical abnormality improves survival. The practical value is that two routinely measured variables can be communicated as a direction-preserving phenotype without retraining a model.

### 4.2 Limitations

Several limitations should be acknowledged. First, this retrospective observational study is subject to residual confounding related to illness severity, fluid composition and cumulative chloride exposure, diuretic use, timing of renal replacement therapy, gastrointestinal losses, and clinician responses. Second, the primary Model 3b used peri-admission covariates that may overlap the exposure window; although the strict pre-exposure analysis yielded supportive findings, it cannot exclude temporal overadjustment or unmeasured confounding. The strict pre-exposure analysis was also restricted to a substantially smaller complete-case MIMIC-IV subset (*n* = 8,684 vs. 13,168 in the primary complete-case Model 3b), so differences in effect estimates may partly reflect sample selection. Third, the clinical cut points were selected for familiarity rather than biological optimality. The MIMIC-IV-derived CART analysis did not materially improve external performance, but the outcome-derived cut points were themselves sample-dependent and cannot define treatment thresholds. Fourth, the longitudinal analyses included only patients who survived and remained in the ICU through 24 h and underwent repeat testing, introducing potential landmark selection, informative measurement, and time-varying confounding; trajectories beyond the first day were not assessed. The transition models included only fixed/admission covariates and therefore did not fully adjust for progression of early illness severity. Fifth, complete-case Model 3b retained only 51.0% of eligible MIMIC-IV stays and 57.8% of eligible eICU stays; multiple-imputation findings remain model-dependent and cannot exclude nonrandom-missingness bias. Sixth, the primary eICU analysis did not explicitly model within-hospital correlation, although the first-stay sensitivity reduced repeated-patient dependence. Seventh, differences between databases in measurement, case mix, and variable definitions persisted despite harmonized adjustment, and both datasets are US-based, limiting geographic generalizability. Finally, the portable common-variable model did not show substantial improvement in external discrimination or reclassification, and the database-specific secondary mortality outcomes and development-sample Model 2b comparisons should be interpreted cautiously.

## 5 Conclusion

Prespecified chloride-bicarbonate joint phenotypes were associated with in-hospital mortality in both US ICU databases. Persistent and incident low-low states during the first ICU day were each associated with higher subsequent mortality. Although overall prediction error and calibration improved modestly, data-driven cut points did not meaningfully outperform the clinical thresholds, external discrimination was essentially unchanged, and reclassification metrics did not indicate improvement. The 3 × 3 framework should therefore be viewed as a transparent, direction-preserving phenotyping adjunct rather than a causal marker, therapeutic target, or stand-alone prediction model.

## Data Availability

Publicly available datasets were analyzed in this study. The datasets are available through the PhysioNet repository subject to credentialed access, completion of the required human-subjects research training, and acceptance of the applicable data use agreements. MIMIC-IV, version 3.1: https://physionet.org/content/mimiciv/3.1/ (DOI: 10.13026/kpb9-mt58). eICU Collaborative Research Database, version 2.0: https://physionet.org/content/eicu-crd/2.0/ (DOI: 10.13026/C2WM1R). No separate accession numbers are assigned; the database versions and version-specific DOIs serve as persistent identifiers.

## References

[1] Singer M Deutschman CS Seymour CW Shankar-Hari M Annane D Bauer M. The third international consensus definitions for sepsis and septic shock (sepsis-3). JAMA. (2016) 315:801–10. 10.1001/jama.2016.0287 26903338 PMC4968574

[2] Pfortmueller CA Uehlinger D Von Haehling S Schefold JC. Serum chloride levels in critical illness—the hidden story. Intensive Care Med Exp. (2018) 6:10. 10.1186/s40635-018-0174-5 29654387 PMC5899079

[3] Kellum JA. Determinants of blood pH in health and disease. Crit Care. (2000) 4:6–14. 10.1186/cc644 11094491 PMC137247

[4] Neyra JA Canepa-Escaro F Li X Manllo J Adams-Huet B Yee J. Association of hyperchloremia with hospital mortality in critically ill septic patients. Crit Care Med. (2015) 43:1938–44. 10.1097/CCM.0000000000001161 26154934 PMC4537691

[5] Oh HJ Kim SJ Kim YC Kim EJ Jung IY Oh DH. An increased chloride level in hypochloremia is associated with decreased mortality in patients with severe sepsis or septic shock. Sci Rep. (2017) 7:15883. 10.1038/s41598-017-16238-z 29162885 PMC5698440

[6] Suetrong B Pisitsak C Boyd JH Russell JA Walley KR. Hyperchloremia and moderate increase in serum chloride are associated with acute kidney injury in severe sepsis and septic shock patients. Crit Care. (2016) 20:315. 10.1186/s13054-016-1499-7 27716310 PMC5053142

[7] Zhao Z Huang F Shen J Lv W Huang Y Pan J. L-shape association between serum chloride and 365-day mortality in critically ill patients with sepsis based on the MIMIC-IV database. Sci Rep. (2025) 15:20391. 10.1038/s41598-025-08630-x 40595311 PMC12219897

[8] Lei M Ma M Zhang Y Hu N Xie J. Longitudinal serum chloride trajectories and their correlation with all-cause mortality in critically ill sepsis patients: a retrospective cohort analysis. BMC Infect Dis. (2025) 25:890. 10.1186/s12879-025-11238-1 40604613 PMC12224684

[9] Liang M Guan F Sun L Xi W Jia H Xu Y. The U-shaped relationship between serum bicarbonate levels and 28-day mortality in patients with sepsis: a retrospective cohort study of MIMIC-IV database. Dose Response. (2025) 23:15593258251374417. 10.1177/15593258251374417 40896300 PMC12394846

[10] Zampieri FG Bagshaw SM Semler MW. Fluid therapy for critically ill adults with sepsis: a review. JAMA. (2023) 329:1967–80. 10.1001/jama.2023.7560 37314271

[11] Semler MW Self WH Wanderer JP Ehrenfeld JM Wang L Byrne DW. Balanced crystalloids versus saline in critically ill adults. N Engl J Med. (2018) 378:829–39. 10.1056/NEJMoa1711584 29485925 PMC5846085

[12] Gunnerson KJ Saul M He S Kellum JA. Lactate versus non-lactate metabolic acidosis: a retrospective outcome evaluation of critically ill patients. Crit Care. (2006) 10:R22. 10.1186/cc3987 16507145 PMC1550830

[13] Seymour CW Kennedy JN Wang S Chang C-CH Elliott CF Xu Z. Derivation, validation, and potential treatment implications of novel clinical phenotypes for sepsis. JAMA. (2019) 321:2003–17. 10.1001/jama.2019.5791 31104070 PMC6537818

[14] Xiao W Huang L Guo H Liu W Zhang J Liu Y. Development and validation of potential phenotypes of serum electrolyte disturbances in critically ill patients and a web-based application. J Crit Care. (2024) 82:154793. 10.1016/j.jcrc.2024.154793 38548515

[15] Johnson A Bulgarelli L Pollard T Gow B Moody B Horng S. MIMIC-IV (version 3.1). PhysioNet. (2024). RRID:SCR_007345. 10.13026/kpb9-mt58

[16] Pollard T Johnson A Raffa J Celi LA Badawi O Mark R. eICU collaborative research database (version 2.0). PhysioNet. (2019). RRID:SCR_007345. 10.13026/C2WM1R PMC6132188 30204154

[17] Johnson AEW Bulgarelli L Shen L Gayles A Shammout A Horng S. MIMIC-IV, a freely accessible electronic health record dataset. Sci Data. (2023) 10:1. 10.1038/s41597-022-01899-x 36596836 PMC9810617

[18] Pollard TJ Johnson AEW Raffa JD Celi LA Mark RG Badawi O. The eICU collaborative research database, a freely available multi-center database for critical care research. Sci Data. (2018) 5:180178. 10.1038/sdata.2018.178 30204154 PMC6132188

[19] Benchimol EI Smeeth L Guttmann A Harron K Moher D Petersen I. The REporting of studies conducted using observational routinely-collected health data (RECORD) Statement. PLoS Med. (2015) 12:e1001885. 10.1371/journal.pmed.1001885 26440803 PMC4595218

[20] Rubin DB. Multiple Imputation for Nonresponse in Surveys. New York: John Wiley & Sons (1987). 10.1002/9780470316696

[21] Collins GS Reitsma JB Altman DG Moons KGM. Transparent reporting of a multivariable prediction model for individual prognosis or diagnosis (TRIPOD): the TRIPOD statement. Ann Intern Med. (2015) 162:55–63. 10.7326/M14-0697 25560714

[22] Van Calster B McLernon DJ Van Smeden M Wynants L Steyerberg EW. Calibration: the achilles heel of predictive analytics. BMC Med. (2019) 17:230. 10.1186/s12916-019-1466-7 31842878 PMC6912996

[23] Vickers AJ Van Calster B Steyerberg EW. Net benefit approaches to the evaluation of prediction models, molecular markers, and diagnostic tests. Br Med J. (2016) 352:i6. 10.1136/bmj.i6 PMC4724785 26810254

[24] Collins GS Moons KGM Dhiman P Riley RD Beam AL Van Calster B. TRIPOD + AI statement: updated guidance for reporting clinical prediction models that use regression or machine learning methods. Br Med J. (2024) 385:e078378. 10.1136/bmj-2023-078378 PMC11019967 38626948

[25] Brown RM Wang L Coston TD Krishnan NI Casey JD Wanderer JP. Balanced crystalloids versus saline in sepsis: a secondary analysis of the SMART clinical trial. Am J Respir Crit Care Med. (2019) 200:1487–95. 10.1164/rccm.201903-0557OC 31454263 PMC6909845

[26] Evans L Rhodes A Alhazzani W Antonelli M Coopersmith CM French C. Surviving sepsis campaign: international guidelines for management of sepsis and septic shock 2021. Intensive Care Med. (2021) 47:1181–247. 10.1007/s00134-021-06506-y 34599691 PMC8486643

[27] Jaber S Paugam C Futier E Lefrant J-Y Lasocki S Lescot T. Sodium bicarbonate therapy for patients with severe metabolic acidaemia in the intensive care unit (BICAR-ICU): a multicentre, open-label, randomised controlled, phase 3 trial. Lancet. (2018) 392:31–40. 10.1016/S0140-6736(18)31080-8 29910040

