# Supplementary material for: Association of early chloride-bicarbonate joint phenotypes with in-hospital mortality among intensive care unit patients with sepsis: a dual-database study
Source: Front Med (Lausanne). 2026 Sep 16;13:1947296. doi: 10.3389/fmed.2026.1947296 (PMC13624113; doi:10.3389/fmed.2026.1947296)
Supplement: Supplementary file 1 [file Supplementaryfile1.docx]

**Supplementary Material**

**Supplementary analytical methods**

**Model specifications.** For direct Model 2b representation comparisons, MIMIC-IV adjusted for age, sex, race, ICU care unit, admission type, and Charlson comorbidity index; eICU adjusted for age, sex, race, geographic region, ICU unit type, and Charlson comorbidity index. The portable MIMIC-IV-to-eICU base model used age, sex, Charlson comorbidity index, dynamic SOFA, creatinine, blood urea nitrogen, lactate, septic shock, AKI, invasive ventilation, sodium bicarbonate therapy, and vasoactive therapy. Continuous transport-model predictors were standardized using MIMIC-IV training parameters, and MIMIC-IV coefficients were applied to eICU without refitting.

**Strict pre-exposure specification.** Pair completion was the later of the selected chloride and bicarbonate measurement times. Fixed covariates were age, sex, race, ICU care unit, admission type, and Charlson comorbidity index in MIMIC-IV, and age, sex, race, geographic region, ICU unit type, and Charlson comorbidity index in eICU. In both cohorts, time-stamped creatinine, blood urea nitrogen, lactate, septic shock, AKI, sodium bicarbonate therapy, and vasoactive therapy were eligible only when recorded no later than pair completion; positive events or treatments with unknown timing were treated as missing. Untimed/stay-level dynamic severity, invasive ventilation, and renal replacement therapy were excluded. The original implementation and aggregate outputs are retained in the original reproducibility package and mirrored unchanged in the revision addendum.

**Categorical incremental information and interaction.** In complete-case Model 3b samples, nested models contained covariates plus chloride categories, bicarbonate categories, additive chloride-bicarbonate categorical main effects, or the full main-effects-plus-interaction parameterization. Two-degree-of-freedom likelihood-ratio tests quantified the incremental categorical contribution of chloride beyond bicarbonate and bicarbonate beyond chloride; a 4-df likelihood-ratio test assessed interaction terms beyond additive categorical main effects. These tests used the same cohort-specific Model 3b adjustment sets as the primary complete-case analyses.

**Harmonized heterogeneity.** Because database-specific Model 3b covariates differed, a harmonized sensitivity model used the same adjustment set in both cohorts: age, sex, harmonized race, Charlson index, SOFA, creatinine, blood urea nitrogen, lactate, septic shock, AKI, invasive ventilation, sodium bicarbonate therapy, and vasoactive therapy. Continuous covariates were standardized within cohort. Cell-specific cohort differences were tested on the log-OR scale and adjusted by the Benjamini-Hochberg FDR procedure; fixed-effect pooling and I² were descriptive because only two cohorts were available.

**Data-driven threshold sensitivity.** Separate constrained classification trees were fitted in MIMIC-IV for continuous chloride and bicarbonate with in-hospital mortality as the outcome, log-loss splitting, a maximum of three terminal intervals per biomarker, and a minimum terminal-node size of 10% of the derivation cohort. Split-point stability was examined with 500 bootstrap derivations. The MIMIC-derived split points were then locked before application to eICU and were not re-estimated in the validation cohort. The resulting 3 × 3 representation was compared with the prespecified clinical 3 × 3 phenotype in the fixed MIMIC-to-eICU transport framework. These outcome-derived cut points were treated as prognostic sensitivity thresholds, not therapeutic targets.

**External reclassification metrics.** For the prespecified portable base and phenotype-augmented models, MIMIC-IV coefficients and standardization parameters were applied to the eICU validation cohort without refitting. IDI and category-free NRI were calculated from the paired fixed predictions. Confidence intervals were obtained from 500 paired case-resampling bootstrap replicates of the eICU cohort while the prediction vectors were held fixed.

**Exploratory 24-hour longitudinal analysis.** Exact-time chloride-bicarbonate pairs within prespecified physiologic ranges (chloride 70–140 mmol/L and bicarbonate 5–45 mmol/L) were identified. The earliest pair at 0–6 hours and latest pair at 12–24 hours were selected. A 24-hour landmark required continued ICU stay through 24 hours and known in-hospital mortality. Low-low was defined as chloride <98 mmol/L plus bicarbonate <22 mmol/L and transitions were classified as never, resolved, persistent, or incident. Transition models used fixed/admission covariates only: age, sex, race, Charlson index, ICU unit and admission type in MIMIC-IV, and age, sex, race, Charlson index, region and ICU unit in eICU. Continuous-change models simultaneously included early chloride, early bicarbonate, delta chloride, and delta bicarbonate with the same covariates; delta was the 12–24-hour value minus the 0–6-hour value. Timing-adjusted sensitivity models additionally accounted for the early measurement time and the interval between measurements. These analyses were exploratory and subject to landmark selection, informative repeat testing, and time-varying confounding.

**Supplementary Tables**

General notes. Unless otherwise specified, continuous variables are presented as median [interquartile range] and categorical variables as n (%). All P values are two-sided. Chloride categories are low <98 mmol/L, normal 98–107 mmol/L, and high >107 mmol/L; bicarbonate categories are low <22 mmol/L, normal 22–26 mmol/L, and high >26 mmol/L. Vasoactive therapy was defined as administration of any prespecified vasopressor or inotropic agent during the database-specific peri-admission treatment window defined in the source derivation; the historical internal code field `vasopressor_any` represents this composite and is retained only for backward compatibility. The primary phenotype and CHBI were derived in the upstream exposure-construction step. The chloride and bicarbonate missingness columns in descriptive Tables 1–3 reflect downstream descriptive/QC recoding of continuous source variables, so their missingness can differ from the already-derived phenotype and CHBI variables; this descriptive recoding should not be interpreted as the analytic-sample definition for all continuous-representation models. Abbreviations: AIC, Akaike information criterion; AKI, acute kidney injury; APACHE IV, Acute Physiology and Chronic Health Evaluation IV; AUROC, area under the receiver operating characteristic curve; BIC, Bayesian information criterion; CART, classification and regression tree; CHBI, chloride-bicarbonate index; CI, confidence interval; Cl, chloride; eICU, eICU Collaborative Research Database; FDR, false discovery rate; HCO₃, bicarbonate; IDI, integrated discrimination improvement; ICU, intensive care unit; LRT, likelihood-ratio test; MIMIC-IV, Medical Information Mart for Intensive Care IV; NRI, net reclassification improvement; OASIS, Oxford Acute Severity of Illness Score; OR, odds ratio; PaCO₂, arterial partial pressure of carbon dioxide; PMM, predictive mean matching; SD, standard deviation; SMD, standardized mean difference; SOFA, Sequential Organ Failure Assessment; NE, not estimable.

**Supplementary Table 1.** Baseline characteristics of ICU patients with sepsis in MIMIC-IV v3.1 according to chloride-bicarbonate joint phenotypes.

| **Characteristic** | **Level** | **Overall** | **Low Cl + Low HCO₃** | **Low Cl + Normal HCO₃** | **Low Cl + High HCO₃** | **Normal Cl + Low HCO₃** | **Normal Cl + Normal HCO₃** | **Normal Cl + High HCO₃** | **High Cl + Low HCO₃** | **High Cl + Normal HCO₃** | **High Cl + High HCO₃** | **Max pairwise SMD** | **Missing, n** | **Missing, %** |
| --- | --- | --- | --- | --- | --- | --- | --- | --- | --- | --- | --- | --- | --- | --- |
| In-hospital mortality | Yes | 4216 (16.3%) | 473 (35.6%) | 273 (23.2%) | 203 (19.9%) | 1190 (23.6%) | 710 (11.4%) | 266 (12.1%) | 759 (17.6%) | 291 (7.1%) | 51 (11.1%) | 0.742 | 0 | 0.000 |
| Age, years |  | 66.71 [55.83, 76.74] | 63.71 [53.62, 73.71] | 64.88 [55.23, 75.06] | 67.24 [57.34, 77.08] | 66.79 [55.47, 76.91] | 66.50 [55.32, 76.58] | 66.60 [56.44, 77.94] | 67.48 [55.28, 77.92] | 67.53 [57.36, 76.37] | 65.81 [56.16, 75.16] | 0.234 | 0 | 0.000 |
| Male sex | Yes | 15286 (59.2%) | 813 (61.1%) | 676 (57.4%) | 546 (53.5%) | 2927 (58.2%) | 3840 (61.9%) | 1298 (58.9%) | 2226 (51.7%) | 2658 (64.6%) | 302 (65.7%) | 0.286 | 0 | 0.000 |
| Race/ethnicity | Asian | 755 (2.9%) | 51 (3.8%) | 35 (3.0%) | 14 (1.4%) | 175 (3.5%) | 151 (2.4%) | 46 (2.1%) | 158 (3.7%) | 118 (2.9%) | 7 (1.5%) | 0.244 | 0 | 0.000 |
|  | Black | 2201 (8.5%) | 136 (10.2%) | 116 (9.9%) | 118 (11.6%) | 457 (9.1%) | 523 (8.4%) | 196 (8.9%) | 382 (8.9%) | 238 (5.8%) | 35 (7.6%) |  |  |  |
|  | Hispanic | 900 (3.5%) | 45 (3.4%) | 47 (4.0%) | 40 (3.9%) | 170 (3.4%) | 207 (3.3%) | 71 (3.2%) | 166 (3.9%) | 141 (3.4%) | 13 (2.8%) |  |  |  |
|  | Other | 1114 (4.3%) | 59 (4.4%) | 57 (4.8%) | 41 (4.0%) | 217 (4.3%) | 286 (4.6%) | 92 (4.2%) | 200 (4.6%) | 149 (3.6%) | 13 (2.8%) |  |  |  |
|  | Unknown | 4175 (16.2%) | 247 (18.6%) | 169 (14.4%) | 150 (14.7%) | 895 (17.8%) | 1021 (16.5%) | 328 (14.9%) | 735 (17.1%) | 565 (13.7%) | 65 (14.1%) |  |  |  |
|  | White | 16697 (64.6%) | 792 (59.5%) | 753 (64.0%) | 658 (64.4%) | 3118 (62.0%) | 4015 (64.7%) | 1470 (66.7%) | 2662 (61.9%) | 2902 (70.6%) | 327 (71.1%) |  |  |  |
| ICU type/care unit | Cardiac Vascular Intensive Care Unit (CVICU) | 5318 (20.6%) | 55 (4.1%) | 70 (5.9%) | 55 (5.4%) | 420 (8.3%) | 1099 (17.7%) | 228 (10.3%) | 924 (21.5%) | 2294 (55.8%) | 173 (37.6%) | 1.365 | 0 | 0.000 |
|  | Coronary Care Unit (CCU) | 2221 (8.6%) | 201 (15.1%) | 167 (14.2%) | 144 (14.1%) | 530 (10.5%) | 536 (8.6%) | 241 (10.9%) | 258 (6.0%) | 125 (3.0%) | 19 (4.1%) |  |  |  |
|  | Medical Intensive Care Unit (MICU) | 6266 (24.2%) | 551 (41.4%) | 401 (34.1%) | 377 (36.9%) | 1407 (28.0%) | 1222 (19.7%) | 625 (28.4%) | 1070 (24.9%) | 508 (12.4%) | 105 (22.8%) |  |  |  |
|  | Medical/Surgical Intensive Care Unit (MICU/SICU) | 4484 (17.4%) | 240 (18.0%) | 237 (20.1%) | 220 (21.5%) | 1003 (19.9%) | 1030 (16.6%) | 402 (18.2%) | 863 (20.1%) | 417 (10.1%) | 72 (15.7%) |  |  |  |
|  | Neuro Intermediate | 661 (2.6%) | 12 (0.9%) | 37 (3.1%) | 16 (1.6%) | 144 (2.9%) | 275 (4.4%) | 35 (1.6%) | 78 (1.8%) | 58 (1.4%) | 6 (1.3%) |  |  |  |
|  | Neuro Surgical Intensive Care Unit (Neuro SICU) | 419 (1.6%) | 18 (1.4%) | 23 (2.0%) | 11 (1.1%) | 106 (2.1%) | 128 (2.1%) | 24 (1.1%) | 76 (1.8%) | 32 (0.8%) | 1 (0.2%) |  |  |  |
|  | Other | 317 (1.2%) | 16 (1.2%) | 19 (1.6%) | 12 (1.2%) | 85 (1.7%) | 111 (1.8%) | 21 (1.0%) | 35 (0.8%) | 15 (0.4%) | 3 (0.7%) |  |  |  |
|  | Surgical Intensive Care Unit (SICU) | 3328 (12.9%) | 146 (11.0%) | 141 (12.0%) | 110 (10.8%) | 696 (13.8%) | 1050 (16.9%) | 332 (15.1%) | 459 (10.7%) | 351 (8.5%) | 43 (9.3%) |  |  |  |
|  | Trauma SICU (TSICU) | 2828 (10.9%) | 91 (6.8%) | 82 (7.0%) | 76 (7.4%) | 641 (12.7%) | 752 (12.1%) | 295 (13.4%) | 540 (12.5%) | 313 (7.6%) | 38 (8.3%) |  |  |  |
| Admission type | DIRECT EMER. | 881 (3.4%) | 48 (3.6%) | 38 (3.2%) | 40 (3.9%) | 172 (3.4%) | 224 (3.6%) | 94 (4.3%) | 106 (2.5%) | 141 (3.4%) | 18 (3.9%) | 0.780 | 0 | 0.000 |
|  | ELECTIVE | 1179 (4.6%) | 3 (0.2%) | 7 (0.6%) | 10 (1.0%) | 80 (1.6%) | 273 (4.4%) | 71 (3.2%) | 247 (5.7%) | 461 (11.2%) | 27 (5.9%) |  |  |  |
|  | EW EMER. | 12847 (49.7%) | 717 (53.9%) | 606 (51.5%) | 537 (52.6%) | 2757 (54.8%) | 3052 (49.2%) | 1144 (51.9%) | 2437 (56.6%) | 1416 (34.4%) | 181 (39.3%) |  |  |  |
|  | OBSERVATION ADMIT | 2799 (10.8%) | 212 (15.9%) | 217 (18.4%) | 137 (13.4%) | 786 (15.6%) | 759 (12.2%) | 158 (7.2%) | 338 (7.9%) | 177 (4.3%) | 15 (3.3%) |  |  |  |
|  | Other | 28 (0.1%) | 0 (0.0%) | 0 (0.0%) | 0 (0.0%) | 7 (0.1%) | 10 (0.2%) | 2 (0.1%) | 7 (0.2%) | 2 (0.0%) | 0 (0.0%) |  |  |  |
|  | SURGICAL SAME DAY ADMISSION | 2609 (10.1%) | 22 (1.7%) | 31 (2.6%) | 24 (2.4%) | 193 (3.8%) | 583 (9.4%) | 170 (7.7%) | 371 (8.6%) | 1118 (27.2%) | 97 (21.1%) |  |  |  |
|  | URGENT | 5499 (21.3%) | 328 (24.7%) | 278 (23.6%) | 273 (26.7%) | 1037 (20.6%) | 1302 (21.0%) | 564 (25.6%) | 797 (18.5%) | 798 (19.4%) | 122 (26.5%) |  |  |  |
| Charlson comorbidity index |  | 5.00 [3.00, 7.00] | 6.00 [4.00, 8.00] | 5.00 [4.00, 8.00] | 6.00 [4.00, 8.00] | 5.00 [3.00, 8.00] | 5.00 [3.00, 7.00] | 5.00 [3.00, 7.00] | 5.00 [3.00, 7.00] | 4.00 [2.00, 6.00] | 4.00 [3.00, 6.00] | 0.648 | 0 | 0.000 |
| APS III |  | 45.00 [34.00, 61.00] | 66.00 [52.00, 85.00] | 52.00 [40.00, 64.00] | 48.00 [38.00, 60.00] | 53.00 [41.00, 70.00] | 40.00 [31.00, 53.00] | 42.00 [33.00, 55.00] | 49.00 [36.00, 66.00] | 35.00 [27.00, 50.00] | 40.00 [29.00, 56.25] | 1.280 | 0 | 0.000 |
| SAPS II |  | 38.00 [30.00, 48.00] | 49.00 [39.00, 60.00] | 38.00 [31.00, 46.00] | 37.00 [30.00, 45.00] | 42.00 [33.00, 53.00] | 35.00 [27.00, 42.00] | 35.00 [28.00, 43.00] | 41.00 [32.00, 52.00] | 36.00 [29.00, 43.00] | 36.00 [30.00, 45.00] | 1.016 | 0 | 0.000 |
| Maximum dynamic SOFA |  | 5.00 [3.00, 8.00] | 8.00 [5.00, 11.00] | 5.00 [3.00, 8.00] | 5.00 [3.00, 7.00] | 6.00 [4.00, 9.00] | 4.00 [3.00, 6.00] | 4.00 [3.00, 6.00] | 6.00 [4.00, 8.00] | 5.00 [4.00, 7.00] | 5.00 [4.00, 6.00] | 1.049 | 23 | 0.090 |
| OASIS |  | 33.00 [28.00, 39.00] | 36.00 [29.00, 44.00] | 32.00 [27.00, 38.00] | 33.00 [28.00, 39.00] | 35.00 [29.00, 41.00] | 32.00 [27.00, 38.00] | 33.00 [28.00, 38.00] | 35.00 [29.00, 41.00] | 33.00 [28.00, 38.00] | 34.00 [29.00, 38.00] | 0.461 | 0 | 0.000 |
| Septic shock | Yes | 6451 (25.0%) | 414 (31.1%) | 188 (16.0%) | 106 (10.4%) | 1401 (27.8%) | 987 (15.9%) | 215 (9.8%) | 1525 (35.4%) | 1504 (36.6%) | 111 (24.1%) | 0.670 | 0 | 0.000 |
| AKI | Yes | 20136 (77.9%) | 1180 (88.7%) | 976 (82.9%) | 848 (83.1%) | 4118 (81.8%) | 4740 (76.4%) | 1742 (79.1%) | 3239 (75.3%) | 2951 (71.7%) | 342 (74.3%) | 0.436 | 0 | 0.000 |
| Hypertension | Yes | 10655 (41.2%) | 377 (28.3%) | 406 (34.5%) | 372 (36.4%) | 1791 (35.6%) | 2750 (44.3%) | 933 (42.4%) | 1657 (38.5%) | 2147 (52.2%) | 222 (48.3%) | 0.501 | 0 | 0.000 |
| Diabetes | Yes | 8007 (31.0%) | 537 (40.4%) | 429 (36.4%) | 405 (39.7%) | 1713 (34.0%) | 1815 (29.3%) | 688 (31.2%) | 1216 (28.3%) | 1068 (26.0%) | 136 (29.6%) | 0.310 | 0 | 0.000 |
| Renal disease | Yes | 5463 (21.1%) | 466 (35.0%) | 370 (31.4%) | 291 (28.5%) | 1309 (26.0%) | 1033 (16.7%) | 433 (19.7%) | 999 (23.2%) | 516 (12.5%) | 46 (10.0%) | 0.628 | 0 | 0.000 |
| Congestive heart failure | Yes | 7034 (27.2%) | 472 (35.5%) | 420 (35.7%) | 517 (50.6%) | 1415 (28.1%) | 1585 (25.6%) | 791 (35.9%) | 925 (21.5%) | 804 (19.5%) | 105 (22.8%) | 0.689 | 0 | 0.000 |
| Chronic pulmonary disease | Yes | 6512 (25.2%) | 302 (22.7%) | 331 (28.1%) | 511 (50.0%) | 1115 (22.2%) | 1496 (24.1%) | 873 (39.6%) | 869 (20.2%) | 869 (21.1%) | 146 (31.7%) | 0.658 | 0 | 0.000 |
| Malignant cancer | Yes | 3587 (13.9%) | 222 (16.7%) | 198 (16.8%) | 156 (15.3%) | 903 (17.9%) | 915 (14.8%) | 321 (14.6%) | 534 (12.4%) | 298 (7.2%) | 40 (8.7%) | 0.327 | 0 | 0.000 |
| Chloride, mmol/L |  | 105.00 [101.00, 109.00] | 94.00 [90.00, 96.00] | 95.00 [92.00, 96.00] | 94.00 [91.00, 96.00] | 104.00 [101.00, 106.00] | 104.00 [101.00, 106.00] | 102.00 [100.00, 104.00] | 111.00 [109.00, 114.00] | 110.00 [109.00, 112.00] | 110.00 [108.00, 111.00] | 4.894 | 12 | 0.050 |
| Bicarbonate, mmol/L |  | 22.00 [20.00, 25.00] | 18.00 [15.00, 20.00] | 24.00 [23.00, 25.00] | 30.00 [28.00, 33.00] | 19.00 [17.00, 20.00] | 24.00 [23.00, 25.00] | 28.00 [27.00, 30.00] | 19.00 [16.25, 20.00] | 23.00 [22.00, 25.00] | 28.00 [27.00, 29.00] | 3.986 | 22 | 0.090 |
| CHBI |  | 0.08 [-0.87, 0.93] | -0.79 [-1.22, -0.16] | -1.89 [-2.32, -1.49] | -3.28 [-4.25, -2.67] | 0.58 [0.16, 1.01] | -0.52 [-0.87, -0.17] | -1.70 [-2.16, -1.23] | 1.73 [1.30, 2.37] | 0.55 [0.26, 0.90] | -0.44 [-0.70, -0.20] | 4.979 | 0 | 0.000 |
| Abs-CHBI |  | 0.90 [0.42, 1.61] | 0.85 [0.45, 1.35] | 1.89 [1.49, 2.32] | 3.28 [2.67, 4.25] | 0.58 [0.29, 1.01] | 0.52 [0.23, 0.87] | 1.70 [1.23, 2.16] | 1.73 [1.30, 2.37] | 0.55 [0.30, 0.90] | 0.50 [0.24, 0.71] | 3.234 | 0 | 0.000 |
| pH |  | 7.38 [7.31, 7.43] | 7.32 [7.22, 7.39] | 7.41 [7.34, 7.46] | 7.41 [7.33, 7.47] | 7.33 [7.26, 7.40] | 7.39 [7.35, 7.44] | 7.40 [7.34, 7.45] | 7.35 [7.27, 7.40] | 7.40 [7.36, 7.43] | 7.40 [7.36, 7.44] | 0.848 | 9344 | 36.160 |
| PaCO₂, mmHg |  | 40.00 [35.00, 46.00] | 35.00 [29.00, 43.00] | 40.00 [35.00, 47.00] | 50.00 [42.00, 66.00] | 37.00 [32.00, 43.00] | 41.00 [37.00, 46.00] | 48.00 [41.00, 57.00] | 38.00 [33.00, 43.00] | 41.00 [38.00, 45.00] | 45.00 [41.00, 50.00] | 1.173 | 9351 | 36.190 |
| Lactate, mmol/L |  | 1.60 [1.10, 2.50] | 3.10 [1.70, 6.50] | 1.70 [1.20, 2.80] | 1.40 [1.00, 2.10] | 2.20 [1.40, 4.20] | 1.50 [1.10, 2.10] | 1.30 [1.00, 1.80] | 1.80 [1.20, 3.00] | 1.30 [1.00, 1.80] | 1.30 [1.00, 1.70] | 0.976 | 11083 | 42.890 |
| Anion gap |  | 14.00 [12.00, 17.00] | 21.00 [17.00, 25.00] | 17.00 [14.00, 19.00] | 14.00 [12.00, 16.00] | 17.00 [14.00, 20.00] | 14.00 [12.00, 16.00] | 12.00 [11.00, 14.00] | 14.00 [12.00, 17.00] | 11.00 [10.00, 13.00] | 10.00 [9.00, 12.00] | 2.106 | 43 | 0.170 |
| Albumin, g/dL |  | 3.00 [2.60, 3.50] | 2.90 [2.50, 3.50] | 3.10 [2.60, 3.50] | 3.20 [2.80, 3.60] | 3.00 [2.50, 3.40] | 3.20 [2.70, 3.60] | 3.20 [2.70, 3.60] | 2.80 [2.40, 3.20] | 3.00 [2.50, 3.40] | 2.90 [2.48, 3.30] | 0.708 | 15945 | 61.700 |
| Creatinine, mg/dL |  | 1.00 [0.80, 1.60] | 2.40 [1.30, 4.60] | 1.30 [0.80, 2.50] | 1.10 [0.70, 1.80] | 1.30 [0.90, 2.20] | 0.90 [0.70, 1.30] | 0.90 [0.70, 1.30] | 1.10 [0.80, 1.70] | 0.90 [0.70, 1.10] | 0.90 [0.70, 1.10] | 1.131 | 12 | 0.050 |
| BUN, mg/dL |  | 20.00 [14.00, 34.00] | 43.00 [24.00, 72.00] | 26.00 [15.00, 45.00] | 25.00 [15.00, 41.00] | 25.00 [16.00, 44.00] | 18.00 [13.00, 26.00] | 19.00 [13.00, 29.00] | 21.00 [14.00, 37.00] | 17.00 [13.00, 23.00] | 19.00 [14.00, 29.00] | 1.103 | 12 | 0.050 |
| Invasive ventilation | Yes | 15651 (60.6%) | 730 (54.9%) | 577 (49.0%) | 527 (51.6%) | 2845 (56.5%) | 3488 (56.2%) | 1245 (56.5%) | 2838 (66.0%) | 3061 (74.4%) | 340 (73.9%) | 0.541 | 0 | 0.000 |
| Any vasoactive therapy | Yes | 13209 (51.1%) | 750 (56.4%) | 451 (38.3%) | 320 (31.3%) | 2728 (54.2%) | 2596 (41.9%) | 677 (30.7%) | 2611 (60.7%) | 2830 (68.8%) | 246 (53.5%) | 0.824 | 0 | 0.000 |
| Sodium bicarbonate therapy | Yes | 2230 (8.6%) | 334 (25.1%) | 46 (3.9%) | 24 (2.4%) | 743 (14.8%) | 160 (2.6%) | 61 (2.8%) | 720 (16.7%) | 128 (3.1%) | 14 (3.0%) | 0.701 | 0 | 0.000 |
| RRT | Yes | 2639 (10.2%) | 475 (35.7%) | 266 (22.6%) | 153 (15.0%) | 763 (15.2%) | 381 (6.1%) | 129 (5.9%) | 342 (7.9%) | 116 (2.8%) | 14 (3.0%) | 0.918 | 0 | 0.000 |
| Fluid input, mL |  | 4962.49 [2698.06, 7821.82] | 4062.10 [2235.06, 7447.94] | 3027.71 [1643.50, 5083.47] | 2420.93 [1327.97, 4377.49] | 5041.00 [2837.52, 8137.70] | 4310.00 [2444.76, 6769.39] | 3181.04 [1797.55, 5407.86] | 6743.50 [4084.34, 9968.38] | 6560.92 [4285.20, 8628.01] | 5668.29 [3305.04, 8210.97] | 0.935 | 2103 | 8.140 |
| Urine output, mL |  | 1540.00 [920.00, 2350.00] | 830.00 [238.00, 1810.00] | 1428.50 [756.75, 2298.75] | 1577.00 [922.50, 2520.00] | 1290.00 [691.75, 2150.00] | 1580.00 [1015.00, 2340.00] | 1585.00 [1015.00, 2375.00] | 1550.00 [895.00, 2385.00] | 1815.00 [1270.00, 2525.00] | 1782.00 [1235.00, 2535.00] | 0.514 | 613 | 2.370 |

*Note.* The table includes 25,842 eligible MIMIC-IV ICU stays with nonmissing in-hospital mortality status, including 4,216 in-hospital deaths. Continuous variables are presented as median [interquartile range] and categorical variables as n (%). Maximum pairwise SMD is the largest absolute pairwise standardized mean difference across the nine phenotype groups.

**Supplementary Table 2.** Cohort characteristics of the final eICU v2.0 analysis cohort according to chloride-bicarbonate joint phenotypes.

| **Characteristic** | **Level** | **Overall** | **Low Cl + Low HCO₃** | **Low Cl + Normal HCO₃** | **Low Cl + High HCO₃** | **Normal Cl + Low HCO₃** | **Normal Cl + Normal HCO₃** | **Normal Cl + High HCO₃** | **High Cl + Low HCO₃** | **High Cl + Normal HCO₃** | **High Cl + High HCO₃** | **Maximum pairwise SMD** | **Missing, n** | **Missing, %** |
| --- | --- | --- | --- | --- | --- | --- | --- | --- | --- | --- | --- | --- | --- | --- |
| In-hospital mortality | Yes | 5741 (15.5%) | 405 (26.8%) | 248 (15.2%) | 330 (13.7%) | 1238 (21.4%) | 897 (11.3%) | 624 (11.1%) | 1355 (20.5%) | 494 (11.2%) | 150 (13.5%) | 0.409 | 0 | 0.0 |
| Age, years |  | 67.00 [55.00, 78.00] | 62.00 [52.00, 72.00] | 65.00 [54.00, 76.00] | 67.00 [57.00, 76.25] | 65.00 [54.00, 76.00] | 66.00 [54.00, 77.00] | 68.00 [57.00, 78.00] | 67.00 [54.00, 79.00] | 68.00 [55.00, 79.00] | 70.00 [56.00, 80.00] | 0.364 | 0 | 0.0 |
| Male sex | Yes | 19236 (51.9%) | 832 (55.1%) | 899 (55.2%) | 1218 (50.4%) | 2939 (50.8%) | 4384 (55.1%) | 2962 (52.7%) | 3064 (46.4%) | 2327 (52.8%) | 611 (54.9%) | 0.177 | 4 | 0.01 |
| Race/ethnicity | African American | 3878 (10.5%) | 160 (10.6%) | 215 (13.2%) | 202 (8.4%) | 637 (11.0%) | 810 (10.2%) | 544 (9.7%) | 760 (11.5%) | 431 (9.8%) | 119 (10.7%) | 0.157 | 0 | 0.0 |
|  | Asian | 550 (1.5%) | 22 (1.5%) | 34 (2.1%) | 23 (1.0%) | 85 (1.5%) | 118 (1.5%) | 48 (0.9%) | 138 (2.1%) | 74 (1.7%) | 8 (0.7%) |  |  |  |
|  | Caucasian | 28848 (77.9%) | 1166 (77.2%) | 1222 (75.1%) | 1968 (81.5%) | 4537 (78.4%) | 6239 (78.4%) | 4501 (80.1%) | 4961 (75.1%) | 3377 (76.6%) | 877 (78.8%) |  |  |  |
|  | Hispanic | 1844 (5.0%) | 57 (3.8%) | 69 (4.2%) | 121 (5.0%) | 217 (3.7%) | 411 (5.2%) | 287 (5.1%) | 353 (5.3%) | 278 (6.3%) | 51 (4.6%) |  |  |  |
|  | Native American | 290 (0.8%) | 23 (1.5%) | 19 (1.2%) | 14 (0.6%) | 56 (1.0%) | 60 (0.8%) | 27 (0.5%) | 53 (0.8%) | 34 (0.8%) | 4 (0.4%) |  |  |  |
|  | Unknown | 1635 (4.4%) | 82 (5.4%) | 69 (4.2%) | 88 (3.6%) | 258 (4.5%) | 323 (4.1%) | 210 (3.7%) | 337 (5.1%) | 214 (4.9%) | 54 (4.9%) |  |  |  |
| ICU type/care unit | CCU-CTICU | 2273 (6.1%) | 90 (6.0%) | 98 (6.0%) | 160 (6.6%) | 380 (6.6%) | 521 (6.5%) | 347 (6.2%) | 381 (5.8%) | 241 (5.5%) | 55 (4.9%) | 0.214 | 0 | 0.0 |
|  | CSICU | 628 (1.7%) | 20 (1.3%) | 18 (1.1%) | 23 (1.0%) | 89 (1.5%) | 137 (1.7%) | 99 (1.8%) | 126 (1.9%) | 97 (2.2%) | 19 (1.7%) |  |  |  |
|  | CTICU | 501 (1.4%) | 14 (0.9%) | 19 (1.2%) | 22 (0.9%) | 70 (1.2%) | 121 (1.5%) | 87 (1.5%) | 75 (1.1%) | 72 (1.6%) | 21 (1.9%) |  |  |  |
|  | Cardiac ICU | 2834 (7.7%) | 99 (6.6%) | 141 (8.7%) | 196 (8.1%) | 426 (7.4%) | 586 (7.4%) | 462 (8.2%) | 498 (7.5%) | 325 (7.4%) | 101 (9.1%) |  |  |  |
|  | MICU | 3967 (10.7%) | 220 (14.6%) | 200 (12.3%) | 280 (11.6%) | 733 (12.7%) | 818 (10.3%) | 540 (9.6%) | 729 (11.0%) | 346 (7.8%) | 101 (9.1%) |  |  |  |
|  | Med-Surg ICU | 23977 (64.7%) | 978 (64.8%) | 1042 (64.0%) | 1582 (65.5%) | 3687 (63.7%) | 5014 (63.0%) | 3646 (64.9%) | 4296 (65.1%) | 2981 (67.6%) | 751 (67.5%) |  |  |  |
|  | Neuro ICU | 1194 (3.2%) | 30 (2.0%) | 49 (3.0%) | 68 (2.8%) | 150 (2.6%) | 331 (4.2%) | 191 (3.4%) | 191 (2.9%) | 157 (3.6%) | 27 (2.4%) |  |  |  |
|  | SICU | 1671 (4.5%) | 59 (3.9%) | 61 (3.7%) | 85 (3.5%) | 255 (4.4%) | 433 (5.4%) | 245 (4.4%) | 306 (4.6%) | 189 (4.3%) | 38 (3.4%) |  |  |  |
| Charlson comorbidity index |  | 4.00 [2.00, 6.00] | 4.00 [2.00, 6.00] | 4.00 [3.00, 6.00] | 4.00 [3.00, 6.00] | 4.00 [2.00, 6.00] | 4.00 [2.00, 6.00] | 4.00 [3.00, 6.00] | 4.00 [2.00, 6.00] | 4.00 [2.00, 6.00] | 4.00 [2.00, 6.00] | 0.224 | 0 | 0.0 |
| APACHE IV |  | 64.00 [49.00, 82.00] | 76.00 [60.00, 100.00] | 65.00 [51.00, 81.00] | 60.00 [47.00, 75.00] | 71.00 [56.00, 92.00] | 60.00 [46.00, 75.00] | 57.00 [44.00, 72.00] | 72.00 [55.00, 93.00] | 62.00 [47.00, 79.00] | 64.00 [49.00, 81.00] | 0.846 | 3932 | 10.61 |
| SOFA |  | 7.00 [5.00, 9.00] | 8.00 [6.00, 11.00] | 6.00 [5.00, 9.00] | 6.00 [4.00, 8.00] | 7.00 [5.00, 10.00] | 6.00 [4.00, 8.00] | 6.00 [4.00, 8.00] | 7.00 [5.00, 10.00] | 6.00 [4.00, 9.00] | 7.00 [5.00, 9.00] | 0.636 | 0 | 0.0 |
| Maximum dynamic SOFA |  | 6.00 [4.00, 8.00] | 7.00 [5.00, 10.00] | 6.00 [4.00, 8.00] | 6.00 [4.00, 8.00] | 7.00 [5.00, 9.00] | 6.00 [4.00, 8.00] | 5.00 [4.00, 8.00] | 7.00 [5.00, 9.00] | 6.00 [4.00, 8.00] | 6.00 [4.00, 9.00] | 0.605 | 0 | 0.0 |
| OASIS |  | 28.00 [22.00, 36.00] | 30.00 [23.00, 39.00] | 27.00 [21.00, 35.00] | 27.00 [22.00, 34.00] | 30.00 [23.00, 38.00] | 27.00 [21.00, 35.00] | 27.00 [22.00, 34.00] | 30.00 [23.00, 39.00] | 29.00 [22.00, 36.00] | 30.00 [24.00, 38.00] | 0.33 | 0 | 0.0 |
| Septic shock | Yes | 1117 (3.0%) | 110 (7.3%) | 32 (2.0%) | 22 (0.9%) | 323 (5.6%) | 112 (1.4%) | 35 (0.6%) | 378 (5.7%) | 90 (2.0%) | 15 (1.3%) | 0.347 | 0 | 0.0 |
| AKI | Yes | 19566 (52.8%) | 1006 (66.6%) | 994 (61.1%) | 1342 (55.5%) | 3464 (59.8%) | 4012 (50.4%) | 2750 (49.0%) | 3503 (53.1%) | 1971 (44.7%) | 524 (47.1%) | 0.452 | 0 | 0.0 |
| Hypertension | Yes | 5272 (14.2%) | 149 (9.9%) | 260 (16.0%) | 389 (16.1%) | 660 (11.4%) | 1277 (16.0%) | 1007 (17.9%) | 714 (10.8%) | 648 (14.7%) | 168 (15.1%) | 0.235 | 0 | 0.0 |
| Diabetes | Yes | 12123 (32.7%) | 624 (41.3%) | 659 (40.5%) | 854 (35.3%) | 2111 (36.5%) | 2583 (32.4%) | 1939 (34.5%) | 1904 (28.8%) | 1121 (25.4%) | 328 (29.5%) | 0.342 | 0 | 0.0 |
| Renal disease | Yes | 6374 (17.2%) | 416 (27.5%) | 444 (27.3%) | 489 (20.2%) | 1090 (18.8%) | 1333 (16.7%) | 954 (17.0%) | 1004 (15.2%) | 516 (11.7%) | 128 (11.5%) | 0.413 | 0 | 0.0 |
| Congestive heart failure | Yes | 6745 (18.2%) | 302 (20.0%) | 439 (27.0%) | 723 (29.9%) | 926 (16.0%) | 1372 (17.2%) | 1462 (26.0%) | 734 (11.1%) | 576 (13.1%) | 211 (19.0%) | 0.479 | 0 | 0.0 |
| Chronic pulmonary disease | Yes | 7407 (20.0%) | 228 (15.1%) | 366 (22.5%) | 978 (40.5%) | 848 (14.6%) | 1417 (17.8%) | 1795 (32.0%) | 836 (12.7%) | 669 (15.2%) | 270 (24.3%) | 0.664 | 0 | 0.0 |
| Malignant cancer | Yes | 5675 (15.3%) | 221 (14.6%) | 274 (16.8%) | 361 (14.9%) | 932 (16.1%) | 1241 (15.6%) | 823 (14.7%) | 1039 (15.7%) | 618 (14.0%) | 166 (14.9%) | 0.078 | 0 | 0.0 |
| Chloride, mmol/L |  | 105.00 [100.00, 109.00] | 94.00 [91.00, 96.00] | 95.00 [92.00, 96.00] | 94.00 [91.00, 96.00] | 104.00 [101.00, 106.00] | 104.00 [101.00, 106.00] | 102.00 [100.00, 104.00] | 112.00 [109.00, 115.00] | 110.00 [109.00, 113.00] | 110.00 [108.00, 112.00] | 4.555 | 24 | 0.06 |
| Bicarbonate, mmol/L |  | 23.00 [20.00, 26.00] | 18.00 [16.00, 20.00] | 24.00 [23.00, 25.00] | 31.00 [28.00, 35.00] | 19.00 [16.00, 20.00] | 24.00 [23.00, 25.00] | 29.00 [28.00, 31.00] | 18.00 [16.00, 20.00] | 23.00 [22.00, 25.00] | 28.00 [27.00, 30.00] | 4.074 | 73 | 0.2 |
| CHBI |  | -0.17 [-1.31, 0.97] | -0.79 [-1.16, -0.16] | -1.89 [-2.28, -1.62] | -3.48 [-4.48, -2.80] | 0.60 [0.16, 1.12] | -0.54 [-0.96, -0.19] | -1.85 [-2.36, -1.37] | 1.98 [1.44, 2.66] | 0.55 [0.26, 0.97] | -0.50 [-0.77, -0.13] | 4.98 | 0 | 0.0 |
| Absolute CHBI |  | 1.14 [0.53, 2.01] | 0.85 [0.47, 1.28] | 1.89 [1.62, 2.28] | 3.48 [2.80, 4.48] | 0.62 [0.31, 1.12] | 0.54 [0.24, 0.96] | 1.85 [1.37, 2.36] | 1.98 [1.44, 2.66] | 0.55 [0.30, 0.97] | 0.51 [0.34, 0.92] | 3.143 | 0 | 0.0 |
| pH |  | 7.35 [7.26, 7.42] | 7.32 [7.20, 7.39] | 7.38 [7.32, 7.44] | 7.36 [7.28, 7.44] | 7.32 [7.22, 7.39] | 7.38 [7.30, 7.43] | 7.36 [7.28, 7.43] | 7.31 [7.23, 7.39] | 7.36 [7.29, 7.42] | 7.37 [7.29, 7.44] | 0.648 | 16065 | 43.37 |
| PaCO₂, mmHg |  | 40.00 [32.70, 50.90] | 33.00 [27.00, 40.20] | 40.00 [34.52, 48.00] | 57.00 [45.00, 77.00] | 34.00 [28.00, 42.00] | 40.50 [35.00, 48.80] | 51.00 [42.02, 67.00] | 33.00 [27.30, 40.40] | 40.00 [34.80, 48.00] | 47.00 [40.00, 58.00] | 1.499 | 16053 | 43.33 |
| Lactate, mmol/L |  | 2.00 [1.20, 3.50] | 3.10 [1.60, 6.10] | 2.10 [1.30, 3.30] | 1.60 [1.00, 2.50] | 2.50 [1.50, 4.70] | 1.90 [1.20, 3.00] | 1.50 [1.00, 2.40] | 2.40 [1.40, 4.40] | 1.90 [1.20, 3.10] | 1.75 [1.20, 2.70] | 0.786 | 13154 | 35.51 |
| Anion gap |  | 12.00 [9.00, 15.00] | 19.00 [15.00, 24.48] | 14.00 [11.00, 17.00] | 10.00 [7.00, 13.00] | 15.00 [12.00, 19.00] | 11.70 [9.00, 14.00] | 9.00 [7.00, 12.00] | 13.00 [10.00, 17.00] | 10.00 [7.00, 13.00] | 8.00 [6.00, 11.00] | 1.841 | 7518 | 20.29 |
| Albumin, g/dL |  | 2.80 [2.30, 3.30] | 2.80 [2.20, 3.30] | 2.90 [2.40, 3.40] | 3.10 [2.60, 3.50] | 2.70 [2.20, 3.30] | 2.90 [2.40, 3.40] | 2.90 [2.50, 3.40] | 2.60 [2.10, 3.10] | 2.80 [2.30, 3.30] | 2.70 [2.20, 3.20] | 0.551 | 9931 | 26.81 |
| Creatinine, mg/dL |  | 1.30 [0.85, 2.20] | 2.53 [1.34, 4.91] | 1.50 [0.90, 3.50] | 1.01 [0.70, 1.80] | 1.80 [1.12, 3.10] | 1.17 [0.81, 1.90] | 1.03 [0.73, 1.55] | 1.55 [1.00, 2.45] | 1.10 [0.80, 1.63] | 1.07 [0.75, 1.49] | 0.993 | 87 | 0.23 |
| BUN, mg/dL |  | 25.00 [16.00, 42.00] | 43.65 [24.00, 69.00] | 28.00 [16.00, 48.00] | 23.00 [15.00, 37.00] | 33.00 [19.00, 53.00] | 22.00 [14.00, 36.00] | 21.00 [14.00, 33.00] | 30.00 [18.00, 49.00] | 22.00 [14.00, 36.00] | 25.00 [16.00, 40.00] | 0.872 | 43 | 0.12 |
| Invasive ventilation | Yes | 18464 (49.8%) | 745 (49.3%) | 748 (45.9%) | 1469 (60.8%) | 2776 (47.9%) | 3634 (45.6%) | 3114 (55.4%) | 3197 (48.4%) | 2149 (48.8%) | 632 (56.8%) | 0.307 | 0 | 0.0 |
| Vasoactive therapy | Yes | 7754 (20.9%) | 495 (32.8%) | 306 (18.8%) | 278 (11.5%) | 1698 (29.3%) | 1335 (16.8%) | 657 (11.7%) | 1973 (29.9%) | 819 (18.6%) | 193 (17.3%) | 0.53 | 0 | 0.0 |
| Sodium bicarbonate therapy | Yes | 3425 (9.2%) | 351 (23.2%) | 79 (4.9%) | 45 (1.9%) | 1079 (18.6%) | 273 (3.4%) | 99 (1.8%) | 1302 (19.7%) | 177 (4.0%) | 20 (1.8%) | 0.687 | 0 | 0.0 |
| Fluid input, mL |  | 5210.30 [2195.76, 10754.90] | 5621.50 [2074.98, 11490.48] | 4443.55 [1828.28, 9481.50] | 4360.00 [1807.00, 9477.12] | 5369.70 [2196.51, 11024.92] | 4830.00 [2141.50, 9988.83] | 4390.41 [1911.10, 9488.35] | 6303.00 [2730.50, 12558.30] | 5550.00 [2430.00, 11006.67] | 5664.50 [2344.31, 12501.25] | 0.17 | 14997 | 40.48 |
| Urine output, mL |  | 3400.00 [1235.00, 7860.00] | 2700.00 [470.00, 7884.00] | 2980.00 [730.25, 6987.50] | 4015.00 [1550.00, 9201.25] | 3100.00 [975.00, 7400.00] | 3400.00 [1350.00, 7530.00] | 3550.00 [1400.00, 7920.00] | 3355.00 [1170.75, 7835.00] | 3525.00 [1500.00, 8470.00] | 4139.00 [1480.25, 9715.00] | 0.208 | 7027 | 18.97 |

*Note.* Continuous variables are presented as median [interquartile range] and categorical variables as n (%). Max pairwise SMD is the largest absolute pairwise standardized mean difference across the nine phenotype groups. Missing n and Missing % are calculated within the final eICU in-hospital mortality cohort (n=37,045) and describe downstream source-variable/continuous-variable missingness after analysis preprocessing.

**Supplementary Table 3.** Overall comparison between the final MIMIC-IV v3.1 and eICU v2.0 analysis cohorts.

| **Characteristic** | **Level** | **Overall** | **MIMIC-IV** | **eICU** | **Maximum pairwise SMD** | **Missing, n** | **Missing, %** |
| --- | --- | --- | --- | --- | --- | --- | --- |
| In-hospital mortality | Yes | 9957 (15.8%) | 4216 (16.3%) | 5741 (15.5%) | 0.022 | 0 | 0.0 |
| Age, years |  | 66.84 [55.04, 77.00] | 66.71 [55.83, 76.74] | 67.00 [55.00, 78.00] | 0.014 | 0 | 0.0 |
| Male sex | Yes | 34522 (54.9%) | 15286 (59.2%) | 19236 (51.9%) | 0.146 | 4 | 0.01 |
| Race/ethnicity | Asian | 1305 (2.1%) | 755 (2.9%) | 550 (1.5%) | 0.394 | 0 | 0.0 |
|  | Black | 6079 (9.7%) | 2201 (8.5%) | 3878 (10.5%) |  |  |  |
|  | Hispanic | 2744 (4.4%) | 900 (3.5%) | 1844 (5.0%) |  |  |  |
|  | Other | 1404 (2.2%) | 1114 (4.3%) | 290 (0.8%) |  |  |  |
|  | Unknown | 5810 (9.2%) | 4175 (16.2%) | 1635 (4.4%) |  |  |  |
|  | White | 45545 (72.4%) | 16697 (64.6%) | 28848 (77.9%) |  |  |  |
| Charlson comorbidity index |  | 4.00 [2.00, 6.00] | 5.00 [3.00, 7.00] | 4.00 [2.00, 6.00] | 0.225 | 0 | 0.0 |
| OASIS |  | 31.00 [24.00, 38.00] | 33.00 [28.00, 39.00] | 28.00 [22.00, 36.00] | 0.454 | 0 | 0.0 |
| Septic shock | Yes | 7568 (12.0%) | 6451 (25.0%) | 1117 (3.0%) | 0.667 | 0 | 0.0 |
| AKI | Yes | 39702 (63.1%) | 20136 (77.9%) | 19566 (52.8%) | 0.547 | 0 | 0.0 |
| Chloride, mmol/L |  | 105.00 [100.00, 109.00] | 105.00 [101.00, 109.00] | 105.00 [100.00, 109.00] | 0.015 | 36 | 0.06 |
| Bicarbonate, mmol/L |  | 23.00 [20.00, 26.00] | 22.00 [20.00, 25.00] | 23.00 [20.00, 26.00] | 0.179 | 95 | 0.15 |
| CHBI |  | -0.05 [-1.12, 0.95] | 0.08 [-0.87, 0.93] | -0.17 [-1.31, 0.97] | 0.126 | 0 | 0.0 |
| Absolute CHBI |  | 1.03 [0.48, 1.85] | 0.90 [0.42, 1.61] | 1.14 [0.53, 2.01] | 0.234 | 0 | 0.0 |
| pH |  | 7.36 [7.28, 7.42] | 7.38 [7.31, 7.43] | 7.35 [7.26, 7.42] | 0.259 | 25409 | 40.4 |
| PaCO₂, mmHg |  | 40.00 [34.00, 48.00] | 40.00 [35.00, 46.00] | 40.00 [32.70, 50.90] | 0.142 | 25404 | 40.4 |
| Lactate, mmol/L |  | 1.80 [1.20, 3.10] | 1.60 [1.10, 2.50] | 2.00 [1.20, 3.50] | 0.223 | 24237 | 38.54 |
| Anion gap |  | 13.00 [10.00, 16.00] | 14.00 [12.00, 17.00] | 12.00 [9.00, 15.00] | 0.423 | 7561 | 12.02 |
| Albumin, g/dL |  | 2.90 [2.40, 3.40] | 3.00 [2.60, 3.50] | 2.80 [2.30, 3.30] | 0.285 | 25876 | 41.15 |
| Creatinine, mg/dL |  | 1.19 [0.80, 1.95] | 1.00 [0.80, 1.60] | 1.30 [0.85, 2.20] | 0.218 | 99 | 0.16 |
| BUN, mg/dL |  | 23.00 [15.00, 39.00] | 20.00 [14.00, 34.00] | 25.00 [16.00, 42.00] | 0.196 | 55 | 0.09 |
| Invasive ventilation | Yes | 34115 (54.2%) | 15651 (60.6%) | 18464 (49.8%) | 0.217 | 0 | 0.0 |
| Vasoactive therapy | Yes | 20963 (33.3%) | 13209 (51.1%) | 7754 (20.9%) | 0.662 | 0 | 0.0 |
| Sodium bicarbonate therapy | Yes | 5655 (9.0%) | 2230 (8.6%) | 3425 (9.2%) | 0.022 | 0 | 0.0 |
| Fluid input, mL |  | 5050.50 [2480.00, 8767.80] | 4962.49 [2698.06, 7821.82] | 5210.30 [2195.76, 10754.90] | 0.136 | 17100 | 27.19 |
| Urine output, mL |  | 2035.00 [1012.00, 4300.00] | 1540.00 [920.00, 2350.00] | 3400.00 [1235.00, 7860.00] | 0.628 | 7640 | 12.15 |

*Note.* Race/ethnicity categories were harmonized across databases for this descriptive comparison: Caucasian was mapped to White, African American to Black, and Native American to Other. Continuous variables are presented as median [interquartile range] and categorical variables as n (%).

**Supplementary Table 4.** Complete-case Model 3b associations of CHBI and chloride-bicarbonate exposure representations with in-hospital mortality.

| **Cohort** | **Model** | **Exposure** | **Contrast** | **Reference** | **n** | **Events** | **OR (95% CI)** | **P** | **Overall chi-square** | **df** | **Converged** | **Overall P** |
| --- | --- | --- | --- | --- | --- | --- | --- | --- | --- | --- | --- | --- |
| eICU | Model 3b | Absolute CHBI | Per 1-SD increase | — | 21414 | 3907 | 1.057 (1.015–1.100) | 0.007 | 7.289 | 1 | Yes | 0.007 |
| eICU | Model 3b | CHBI | Per 1-SD increase | — | 21414 | 3907 | 0.973 (0.933–1.016) | 0.212 | 1.553 | 1 | Yes | 0.213 |
| eICU | Model 3b | CHBI quartiles | Q1 (lowest) | Q2 | 21414 | 3907 | 1.145 (1.015–1.291) | 0.027 | 8.594 | 3 | Yes | 0.035 |
| eICU | Model 3b | CHBI quartiles | Q3 | Q2 | 21414 | 3907 | 0.975 (0.855–1.111) | 0.699 | 8.594 | 3 | Yes | 0.035 |
| eICU | Model 3b | CHBI quartiles | Q4 (highest) | Q2 | 21414 | 3907 | 1.016 (0.902–1.145) | 0.788 | 8.594 | 3 | Yes | 0.035 |
| eICU | Model 3b | Chloride-bicarbonate phenotype | Low Cl + Low HCO₃ | Normal Cl + Normal HCO₃ | 21414 | 3907 | 1.426 (1.169–1.739) | <0.001 | 52.567 | 8 | Yes | <0.001 |
| eICU | Model 3b | Chloride-bicarbonate phenotype | Low Cl + Normal HCO₃ | Normal Cl + Normal HCO₃ | 21414 | 3907 | 1.399 (1.129–1.733) | 0.002 | 52.567 | 8 | Yes | <0.001 |
| eICU | Model 3b | Chloride-bicarbonate phenotype | Low Cl + High HCO₃ | Normal Cl + Normal HCO₃ | 21414 | 3907 | 1.166 (0.952–1.430) | 0.138 | 52.567 | 8 | Yes | <0.001 |
| eICU | Model 3b | Chloride-bicarbonate phenotype | Normal Cl + Low HCO₃ | Normal Cl + Normal HCO₃ | 21414 | 3907 | 1.207 (1.053–1.384) | 0.007 | 52.567 | 8 | Yes | <0.001 |
| eICU | Model 3b | Chloride-bicarbonate phenotype | Normal Cl + High HCO₃ | Normal Cl + Normal HCO₃ | 21414 | 3907 | 1.100 (0.940–1.288) | 0.233 | 52.567 | 8 | Yes | <0.001 |
| eICU | Model 3b | Chloride-bicarbonate phenotype | High Cl + Low HCO₃ | Normal Cl + Normal HCO₃ | 21414 | 3907 | 1.084 (0.949–1.238) | 0.233 | 52.567 | 8 | Yes | <0.001 |
| eICU | Model 3b | Chloride-bicarbonate phenotype | High Cl + Normal HCO₃ | Normal Cl + Normal HCO₃ | 21414 | 3907 | 0.758 (0.641–0.895) | 0.001 | 52.567 | 8 | Yes | <0.001 |
| eICU | Model 3b | Chloride-bicarbonate phenotype | High Cl + High HCO₃ | Normal Cl + Normal HCO₃ | 21414 | 3907 | 0.903 (0.693–1.176) | 0.448 | 52.567 | 8 | Yes | <0.001 |
| MIMIC-IV | Model 3b | Absolute CHBI | Per 1-SD increase | — | 13168 | 2370 | 1.007 (0.957–1.059) | 0.785 | 0.075 | 1 | Yes | 0.785 |
| MIMIC-IV | Model 3b | CHBI | Per 1-SD increase | — | 13168 | 2370 | 0.940 (0.892–0.990) | 0.019 | 5.498 | 1 | Yes | 0.019 |
| MIMIC-IV | Model 3b | CHBI quartiles | Q1 (lowest) | Q2 | 13168 | 2370 | 1.187 (1.015–1.388) | 0.032 | 10.356 | 3 | Yes | 0.016 |
| MIMIC-IV | Model 3b | CHBI quartiles | Q3 | Q2 | 13168 | 2370 | 0.929 (0.788–1.096) | 0.382 | 10.356 | 3 | Yes | 0.016 |
| MIMIC-IV | Model 3b | CHBI quartiles | Q4 (highest) | Q2 | 13168 | 2370 | 0.977 (0.836–1.142) | 0.769 | 10.356 | 3 | Yes | 0.016 |
| MIMIC-IV | Model 3b | Chloride-bicarbonate phenotype | Low Cl + Low HCO₃ | Normal Cl + Normal HCO₃ | 13168 | 2370 | 1.136 (0.885–1.460) | 0.317 | 18.587 | 8 | Yes | 0.017 |
| MIMIC-IV | Model 3b | Chloride-bicarbonate phenotype | Low Cl + Normal HCO₃ | Normal Cl + Normal HCO₃ | 13168 | 2370 | 1.519 (1.159–1.990) | 0.002 | 18.587 | 8 | Yes | 0.017 |
| MIMIC-IV | Model 3b | Chloride-bicarbonate phenotype | Low Cl + High HCO₃ | Normal Cl + Normal HCO₃ | 13168 | 2370 | 0.989 (0.742–1.319) | 0.940 | 18.587 | 8 | Yes | 0.017 |
| MIMIC-IV | Model 3b | Chloride-bicarbonate phenotype | Normal Cl + Low HCO₃ | Normal Cl + Normal HCO₃ | 13168 | 2370 | 0.963 (0.813–1.141) | 0.666 | 18.587 | 8 | Yes | 0.017 |
| MIMIC-IV | Model 3b | Chloride-bicarbonate phenotype | Normal Cl + High HCO₃ | Normal Cl + Normal HCO₃ | 13168 | 2370 | 0.925 (0.736–1.162) | 0.502 | 18.587 | 8 | Yes | 0.017 |
| MIMIC-IV | Model 3b | Chloride-bicarbonate phenotype | High Cl + Low HCO₃ | Normal Cl + Normal HCO₃ | 13168 | 2370 | 0.920 (0.770–1.099) | 0.356 | 18.587 | 8 | Yes | 0.017 |
| MIMIC-IV | Model 3b | Chloride-bicarbonate phenotype | High Cl + Normal HCO₃ | Normal Cl + Normal HCO₃ | 13168 | 2370 | 0.841 (0.673–1.049) | 0.125 | 18.587 | 8 | Yes | 0.017 |
| MIMIC-IV | Model 3b | Chloride-bicarbonate phenotype | High Cl + High HCO₃ | Normal Cl + Normal HCO₃ | 13168 | 2370 | 1.189 (0.773–1.827) | 0.430 | 18.587 | 8 | Yes | 0.017 |

*Note.* Continuous CHBI terms are reported per 1-SD increase. Q2 is the reference category for CHBI quartiles. Normal chloride plus normal bicarbonate is the reference phenotype. Overall P values are from likelihood-ratio tests.

**Supplementary Table 5.** Extended complete-case acid-base adjustment models.

| **Cohort** | **Model** | **Exposure** | **Contrast** | **Reference** | **n** | **Events** | **OR (95% CI)** | **P** | **Overall chi-square** | **df** | **Converged** | **Overall P** |
| --- | --- | --- | --- | --- | --- | --- | --- | --- | --- | --- | --- | --- |
| eICU | Model 4b-Full | Absolute CHBI | Per 1-SD increase | — | 8929 | 2126 | 1.110 (1.050–1.174) | <0.001 | 13.337 | 1 | Yes | <0.001 |
| eICU | Model 4b-Full | CHBI | Per 1-SD increase | — | 8929 | 2126 | 0.932 (0.865–1.004) | 0.064 | 3.433 | 1 | Yes | 0.064 |
| eICU | Model 4b-Full | CHBI quartiles | Q1 (lowest) | Q2 | 8929 | 2126 | 1.150 (0.960–1.377) | 0.128 | 12.918 | 3 | Yes | 0.005 |
| eICU | Model 4b-Full | CHBI quartiles | Q3 | Q2 | 8929 | 2126 | 0.817 (0.675–0.988) | 0.038 | 12.918 | 3 | Yes | 0.005 |
| eICU | Model 4b-Full | CHBI quartiles | Q4 (highest) | Q2 | 8929 | 2126 | 0.915 (0.768–1.091) | 0.324 | 12.918 | 3 | Yes | 0.005 |
| eICU | Model 4b-Full | Chloride-bicarbonate phenotype | Low Cl + Low HCO₃ | Normal Cl + Normal HCO₃ | 8929 | 2126 | 1.254 (0.929–1.693) | 0.140 | 26.181 | 8 | Yes | <0.001 |
| eICU | Model 4b-Full | Chloride-bicarbonate phenotype | Low Cl + Normal HCO₃ | Normal Cl + Normal HCO₃ | 8929 | 2126 | 1.178 (0.841–1.649) | 0.342 | 26.181 | 8 | Yes | <0.001 |
| eICU | Model 4b-Full | Chloride-bicarbonate phenotype | Low Cl + High HCO₃ | Normal Cl + Normal HCO₃ | 8929 | 2126 | 1.334 (0.984–1.809) | 0.063 | 26.181 | 8 | Yes | <0.001 |
| eICU | Model 4b-Full | Chloride-bicarbonate phenotype | Normal Cl + Low HCO₃ | Normal Cl + Normal HCO₃ | 8929 | 2126 | 1.009 (0.821–1.239) | 0.935 | 26.181 | 8 | Yes | <0.001 |
| eICU | Model 4b-Full | Chloride-bicarbonate phenotype | Normal Cl + High HCO₃ | Normal Cl + Normal HCO₃ | 8929 | 2126 | 1.102 (0.874–1.389) | 0.412 | 26.181 | 8 | Yes | <0.001 |
| eICU | Model 4b-Full | Chloride-bicarbonate phenotype | High Cl + Low HCO₃ | Normal Cl + Normal HCO₃ | 8929 | 2126 | 0.963 (0.790–1.174) | 0.711 | 26.181 | 8 | Yes | <0.001 |
| eICU | Model 4b-Full | Chloride-bicarbonate phenotype | High Cl + Normal HCO₃ | Normal Cl + Normal HCO₃ | 8929 | 2126 | 0.703 (0.556–0.890) | 0.003 | 26.181 | 8 | Yes | <0.001 |
| eICU | Model 4b-Full | Chloride-bicarbonate phenotype | High Cl + High HCO₃ | Normal Cl + Normal HCO₃ | 8929 | 2126 | 0.746 (0.514–1.083) | 0.124 | 26.181 | 8 | Yes | <0.001 |
| eICU | Model 4b-NoAG | Absolute CHBI | Per 1-SD increase | — | 10836 | 2594 | 1.083 (1.029–1.140) | 0.002 | 9.355 | 1 | Yes | 0.002 |
| eICU | Model 4b-NoAG | CHBI | Per 1-SD increase | — | 10836 | 2594 | 0.903 (0.844–0.966) | 0.003 | 8.732 | 1 | Yes | 0.003 |
| eICU | Model 4b-NoAG | CHBI quartiles | Q1 (lowest) | Q2 | 10836 | 2594 | 1.132 (0.964–1.329) | 0.131 | 20.204 | 3 | Yes | <0.001 |
| eICU | Model 4b-NoAG | CHBI quartiles | Q3 | Q2 | 10836 | 2594 | 0.784 (0.660–0.931) | 0.005 | 20.204 | 3 | Yes | <0.001 |
| eICU | Model 4b-NoAG | CHBI quartiles | Q4 (highest) | Q2 | 10836 | 2594 | 0.851 (0.726–0.998) | 0.047 | 20.204 | 3 | Yes | <0.001 |
| eICU | Model 4b-NoAG | Chloride-bicarbonate phenotype | Low Cl + Low HCO₃ | Normal Cl + Normal HCO₃ | 10836 | 2594 | 1.261 (0.975–1.631) | 0.077 | 37.722 | 8 | Yes | <0.001 |
| eICU | Model 4b-NoAG | Chloride-bicarbonate phenotype | Low Cl + Normal HCO₃ | Normal Cl + Normal HCO₃ | 10836 | 2594 | 1.236 (0.931–1.640) | 0.142 | 37.722 | 8 | Yes | <0.001 |
| eICU | Model 4b-NoAG | Chloride-bicarbonate phenotype | Low Cl + High HCO₃ | Normal Cl + Normal HCO₃ | 10836 | 2594 | 1.221 (0.931–1.601) | 0.149 | 37.722 | 8 | Yes | <0.001 |
| eICU | Model 4b-NoAG | Chloride-bicarbonate phenotype | Normal Cl + Low HCO₃ | Normal Cl + Normal HCO₃ | 10836 | 2594 | 0.974 (0.811–1.171) | 0.783 | 37.722 | 8 | Yes | <0.001 |
| eICU | Model 4b-NoAG | Chloride-bicarbonate phenotype | Normal Cl + High HCO₃ | Normal Cl + Normal HCO₃ | 10836 | 2594 | 0.998 (0.808–1.233) | 0.987 | 37.722 | 8 | Yes | <0.001 |
| eICU | Model 4b-NoAG | Chloride-bicarbonate phenotype | High Cl + Low HCO₃ | Normal Cl + Normal HCO₃ | 10836 | 2594 | 0.893 (0.745–1.070) | 0.219 | 37.722 | 8 | Yes | <0.001 |
| eICU | Model 4b-NoAG | Chloride-bicarbonate phenotype | High Cl + Normal HCO₃ | Normal Cl + Normal HCO₃ | 10836 | 2594 | 0.658 (0.531–0.816) | <0.001 | 37.722 | 8 | Yes | <0.001 |
| eICU | Model 4b-NoAG | Chloride-bicarbonate phenotype | High Cl + High HCO₃ | Normal Cl + Normal HCO₃ | 10836 | 2594 | 0.713 (0.508–1.000) | 0.050 | 37.722 | 8 | Yes | <0.001 |
| eICU | Model 4b-pH/PaCO₂ | Absolute CHBI | Per 1-SD increase | — | 13243 | 3072 | 1.065 (1.018–1.115) | 0.007 | 7.292 | 1 | Yes | 0.007 |
| eICU | Model 4b-pH/PaCO₂ | CHBI | Per 1-SD increase | — | 13243 | 3072 | 0.936 (0.881–0.994) | 0.031 | 4.640 | 1 | Yes | 0.031 |
| eICU | Model 4b-pH/PaCO₂ | CHBI quartiles | Q1 (lowest) | Q2 | 13243 | 3072 | 1.135 (0.982–1.312) | 0.086 | 10.359 | 3 | Yes | 0.016 |
| eICU | Model 4b-pH/PaCO₂ | CHBI quartiles | Q3 | Q2 | 13243 | 3072 | 0.904 (0.776–1.054) | 0.199 | 10.359 | 3 | Yes | 0.016 |
| eICU | Model 4b-pH/PaCO₂ | CHBI quartiles | Q4 (highest) | Q2 | 13243 | 3072 | 0.920 (0.797–1.062) | 0.255 | 10.359 | 3 | Yes | 0.016 |
| eICU | Model 4b-pH/PaCO₂ | Chloride-bicarbonate phenotype | Low Cl + Low HCO₃ | Normal Cl + Normal HCO₃ | 13243 | 3072 | 1.378 (1.091–1.740) | 0.007 | 35.773 | 8 | Yes | <0.001 |
| eICU | Model 4b-pH/PaCO₂ | Chloride-bicarbonate phenotype | Low Cl + Normal HCO₃ | Normal Cl + Normal HCO₃ | 13243 | 3072 | 1.227 (0.948–1.589) | 0.120 | 35.773 | 8 | Yes | <0.001 |
| eICU | Model 4b-pH/PaCO₂ | Chloride-bicarbonate phenotype | Low Cl + High HCO₃ | Normal Cl + Normal HCO₃ | 13243 | 3072 | 1.225 (0.962–1.560) | 0.100 | 35.773 | 8 | Yes | <0.001 |
| eICU | Model 4b-pH/PaCO₂ | Chloride-bicarbonate phenotype | Normal Cl + Low HCO₃ | Normal Cl + Normal HCO₃ | 13243 | 3072 | 1.120 (0.949–1.321) | 0.180 | 35.773 | 8 | Yes | <0.001 |
| eICU | Model 4b-pH/PaCO₂ | Chloride-bicarbonate phenotype | Normal Cl + High HCO₃ | Normal Cl + Normal HCO₃ | 13243 | 3072 | 1.100 (0.912–1.327) | 0.320 | 35.773 | 8 | Yes | <0.001 |
| eICU | Model 4b-pH/PaCO₂ | Chloride-bicarbonate phenotype | High Cl + Low HCO₃ | Normal Cl + Normal HCO₃ | 13243 | 3072 | 1.000 (0.851–1.176) | 0.997 | 35.773 | 8 | Yes | <0.001 |
| eICU | Model 4b-pH/PaCO₂ | Chloride-bicarbonate phenotype | High Cl + Normal HCO₃ | Normal Cl + Normal HCO₃ | 13243 | 3072 | 0.742 (0.611–0.900) | 0.002 | 35.773 | 8 | Yes | <0.001 |
| eICU | Model 4b-pH/PaCO₂ | Chloride-bicarbonate phenotype | High Cl + High HCO₃ | Normal Cl + Normal HCO₃ | 13243 | 3072 | 0.841 (0.619–1.142) | 0.267 | 35.773 | 8 | Yes | <0.001 |
| MIMIC-IV | Model 4b-Full | Absolute CHBI | Per 1-SD increase | — | 4793 | 1298 | 0.986 (0.918–1.059) | 0.706 | 0.143 | 1 | Yes | 0.706 |
| MIMIC-IV | Model 4b-Full | CHBI | Per 1-SD increase | — | 4793 | 1298 | 0.984 (0.892–1.085) | 0.742 | 0.108 | 1 | Yes | 0.742 |
| MIMIC-IV | Model 4b-Full | CHBI quartiles | Q1 (lowest) | Q2 | 4793 | 1298 | 1.158 (0.918–1.461) | 0.215 | 2.203 | 3 | Yes | 0.531 |
| MIMIC-IV | Model 4b-Full | CHBI quartiles | Q3 | Q2 | 4793 | 1298 | 1.020 (0.803–1.295) | 0.873 | 2.203 | 3 | Yes | 0.531 |
| MIMIC-IV | Model 4b-Full | CHBI quartiles | Q4 (highest) | Q2 | 4793 | 1298 | 1.107 (0.874–1.401) | 0.400 | 2.203 | 3 | Yes | 0.531 |
| MIMIC-IV | Model 4b-Full | Chloride-bicarbonate phenotype | Low Cl + Low HCO₃ | Normal Cl + Normal HCO₃ | 4793 | 1298 | 1.466 (1.044–2.060) | 0.027 | 13.363 | 8 | Yes | 0.100 |
| MIMIC-IV | Model 4b-Full | Chloride-bicarbonate phenotype | Low Cl + Normal HCO₃ | Normal Cl + Normal HCO₃ | 4793 | 1298 | 1.667 (1.149–2.419) | 0.007 | 13.363 | 8 | Yes | 0.100 |
| MIMIC-IV | Model 4b-Full | Chloride-bicarbonate phenotype | Low Cl + High HCO₃ | Normal Cl + Normal HCO₃ | 4793 | 1298 | 0.917 (0.590–1.426) | 0.702 | 13.363 | 8 | Yes | 0.100 |
| MIMIC-IV | Model 4b-Full | Chloride-bicarbonate phenotype | Normal Cl + Low HCO₃ | Normal Cl + Normal HCO₃ | 4793 | 1298 | 1.284 (0.993–1.660) | 0.056 | 13.363 | 8 | Yes | 0.100 |
| MIMIC-IV | Model 4b-Full | Chloride-bicarbonate phenotype | Normal Cl + High HCO₃ | Normal Cl + Normal HCO₃ | 4793 | 1298 | 0.931 (0.657–1.321) | 0.690 | 13.363 | 8 | Yes | 0.100 |
| MIMIC-IV | Model 4b-Full | Chloride-bicarbonate phenotype | High Cl + Low HCO₃ | Normal Cl + Normal HCO₃ | 4793 | 1298 | 1.265 (0.958–1.669) | 0.097 | 13.363 | 8 | Yes | 0.100 |
| MIMIC-IV | Model 4b-Full | Chloride-bicarbonate phenotype | High Cl + Normal HCO₃ | Normal Cl + Normal HCO₃ | 4793 | 1298 | 1.055 (0.746–1.493) | 0.761 | 13.363 | 8 | Yes | 0.100 |
| MIMIC-IV | Model 4b-Full | Chloride-bicarbonate phenotype | High Cl + High HCO₃ | Normal Cl + Normal HCO₃ | 4793 | 1298 | 1.356 (0.706–2.604) | 0.360 | 13.363 | 8 | Yes | 0.100 |
| MIMIC-IV | Model 4b-NoAG | Absolute CHBI | Per 1-SD increase | — | 4793 | 1298 | 0.985 (0.918–1.058) | 0.684 | 0.166 | 1 | Yes | 0.683 |
| MIMIC-IV | Model 4b-NoAG | CHBI | Per 1-SD increase | — | 4793 | 1298 | 0.983 (0.891–1.084) | 0.726 | 0.123 | 1 | Yes | 0.726 |
| MIMIC-IV | Model 4b-NoAG | CHBI quartiles | Q1 (lowest) | Q2 | 4793 | 1298 | 1.157 (0.917–1.460) | 0.218 | 2.154 | 3 | Yes | 0.541 |
| MIMIC-IV | Model 4b-NoAG | CHBI quartiles | Q3 | Q2 | 4793 | 1298 | 1.019 (0.802–1.294) | 0.878 | 2.154 | 3 | Yes | 0.541 |
| MIMIC-IV | Model 4b-NoAG | CHBI quartiles | Q4 (highest) | Q2 | 4793 | 1298 | 1.103 (0.871–1.397) | 0.414 | 2.154 | 3 | Yes | 0.541 |
| MIMIC-IV | Model 4b-NoAG | Chloride-bicarbonate phenotype | Low Cl + Low HCO₃ | Normal Cl + Normal HCO₃ | 4793 | 1298 | 1.480 (1.060–2.067) | 0.021 | 14.030 | 8 | Yes | 0.081 |
| MIMIC-IV | Model 4b-NoAG | Chloride-bicarbonate phenotype | Low Cl + Normal HCO₃ | Normal Cl + Normal HCO₃ | 4793 | 1298 | 1.673 (1.153–2.426) | 0.007 | 14.030 | 8 | Yes | 0.081 |
| MIMIC-IV | Model 4b-NoAG | Chloride-bicarbonate phenotype | Low Cl + High HCO₃ | Normal Cl + Normal HCO₃ | 4793 | 1298 | 0.918 (0.591–1.427) | 0.704 | 14.030 | 8 | Yes | 0.081 |
| MIMIC-IV | Model 4b-NoAG | Chloride-bicarbonate phenotype | Normal Cl + Low HCO₃ | Normal Cl + Normal HCO₃ | 4793 | 1298 | 1.290 (1.000–1.665) | 0.050 | 14.030 | 8 | Yes | 0.081 |
| MIMIC-IV | Model 4b-NoAG | Chloride-bicarbonate phenotype | Normal Cl + High HCO₃ | Normal Cl + Normal HCO₃ | 4793 | 1298 | 0.928 (0.655–1.316) | 0.676 | 14.030 | 8 | Yes | 0.081 |
| MIMIC-IV | Model 4b-NoAG | Chloride-bicarbonate phenotype | High Cl + Low HCO₃ | Normal Cl + Normal HCO₃ | 4793 | 1298 | 1.264 (0.958–1.668) | 0.098 | 14.030 | 8 | Yes | 0.081 |
| MIMIC-IV | Model 4b-NoAG | Chloride-bicarbonate phenotype | High Cl + Normal HCO₃ | Normal Cl + Normal HCO₃ | 4793 | 1298 | 1.052 (0.744–1.486) | 0.776 | 14.030 | 8 | Yes | 0.081 |
| MIMIC-IV | Model 4b-NoAG | Chloride-bicarbonate phenotype | High Cl + High HCO₃ | Normal Cl + Normal HCO₃ | 4793 | 1298 | 1.346 (0.702–2.581) | 0.371 | 14.030 | 8 | Yes | 0.081 |
| MIMIC-IV | Model 4b-pH/PaCO₂ | Absolute CHBI | Per 1-SD increase | — | 13155 | 2364 | 0.999 (0.949–1.052) | 0.968 | 0.002 | 1 | Yes | 0.968 |
| MIMIC-IV | Model 4b-pH/PaCO₂ | CHBI | Per 1-SD increase | — | 13155 | 2364 | 0.994 (0.925–1.068) | 0.862 | 0.030 | 1 | Yes | 0.862 |
| MIMIC-IV | Model 4b-pH/PaCO₂ | CHBI quartiles | Q1 (lowest) | Q2 | 13155 | 2364 | 1.125 (0.952–1.328) | 0.166 | 4.290 | 3 | Yes | 0.232 |
| MIMIC-IV | Model 4b-pH/PaCO₂ | CHBI quartiles | Q3 | Q2 | 13155 | 2364 | 0.948 (0.803–1.119) | 0.525 | 4.290 | 3 | Yes | 0.232 |
| MIMIC-IV | Model 4b-pH/PaCO₂ | CHBI quartiles | Q4 (highest) | Q2 | 13155 | 2364 | 1.044 (0.885–1.232) | 0.606 | 4.290 | 3 | Yes | 0.232 |
| MIMIC-IV | Model 4b-pH/PaCO₂ | Chloride-bicarbonate phenotype | Low Cl + Low HCO₃ | Normal Cl + Normal HCO₃ | 13155 | 2364 | 1.184 (0.919–1.525) | 0.190 | 16.790 | 8 | Yes | 0.032 |
| MIMIC-IV | Model 4b-pH/PaCO₂ | Chloride-bicarbonate phenotype | Low Cl + Normal HCO₃ | Normal Cl + Normal HCO₃ | 13155 | 2364 | 1.446 (1.101–1.899) | 0.008 | 16.790 | 8 | Yes | 0.032 |
| MIMIC-IV | Model 4b-pH/PaCO₂ | Chloride-bicarbonate phenotype | Low Cl + High HCO₃ | Normal Cl + Normal HCO₃ | 13155 | 2364 | 0.855 (0.627–1.165) | 0.320 | 16.790 | 8 | Yes | 0.032 |
| MIMIC-IV | Model 4b-pH/PaCO₂ | Chloride-bicarbonate phenotype | Normal Cl + Low HCO₃ | Normal Cl + Normal HCO₃ | 13155 | 2364 | 1.049 (0.879–1.251) | 0.598 | 16.790 | 8 | Yes | 0.032 |
| MIMIC-IV | Model 4b-pH/PaCO₂ | Chloride-bicarbonate phenotype | Normal Cl + High HCO₃ | Normal Cl + Normal HCO₃ | 13155 | 2364 | 0.847 (0.667–1.077) | 0.176 | 16.790 | 8 | Yes | 0.032 |
| MIMIC-IV | Model 4b-pH/PaCO₂ | Chloride-bicarbonate phenotype | High Cl + Low HCO₃ | Normal Cl + Normal HCO₃ | 13155 | 2364 | 1.037 (0.858–1.253) | 0.710 | 16.790 | 8 | Yes | 0.032 |
| MIMIC-IV | Model 4b-pH/PaCO₂ | Chloride-bicarbonate phenotype | High Cl + Normal HCO₃ | Normal Cl + Normal HCO₃ | 13155 | 2364 | 0.861 (0.690–1.076) | 0.188 | 16.790 | 8 | Yes | 0.032 |
| MIMIC-IV | Model 4b-pH/PaCO₂ | Chloride-bicarbonate phenotype | High Cl + High HCO₃ | Normal Cl + Normal HCO₃ | 13155 | 2364 | 1.143 (0.742–1.763) | 0.544 | 16.790 | 8 | Yes | 0.032 |

*Note.* Models were refitted using stable binomial generalized linear models, and all base and full models converged. Model 4b-Full additionally includes pH, PaCO₂, anion gap, and albumin; Model 4b-NoAG excludes anion gap; Model 4b-pH/PaCO₂ adds pH and PaCO₂ to Model 3b.

**Supplementary Table 6.** Predictive mean matching multiple-imputation analysis.

| **Cohort** | **Model** | **Contrast** | **Reference** | **m** | **n min** | **n max** | **Events min** | **Events max** | **OR (95% CI)** | **P** | **Global Wald chi-square** | **df** | **Global P** | **All converged** |
| --- | --- | --- | --- | --- | --- | --- | --- | --- | --- | --- | --- | --- | --- | --- |
| MIMIC-IV | Model 3b | Low Cl + Low HCO₃ | Normal Cl + Normal HCO₃ | 20 | 25842 | 25842 | 4216 | 4216 | 1.276 (1.066–1.527) | 0.008 | 36.420 | 8 | <0.001 | Yes |
| MIMIC-IV | Model 3b | Low Cl + Normal HCO₃ | Normal Cl + Normal HCO₃ | 20 | 25842 | 25842 | 4216 | 4216 | 1.487 (1.241–1.782) | <0.001 | 36.420 | 8 | <0.001 | Yes |
| MIMIC-IV | Model 3b | Low Cl + High HCO₃ | Normal Cl + Normal HCO₃ | 20 | 25842 | 25842 | 4216 | 4216 | 1.205 (0.992–1.464) | 0.060 | 36.420 | 8 | <0.001 | Yes |
| MIMIC-IV | Model 3b | Normal Cl + Low HCO₃ | Normal Cl + Normal HCO₃ | 20 | 25842 | 25842 | 4216 | 4216 | 1.104 (0.977–1.248) | 0.113 | 36.420 | 8 | <0.001 | Yes |
| MIMIC-IV | Model 3b | Normal Cl + High HCO₃ | Normal Cl + Normal HCO₃ | 20 | 25842 | 25842 | 4216 | 4216 | 0.899 (0.762–1.061) | 0.209 | 36.420 | 8 | <0.001 | Yes |
| MIMIC-IV | Model 3b | High Cl + Low HCO₃ | Normal Cl + Normal HCO₃ | 20 | 25842 | 25842 | 4216 | 4216 | 0.987 (0.865–1.126) | 0.841 | 36.420 | 8 | <0.001 | Yes |
| MIMIC-IV | Model 3b | High Cl + Normal HCO₃ | Normal Cl + Normal HCO₃ | 20 | 25842 | 25842 | 4216 | 4216 | 0.918 (0.781–1.080) | 0.301 | 36.420 | 8 | <0.001 | Yes |
| MIMIC-IV | Model 3b | High Cl + High HCO₃ | Normal Cl + Normal HCO₃ | 20 | 25842 | 25842 | 4216 | 4216 | 1.032 (0.734–1.451) | 0.858 | 36.420 | 8 | <0.001 | Yes |
| MIMIC-IV | Model 4b-NoAG | Low Cl + Low HCO₃ | Normal Cl + Normal HCO₃ | 20 | 25842 | 25842 | 4216 | 4216 | 1.320 (1.099–1.586) | 0.003 | 36.787 | 8 | <0.001 | Yes |
| MIMIC-IV | Model 4b-NoAG | Low Cl + Normal HCO₃ | Normal Cl + Normal HCO₃ | 20 | 25842 | 25842 | 4216 | 4216 | 1.432 (1.193–1.718) | <0.001 | 36.787 | 8 | <0.001 | Yes |
| MIMIC-IV | Model 4b-NoAG | Low Cl + High HCO₃ | Normal Cl + Normal HCO₃ | 20 | 25842 | 25842 | 4216 | 4216 | 1.092 (0.881–1.355) | 0.421 | 36.787 | 8 | <0.001 | Yes |
| MIMIC-IV | Model 4b-NoAG | Normal Cl + Low HCO₃ | Normal Cl + Normal HCO₃ | 20 | 25842 | 25842 | 4216 | 4216 | 1.174 (1.030–1.338) | 0.016 | 36.787 | 8 | <0.001 | Yes |
| MIMIC-IV | Model 4b-NoAG | Normal Cl + High HCO₃ | Normal Cl + Normal HCO₃ | 20 | 25842 | 25842 | 4216 | 4216 | 0.844 (0.707–1.007) | 0.059 | 36.787 | 8 | <0.001 | Yes |
| MIMIC-IV | Model 4b-NoAG | High Cl + Low HCO₃ | Normal Cl + Normal HCO₃ | 20 | 25842 | 25842 | 4216 | 4216 | 1.049 (0.906–1.214) | 0.524 | 36.787 | 8 | <0.001 | Yes |
| MIMIC-IV | Model 4b-NoAG | High Cl + Normal HCO₃ | Normal Cl + Normal HCO₃ | 20 | 25842 | 25842 | 4216 | 4216 | 0.915 (0.777–1.078) | 0.288 | 36.787 | 8 | <0.001 | Yes |
| MIMIC-IV | Model 4b-NoAG | High Cl + High HCO₃ | Normal Cl + Normal HCO₃ | 20 | 25842 | 25842 | 4216 | 4216 | 0.963 (0.683–1.359) | 0.831 | 36.787 | 8 | <0.001 | Yes |
| eICU | Model 3b | Low Cl + Low HCO₃ | Normal Cl + Normal HCO₃ | 20 | 37041 | 37041 | 5741 | 5741 | 1.507 (1.277–1.779) | <0.001 | 77.709 | 8 | <0.001 | Yes |
| eICU | Model 3b | Low Cl + Normal HCO₃ | Normal Cl + Normal HCO₃ | 20 | 37041 | 37041 | 5741 | 5741 | 1.297 (1.094–1.537) | 0.003 | 77.709 | 8 | <0.001 | Yes |
| eICU | Model 3b | Low Cl + High HCO₃ | Normal Cl + Normal HCO₃ | 20 | 37041 | 37041 | 5741 | 5741 | 1.283 (1.107–1.487) | <0.001 | 77.709 | 8 | <0.001 | Yes |
| eICU | Model 3b | Normal Cl + Low HCO₃ | Normal Cl + Normal HCO₃ | 20 | 37041 | 37041 | 5741 | 5741 | 1.259 (1.127–1.406) | <0.001 | 77.709 | 8 | <0.001 | Yes |
| eICU | Model 3b | Normal Cl + High HCO₃ | Normal Cl + Normal HCO₃ | 20 | 37041 | 37041 | 5741 | 5741 | 1.044 (0.927–1.175) | 0.479 | 77.709 | 8 | <0.001 | Yes |
| eICU | Model 3b | High Cl + Low HCO₃ | Normal Cl + Normal HCO₃ | 20 | 37041 | 37041 | 5741 | 5741 | 1.146 (1.029–1.275) | 0.013 | 77.709 | 8 | <0.001 | Yes |
| eICU | Model 3b | High Cl + Normal HCO₃ | Normal Cl + Normal HCO₃ | 20 | 37041 | 37041 | 5741 | 5741 | 0.813 (0.715–0.924) | 0.002 | 77.709 | 8 | <0.001 | Yes |
| eICU | Model 3b | High Cl + High HCO₃ | Normal Cl + Normal HCO₃ | 20 | 37041 | 37041 | 5741 | 5741 | 0.921 (0.751–1.131) | 0.434 | 77.709 | 8 | <0.001 | Yes |
| eICU | Model 4b-NoAG | Low Cl + Low HCO₃ | Normal Cl + Normal HCO₃ | 20 | 37041 | 37041 | 5741 | 5741 | 1.499 (1.265–1.776) | <0.001 | 88.802 | 8 | <0.001 | Yes |
| eICU | Model 4b-NoAG | Low Cl + Normal HCO₃ | Normal Cl + Normal HCO₃ | 20 | 37041 | 37041 | 5741 | 5741 | 1.344 (1.131–1.596) | <0.001 | 88.802 | 8 | <0.001 | Yes |
| eICU | Model 4b-NoAG | Low Cl + High HCO₃ | Normal Cl + Normal HCO₃ | 20 | 37041 | 37041 | 5741 | 5741 | 1.344 (1.145–1.579) | <0.001 | 88.802 | 8 | <0.001 | Yes |
| eICU | Model 4b-NoAG | Normal Cl + Low HCO₃ | Normal Cl + Normal HCO₃ | 20 | 37041 | 37041 | 5741 | 5741 | 1.203 (1.071–1.350) | 0.002 | 88.802 | 8 | <0.001 | Yes |
| eICU | Model 4b-NoAG | Normal Cl + High HCO₃ | Normal Cl + Normal HCO₃ | 20 | 37041 | 37041 | 5741 | 5741 | 1.025 (0.904–1.162) | 0.702 | 88.802 | 8 | <0.001 | Yes |
| eICU | Model 4b-NoAG | High Cl + Low HCO₃ | Normal Cl + Normal HCO₃ | 20 | 37041 | 37041 | 5741 | 5741 | 1.055 (0.940–1.184) | 0.364 | 88.802 | 8 | <0.001 | Yes |
| eICU | Model 4b-NoAG | High Cl + Normal HCO₃ | Normal Cl + Normal HCO₃ | 20 | 37041 | 37041 | 5741 | 5741 | 0.779 (0.684–0.888) | <0.001 | 88.802 | 8 | <0.001 | Yes |
| eICU | Model 4b-NoAG | High Cl + High HCO₃ | Normal Cl + Normal HCO₃ | 20 | 37041 | 37041 | 5741 | 5741 | 0.848 (0.689–1.044) | 0.120 | 88.802 | 8 | <0.001 | Yes |

*Note.* Estimates were pooled across 20 predictive mean matching imputed datasets using Rubin’s rules. The phenotype reference group is normal chloride plus normal bicarbonate. Global P values are pooled Wald tests. All converged indicates convergence in every imputed dataset.

**Supplementary Table 7A.** Exposure-definition and patient-level sensitivity analyses: overall phenotype tests.

| **Cohort** | **Analysis** | **Subset description** | **n** | **Events** | **LRT chi-square** | **df** | **Overall P** | **Converged** |
| --- | --- | --- | --- | --- | --- | --- | --- | --- |
| MIMIC-IV | Main analysis | Full paired analysis set | 13168 | 2370 | 18.587 | 8 | 0.017 | Yes |
| MIMIC-IV | Exact-time pairs | Exact-time Cl–HCO₃ pairs only | 13168 | 2370 | 18.587 | 8 | 0.017 | Yes |
| MIMIC-IV | Pairing interval ≤1 h | Chloride–bicarbonate measurement interval ≤1 h | 13168 | 2370 | 18.587 | 8 | 0.017 | Yes |
| MIMIC-IV | Pairing interval ≤3 h | Chloride–bicarbonate measurement interval ≤3 h | 13168 | 2370 | 18.587 | 8 | 0.017 | Yes |
| MIMIC-IV | Exposure window 0–6 h | Paired measurements obtained within ICU hours 0–6 | 11321 | 2034 | 14.425 | 8 | 0.071 | Yes |
| MIMIC-IV | Exposure window 0–12 h | Paired measurements obtained within ICU hours 0–12 | 12775 | 2289 | 16.441 | 8 | 0.036 | Yes |
| eICU | Main analysis | Full paired analysis set | 21414 | 3907 | 52.567 | 8 | <0.001 | Yes |
| eICU | Exact-time pairs | Exact-time Cl–HCO₃ pairs only | 21246 | 3858 | 52.454 | 8 | <0.001 | Yes |
| eICU | Pairing interval ≤1 h | Chloride–bicarbonate measurement interval ≤1 h | 21295 | 3875 | 52.150 | 8 | <0.001 | Yes |
| eICU | Pairing interval ≤3 h | Chloride–bicarbonate measurement interval ≤3 h | 21355 | 3892 | 52.427 | 8 | <0.001 | Yes |
| eICU | Exposure window 0–6 h | Paired measurements obtained within ICU hours 0–6 | 11980 | 2414 | 19.166 | 8 | 0.014 | Yes |
| eICU | Exposure window 0–12 h | Paired measurements obtained within ICU hours 0–12 | 17249 | 3296 | 32.372 | 8 | <0.001 | Yes |
| eICU | First ICU stay per unique patient | First eligible ICU stay per unique patient | 19173 | 3690 | 52.018 | 8 | <0.001 | Yes |

*Note.* Analyses used complete-case Model 3b and varied the pairing rule, exposure window, or patient-level restriction. The eICU first-stay analysis retained only the first eligible ICU stay per unique patient. Overall P values are likelihood-ratio tests of the eight phenotype indicator terms. In MIMIC-IV, all chloride-bicarbonate pairs retained in the primary paired-laboratory cohort were recorded at the same chart time; consequently, the exact-time, ≤1-hour, and ≤3-hour restrictions yielded identical analytic samples and estimates.

**Supplementary Table 7B.** Exposure-definition and patient-level sensitivity analyses: phenotype-specific odds ratios.

| **Cohort** | **Analysis** | **Subset description** | **Contrast** | **Reference** | **n** | **Events** | **OR (95% CI)** | **P** | **Converged** |
| --- | --- | --- | --- | --- | --- | --- | --- | --- | --- |
| MIMIC-IV | Main analysis | Full paired analysis set | Low Cl + Low HCO₃ | Normal Cl + Normal HCO₃ | 13168 | 2370 | 1.136 (0.885–1.460) | 0.317 | Yes |
| MIMIC-IV | Main analysis | Full paired analysis set | Low Cl + Normal HCO₃ | Normal Cl + Normal HCO₃ | 13168 | 2370 | 1.519 (1.159–1.990) | 0.002 | Yes |
| MIMIC-IV | Main analysis | Full paired analysis set | Low Cl + High HCO₃ | Normal Cl + Normal HCO₃ | 13168 | 2370 | 0.989 (0.742–1.319) | 0.940 | Yes |
| MIMIC-IV | Main analysis | Full paired analysis set | Normal Cl + Low HCO₃ | Normal Cl + Normal HCO₃ | 13168 | 2370 | 0.963 (0.813–1.141) | 0.666 | Yes |
| MIMIC-IV | Main analysis | Full paired analysis set | Normal Cl + High HCO₃ | Normal Cl + Normal HCO₃ | 13168 | 2370 | 0.925 (0.736–1.162) | 0.502 | Yes |
| MIMIC-IV | Main analysis | Full paired analysis set | High Cl + Low HCO₃ | Normal Cl + Normal HCO₃ | 13168 | 2370 | 0.920 (0.770–1.099) | 0.356 | Yes |
| MIMIC-IV | Main analysis | Full paired analysis set | High Cl + Normal HCO₃ | Normal Cl + Normal HCO₃ | 13168 | 2370 | 0.841 (0.673–1.049) | 0.125 | Yes |
| MIMIC-IV | Main analysis | Full paired analysis set | High Cl + High HCO₃ | Normal Cl + Normal HCO₃ | 13168 | 2370 | 1.189 (0.773–1.827) | 0.430 | Yes |
| MIMIC-IV | Exact-time pairs | Exact-time Cl–HCO₃ pairs only | Low Cl + Low HCO₃ | Normal Cl + Normal HCO₃ | 13168 | 2370 | 1.136 (0.885–1.460) | 0.317 | Yes |
| MIMIC-IV | Exact-time pairs | Exact-time Cl–HCO₃ pairs only | Low Cl + Normal HCO₃ | Normal Cl + Normal HCO₃ | 13168 | 2370 | 1.519 (1.159–1.990) | 0.002 | Yes |
| MIMIC-IV | Exact-time pairs | Exact-time Cl–HCO₃ pairs only | Low Cl + High HCO₃ | Normal Cl + Normal HCO₃ | 13168 | 2370 | 0.989 (0.742–1.319) | 0.940 | Yes |
| MIMIC-IV | Exact-time pairs | Exact-time Cl–HCO₃ pairs only | Normal Cl + Low HCO₃ | Normal Cl + Normal HCO₃ | 13168 | 2370 | 0.963 (0.813–1.141) | 0.666 | Yes |
| MIMIC-IV | Exact-time pairs | Exact-time Cl–HCO₃ pairs only | Normal Cl + High HCO₃ | Normal Cl + Normal HCO₃ | 13168 | 2370 | 0.925 (0.736–1.162) | 0.502 | Yes |
| MIMIC-IV | Exact-time pairs | Exact-time Cl–HCO₃ pairs only | High Cl + Low HCO₃ | Normal Cl + Normal HCO₃ | 13168 | 2370 | 0.920 (0.770–1.099) | 0.356 | Yes |
| MIMIC-IV | Exact-time pairs | Exact-time Cl–HCO₃ pairs only | High Cl + Normal HCO₃ | Normal Cl + Normal HCO₃ | 13168 | 2370 | 0.841 (0.673–1.049) | 0.125 | Yes |
| MIMIC-IV | Exact-time pairs | Exact-time Cl–HCO₃ pairs only | High Cl + High HCO₃ | Normal Cl + Normal HCO₃ | 13168 | 2370 | 1.189 (0.773–1.827) | 0.430 | Yes |
| MIMIC-IV | Pairing interval ≤1 h | Chloride–bicarbonate measurement interval ≤1 h | Low Cl + Low HCO₃ | Normal Cl + Normal HCO₃ | 13168 | 2370 | 1.136 (0.885–1.460) | 0.317 | Yes |
| MIMIC-IV | Pairing interval ≤1 h | Chloride–bicarbonate measurement interval ≤1 h | Low Cl + Normal HCO₃ | Normal Cl + Normal HCO₃ | 13168 | 2370 | 1.519 (1.159–1.990) | 0.002 | Yes |
| MIMIC-IV | Pairing interval ≤1 h | Chloride–bicarbonate measurement interval ≤1 h | Low Cl + High HCO₃ | Normal Cl + Normal HCO₃ | 13168 | 2370 | 0.989 (0.742–1.319) | 0.940 | Yes |
| MIMIC-IV | Pairing interval ≤1 h | Chloride–bicarbonate measurement interval ≤1 h | Normal Cl + Low HCO₃ | Normal Cl + Normal HCO₃ | 13168 | 2370 | 0.963 (0.813–1.141) | 0.666 | Yes |
| MIMIC-IV | Pairing interval ≤1 h | Chloride–bicarbonate measurement interval ≤1 h | Normal Cl + High HCO₃ | Normal Cl + Normal HCO₃ | 13168 | 2370 | 0.925 (0.736–1.162) | 0.502 | Yes |
| MIMIC-IV | Pairing interval ≤1 h | Chloride–bicarbonate measurement interval ≤1 h | High Cl + Low HCO₃ | Normal Cl + Normal HCO₃ | 13168 | 2370 | 0.920 (0.770–1.099) | 0.356 | Yes |
| MIMIC-IV | Pairing interval ≤1 h | Chloride–bicarbonate measurement interval ≤1 h | High Cl + Normal HCO₃ | Normal Cl + Normal HCO₃ | 13168 | 2370 | 0.841 (0.673–1.049) | 0.125 | Yes |
| MIMIC-IV | Pairing interval ≤1 h | Chloride–bicarbonate measurement interval ≤1 h | High Cl + High HCO₃ | Normal Cl + Normal HCO₃ | 13168 | 2370 | 1.189 (0.773–1.827) | 0.430 | Yes |
| MIMIC-IV | Pairing interval ≤3 h | Chloride–bicarbonate measurement interval ≤3 h | Low Cl + Low HCO₃ | Normal Cl + Normal HCO₃ | 13168 | 2370 | 1.136 (0.885–1.460) | 0.317 | Yes |
| MIMIC-IV | Pairing interval ≤3 h | Chloride–bicarbonate measurement interval ≤3 h | Low Cl + Normal HCO₃ | Normal Cl + Normal HCO₃ | 13168 | 2370 | 1.519 (1.159–1.990) | 0.002 | Yes |
| MIMIC-IV | Pairing interval ≤3 h | Chloride–bicarbonate measurement interval ≤3 h | Low Cl + High HCO₃ | Normal Cl + Normal HCO₃ | 13168 | 2370 | 0.989 (0.742–1.319) | 0.940 | Yes |
| MIMIC-IV | Pairing interval ≤3 h | Chloride–bicarbonate measurement interval ≤3 h | Normal Cl + Low HCO₃ | Normal Cl + Normal HCO₃ | 13168 | 2370 | 0.963 (0.813–1.141) | 0.666 | Yes |
| MIMIC-IV | Pairing interval ≤3 h | Chloride–bicarbonate measurement interval ≤3 h | Normal Cl + High HCO₃ | Normal Cl + Normal HCO₃ | 13168 | 2370 | 0.925 (0.736–1.162) | 0.502 | Yes |
| MIMIC-IV | Pairing interval ≤3 h | Chloride–bicarbonate measurement interval ≤3 h | High Cl + Low HCO₃ | Normal Cl + Normal HCO₃ | 13168 | 2370 | 0.920 (0.770–1.099) | 0.356 | Yes |
| MIMIC-IV | Pairing interval ≤3 h | Chloride–bicarbonate measurement interval ≤3 h | High Cl + Normal HCO₃ | Normal Cl + Normal HCO₃ | 13168 | 2370 | 0.841 (0.673–1.049) | 0.125 | Yes |
| MIMIC-IV | Pairing interval ≤3 h | Chloride–bicarbonate measurement interval ≤3 h | High Cl + High HCO₃ | Normal Cl + Normal HCO₃ | 13168 | 2370 | 1.189 (0.773–1.827) | 0.430 | Yes |
| MIMIC-IV | Exposure window 0–6 h | Paired measurements obtained within ICU hours 0–6 | Low Cl + Low HCO₃ | Normal Cl + Normal HCO₃ | 11321 | 2034 | 1.118 (0.854–1.464) | 0.416 | Yes |
| MIMIC-IV | Exposure window 0–6 h | Paired measurements obtained within ICU hours 0–6 | Low Cl + Normal HCO₃ | Normal Cl + Normal HCO₃ | 11321 | 2034 | 1.604 (1.191–2.161) | 0.002 | Yes |
| MIMIC-IV | Exposure window 0–6 h | Paired measurements obtained within ICU hours 0–6 | Low Cl + High HCO₃ | Normal Cl + Normal HCO₃ | 11321 | 2034 | 1.123 (0.819–1.540) | 0.472 | Yes |
| MIMIC-IV | Exposure window 0–6 h | Paired measurements obtained within ICU hours 0–6 | Normal Cl + Low HCO₃ | Normal Cl + Normal HCO₃ | 11321 | 2034 | 1.002 (0.832–1.206) | 0.985 | Yes |
| MIMIC-IV | Exposure window 0–6 h | Paired measurements obtained within ICU hours 0–6 | Normal Cl + High HCO₃ | Normal Cl + Normal HCO₃ | 11321 | 2034 | 0.919 (0.706–1.197) | 0.532 | Yes |
| MIMIC-IV | Exposure window 0–6 h | Paired measurements obtained within ICU hours 0–6 | High Cl + Low HCO₃ | Normal Cl + Normal HCO₃ | 11321 | 2034 | 0.979 (0.807–1.188) | 0.833 | Yes |
| MIMIC-IV | Exposure window 0–6 h | Paired measurements obtained within ICU hours 0–6 | High Cl + Normal HCO₃ | Normal Cl + Normal HCO₃ | 11321 | 2034 | 0.898 (0.702–1.148) | 0.391 | Yes |
| MIMIC-IV | Exposure window 0–6 h | Paired measurements obtained within ICU hours 0–6 | High Cl + High HCO₃ | Normal Cl + Normal HCO₃ | 11321 | 2034 | 1.108 (0.674–1.823) | 0.685 | Yes |
| MIMIC-IV | Exposure window 0–12 h | Paired measurements obtained within ICU hours 0–12 | Low Cl + Low HCO₃ | Normal Cl + Normal HCO₃ | 12775 | 2289 | 1.114 (0.864–1.438) | 0.405 | Yes |
| MIMIC-IV | Exposure window 0–12 h | Paired measurements obtained within ICU hours 0–12 | Low Cl + Normal HCO₃ | Normal Cl + Normal HCO₃ | 12775 | 2289 | 1.499 (1.137–1.976) | 0.004 | Yes |
| MIMIC-IV | Exposure window 0–12 h | Paired measurements obtained within ICU hours 0–12 | Low Cl + High HCO₃ | Normal Cl + Normal HCO₃ | 12775 | 2289 | 1.049 (0.780–1.411) | 0.753 | Yes |
| MIMIC-IV | Exposure window 0–12 h | Paired measurements obtained within ICU hours 0–12 | Normal Cl + Low HCO₃ | Normal Cl + Normal HCO₃ | 12775 | 2289 | 0.979 (0.823–1.164) | 0.811 | Yes |
| MIMIC-IV | Exposure window 0–12 h | Paired measurements obtained within ICU hours 0–12 | Normal Cl + High HCO₃ | Normal Cl + Normal HCO₃ | 12775 | 2289 | 0.910 (0.717–1.155) | 0.438 | Yes |
| MIMIC-IV | Exposure window 0–12 h | Paired measurements obtained within ICU hours 0–12 | High Cl + Low HCO₃ | Normal Cl + Normal HCO₃ | 12775 | 2289 | 0.939 (0.784–1.126) | 0.498 | Yes |
| MIMIC-IV | Exposure window 0–12 h | Paired measurements obtained within ICU hours 0–12 | High Cl + Normal HCO₃ | Normal Cl + Normal HCO₃ | 12775 | 2289 | 0.863 (0.687–1.084) | 0.204 | Yes |
| MIMIC-IV | Exposure window 0–12 h | Paired measurements obtained within ICU hours 0–12 | High Cl + High HCO₃ | Normal Cl + Normal HCO₃ | 12775 | 2289 | 1.308 (0.843–2.030) | 0.232 | Yes |
| eICU | Main analysis | Full paired analysis set | Low Cl + Low HCO₃ | Normal Cl + Normal HCO₃ | 21414 | 3907 | 1.426 (1.169–1.739) | <0.001 | Yes |
| eICU | Main analysis | Full paired analysis set | Low Cl + Normal HCO₃ | Normal Cl + Normal HCO₃ | 21414 | 3907 | 1.399 (1.129–1.733) | 0.002 | Yes |
| eICU | Main analysis | Full paired analysis set | Low Cl + High HCO₃ | Normal Cl + Normal HCO₃ | 21414 | 3907 | 1.166 (0.952–1.430) | 0.138 | Yes |
| eICU | Main analysis | Full paired analysis set | Normal Cl + Low HCO₃ | Normal Cl + Normal HCO₃ | 21414 | 3907 | 1.207 (1.053–1.384) | 0.007 | Yes |
| eICU | Main analysis | Full paired analysis set | Normal Cl + High HCO₃ | Normal Cl + Normal HCO₃ | 21414 | 3907 | 1.100 (0.940–1.288) | 0.233 | Yes |
| eICU | Main analysis | Full paired analysis set | High Cl + Low HCO₃ | Normal Cl + Normal HCO₃ | 21414 | 3907 | 1.084 (0.949–1.238) | 0.233 | Yes |
| eICU | Main analysis | Full paired analysis set | High Cl + Normal HCO₃ | Normal Cl + Normal HCO₃ | 21414 | 3907 | 0.758 (0.641–0.895) | 0.001 | Yes |
| eICU | Main analysis | Full paired analysis set | High Cl + High HCO₃ | Normal Cl + Normal HCO₃ | 21414 | 3907 | 0.903 (0.693–1.176) | 0.448 | Yes |
| eICU | Exact-time pairs | Exact-time Cl–HCO₃ pairs only | Low Cl + Low HCO₃ | Normal Cl + Normal HCO₃ | 21246 | 3858 | 1.408 (1.153–1.720) | <0.001 | Yes |
| eICU | Exact-time pairs | Exact-time Cl–HCO₃ pairs only | Low Cl + Normal HCO₃ | Normal Cl + Normal HCO₃ | 21246 | 3858 | 1.403 (1.132–1.740) | 0.002 | Yes |
| eICU | Exact-time pairs | Exact-time Cl–HCO₃ pairs only | Low Cl + High HCO₃ | Normal Cl + Normal HCO₃ | 21246 | 3858 | 1.162 (0.948–1.426) | 0.149 | Yes |
| eICU | Exact-time pairs | Exact-time Cl–HCO₃ pairs only | Normal Cl + Low HCO₃ | Normal Cl + Normal HCO₃ | 21246 | 3858 | 1.196 (1.042–1.372) | 0.011 | Yes |
| eICU | Exact-time pairs | Exact-time Cl–HCO₃ pairs only | Normal Cl + High HCO₃ | Normal Cl + Normal HCO₃ | 21246 | 3858 | 1.101 (0.941–1.289) | 0.229 | Yes |
| eICU | Exact-time pairs | Exact-time Cl–HCO₃ pairs only | High Cl + Low HCO₃ | Normal Cl + Normal HCO₃ | 21246 | 3858 | 1.078 (0.943–1.232) | 0.272 | Yes |
| eICU | Exact-time pairs | Exact-time Cl–HCO₃ pairs only | High Cl + Normal HCO₃ | Normal Cl + Normal HCO₃ | 21246 | 3858 | 0.749 (0.634–0.886) | <0.001 | Yes |
| eICU | Exact-time pairs | Exact-time Cl–HCO₃ pairs only | High Cl + High HCO₃ | Normal Cl + Normal HCO₃ | 21246 | 3858 | 0.904 (0.694–1.179) | 0.457 | Yes |
| eICU | Pairing interval ≤1 h | Chloride–bicarbonate measurement interval ≤1 h | Low Cl + Low HCO₃ | Normal Cl + Normal HCO₃ | 21295 | 3875 | 1.407 (1.153–1.718) | <0.001 | Yes |
| eICU | Pairing interval ≤1 h | Chloride–bicarbonate measurement interval ≤1 h | Low Cl + Normal HCO₃ | Normal Cl + Normal HCO₃ | 21295 | 3875 | 1.414 (1.142–1.752) | 0.002 | Yes |
| eICU | Pairing interval ≤1 h | Chloride–bicarbonate measurement interval ≤1 h | Low Cl + High HCO₃ | Normal Cl + Normal HCO₃ | 21295 | 3875 | 1.159 (0.945–1.421) | 0.157 | Yes |
| eICU | Pairing interval ≤1 h | Chloride–bicarbonate measurement interval ≤1 h | Normal Cl + Low HCO₃ | Normal Cl + Normal HCO₃ | 21295 | 3875 | 1.196 (1.043–1.372) | 0.011 | Yes |
| eICU | Pairing interval ≤1 h | Chloride–bicarbonate measurement interval ≤1 h | Normal Cl + High HCO₃ | Normal Cl + Normal HCO₃ | 21295 | 3875 | 1.097 (0.937–1.284) | 0.249 | Yes |
| eICU | Pairing interval ≤1 h | Chloride–bicarbonate measurement interval ≤1 h | High Cl + Low HCO₃ | Normal Cl + Normal HCO₃ | 21295 | 3875 | 1.078 (0.944–1.232) | 0.267 | Yes |
| eICU | Pairing interval ≤1 h | Chloride–bicarbonate measurement interval ≤1 h | High Cl + Normal HCO₃ | Normal Cl + Normal HCO₃ | 21295 | 3875 | 0.753 (0.637–0.890) | <0.001 | Yes |
| eICU | Pairing interval ≤1 h | Chloride–bicarbonate measurement interval ≤1 h | High Cl + High HCO₃ | Normal Cl + Normal HCO₃ | 21295 | 3875 | 0.905 (0.695–1.180) | 0.461 | Yes |
| eICU | Pairing interval ≤3 h | Chloride–bicarbonate measurement interval ≤3 h | Low Cl + Low HCO₃ | Normal Cl + Normal HCO₃ | 21355 | 3892 | 1.413 (1.158–1.724) | <0.001 | Yes |
| eICU | Pairing interval ≤3 h | Chloride–bicarbonate measurement interval ≤3 h | Low Cl + Normal HCO₃ | Normal Cl + Normal HCO₃ | 21355 | 3892 | 1.414 (1.141–1.751) | 0.002 | Yes |
| eICU | Pairing interval ≤3 h | Chloride–bicarbonate measurement interval ≤3 h | Low Cl + High HCO₃ | Normal Cl + Normal HCO₃ | 21355 | 3892 | 1.167 (0.952–1.431) | 0.136 | Yes |
| eICU | Pairing interval ≤3 h | Chloride–bicarbonate measurement interval ≤3 h | Normal Cl + Low HCO₃ | Normal Cl + Normal HCO₃ | 21355 | 3892 | 1.201 (1.047–1.377) | 0.009 | Yes |
| eICU | Pairing interval ≤3 h | Chloride–bicarbonate measurement interval ≤3 h | Normal Cl + High HCO₃ | Normal Cl + Normal HCO₃ | 21355 | 3892 | 1.100 (0.940–1.287) | 0.235 | Yes |
| eICU | Pairing interval ≤3 h | Chloride–bicarbonate measurement interval ≤3 h | High Cl + Low HCO₃ | Normal Cl + Normal HCO₃ | 21355 | 3892 | 1.085 (0.950–1.240) | 0.228 | Yes |
| eICU | Pairing interval ≤3 h | Chloride–bicarbonate measurement interval ≤3 h | High Cl + Normal HCO₃ | Normal Cl + Normal HCO₃ | 21355 | 3892 | 0.756 (0.640–0.893) | 0.001 | Yes |
| eICU | Pairing interval ≤3 h | Chloride–bicarbonate measurement interval ≤3 h | High Cl + High HCO₃ | Normal Cl + Normal HCO₃ | 21355 | 3892 | 0.900 (0.691–1.173) | 0.436 | Yes |
| eICU | Exposure window 0–6 h | Paired measurements obtained within ICU hours 0–6 | Low Cl + Low HCO₃ | Normal Cl + Normal HCO₃ | 11980 | 2414 | 1.173 (0.919–1.498) | 0.200 | Yes |
| eICU | Exposure window 0–6 h | Paired measurements obtained within ICU hours 0–6 | Low Cl + Normal HCO₃ | Normal Cl + Normal HCO₃ | 11980 | 2414 | 1.284 (0.981–1.680) | 0.069 | Yes |
| eICU | Exposure window 0–6 h | Paired measurements obtained within ICU hours 0–6 | Low Cl + High HCO₃ | Normal Cl + Normal HCO₃ | 11980 | 2414 | 0.928 (0.701–1.229) | 0.603 | Yes |
| eICU | Exposure window 0–6 h | Paired measurements obtained within ICU hours 0–6 | Normal Cl + Low HCO₃ | Normal Cl + Normal HCO₃ | 11980 | 2414 | 1.072 (0.898–1.280) | 0.443 | Yes |
| eICU | Exposure window 0–6 h | Paired measurements obtained within ICU hours 0–6 | Normal Cl + High HCO₃ | Normal Cl + Normal HCO₃ | 11980 | 2414 | 1.158 (0.929–1.443) | 0.192 | Yes |
| eICU | Exposure window 0–6 h | Paired measurements obtained within ICU hours 0–6 | High Cl + Low HCO₃ | Normal Cl + Normal HCO₃ | 11980 | 2414 | 1.071 (0.898–1.277) | 0.445 | Yes |
| eICU | Exposure window 0–6 h | Paired measurements obtained within ICU hours 0–6 | High Cl + Normal HCO₃ | Normal Cl + Normal HCO₃ | 11980 | 2414 | 0.751 (0.595–0.946) | 0.015 | Yes |
| eICU | Exposure window 0–6 h | Paired measurements obtained within ICU hours 0–6 | High Cl + High HCO₃ | Normal Cl + Normal HCO₃ | 11980 | 2414 | 1.079 (0.757–1.537) | 0.675 | Yes |
| eICU | Exposure window 0–12 h | Paired measurements obtained within ICU hours 0–12 | Low Cl + Low HCO₃ | Normal Cl + Normal HCO₃ | 17249 | 3296 | 1.272 (1.028–1.575) | 0.027 | Yes |
| eICU | Exposure window 0–12 h | Paired measurements obtained within ICU hours 0–12 | Low Cl + Normal HCO₃ | Normal Cl + Normal HCO₃ | 17249 | 3296 | 1.339 (1.063–1.687) | 0.013 | Yes |
| eICU | Exposure window 0–12 h | Paired measurements obtained within ICU hours 0–12 | Low Cl + High HCO₃ | Normal Cl + Normal HCO₃ | 17249 | 3296 | 1.099 (0.874–1.380) | 0.420 | Yes |
| eICU | Exposure window 0–12 h | Paired measurements obtained within ICU hours 0–12 | Normal Cl + Low HCO₃ | Normal Cl + Normal HCO₃ | 17249 | 3296 | 1.128 (0.971–1.311) | 0.116 | Yes |
| eICU | Exposure window 0–12 h | Paired measurements obtained within ICU hours 0–12 | Normal Cl + High HCO₃ | Normal Cl + Normal HCO₃ | 17249 | 3296 | 1.111 (0.929–1.330) | 0.248 | Yes |
| eICU | Exposure window 0–12 h | Paired measurements obtained within ICU hours 0–12 | High Cl + Low HCO₃ | Normal Cl + Normal HCO₃ | 17249 | 3296 | 1.061 (0.916–1.228) | 0.432 | Yes |
| eICU | Exposure window 0–12 h | Paired measurements obtained within ICU hours 0–12 | High Cl + Normal HCO₃ | Normal Cl + Normal HCO₃ | 17249 | 3296 | 0.744 (0.615–0.900) | 0.002 | Yes |
| eICU | Exposure window 0–12 h | Paired measurements obtained within ICU hours 0–12 | High Cl + High HCO₃ | Normal Cl + Normal HCO₃ | 17249 | 3296 | 0.950 (0.704–1.281) | 0.736 | Yes |
| eICU | First ICU stay per unique patient | First eligible ICU stay per unique patient | Low Cl + Low HCO₃ | Normal Cl + Normal HCO₃ | 19173 | 3690 | 1.483 (1.208–1.822) | <0.001 | Yes |
| eICU | First ICU stay per unique patient | First eligible ICU stay per unique patient | Low Cl + Normal HCO₃ | Normal Cl + Normal HCO₃ | 19173 | 3690 | 1.437 (1.149–1.798) | 0.001 | Yes |
| eICU | First ICU stay per unique patient | First eligible ICU stay per unique patient | Low Cl + High HCO₃ | Normal Cl + Normal HCO₃ | 19173 | 3690 | 1.206 (0.974–1.493) | 0.085 | Yes |
| eICU | First ICU stay per unique patient | First eligible ICU stay per unique patient | Normal Cl + Low HCO₃ | Normal Cl + Normal HCO₃ | 19173 | 3690 | 1.209 (1.049–1.394) | 0.009 | Yes |
| eICU | First ICU stay per unique patient | First eligible ICU stay per unique patient | Normal Cl + High HCO₃ | Normal Cl + Normal HCO₃ | 19173 | 3690 | 1.131 (0.959–1.334) | 0.142 | Yes |
| eICU | First ICU stay per unique patient | First eligible ICU stay per unique patient | High Cl + Low HCO₃ | Normal Cl + Normal HCO₃ | 19173 | 3690 | 1.077 (0.937–1.237) | 0.297 | Yes |
| eICU | First ICU stay per unique patient | First eligible ICU stay per unique patient | High Cl + Normal HCO₃ | Normal Cl + Normal HCO₃ | 19173 | 3690 | 0.775 (0.652–0.921) | 0.004 | Yes |
| eICU | First ICU stay per unique patient | First eligible ICU stay per unique patient | High Cl + High HCO₃ | Normal Cl + Normal HCO₃ | 19173 | 3690 | 0.865 (0.655–1.141) | 0.304 | Yes |

*Note.* Analyses used complete-case Model 3b. The reference group is normal chloride plus normal bicarbonate. Pairing interval denotes the absolute time difference between chloride and bicarbonate measurements. The eICU first-stay analysis retained only the first eligible ICU stay per unique patient. In MIMIC-IV, all pairs retained in the primary paired-laboratory cohort were recorded at the same chart time; consequently, the exact-time, ≤1-hour, and ≤3-hour restrictions yielded identical analytic samples and estimates.

**Supplementary Table 8A.** Exploratory subgroup interaction tests.

| **Cohort** | **Subgroup** | **n** | **Events** | **No: n** | **No: events** | **Yes: n** | **Yes: events** | **Interaction chi-square** | **df** | **Interaction P** | **Reliably estimable** | **Note** |
| --- | --- | --- | --- | --- | --- | --- | --- | --- | --- | --- | --- | --- |
| MIMIC-IV | Acute kidney injury | 13168 | 2370 | 2244 | 151 | 10924 | 2219 | 5.303 | 8 | 0.725 | Yes |  |
| MIMIC-IV | Septic shock | 13168 | 2370 | 8423 | 1270 | 4745 | 1100 | 13.462 | 8 | 0.097 | Yes |  |
| MIMIC-IV | Sodium bicarbonate therapy | 13168 | 2370 | 11750 | 1737 | 1418 | 633 | NE | 8 | NE | No | Not reliably estimable: sparse treated high-Cl/high-HCO₃ cell (n = 5; deaths = 0). |
| MIMIC-IV | Vasoactive therapy | 13168 | 2370 | 4280 | 639 | 8888 | 1731 | 10.523 | 8 | 0.230 | Yes |  |
| eICU | Acute kidney injury | 21414 | 3907 | 9533 | 944 | 11881 | 2963 | 18.957 | 8 | 0.015 | Yes |  |
| eICU | Septic shock | 21414 | 3907 | 20400 | 3466 | 1014 | 441 | 15.418 | 8 | 0.052 | Yes |  |
| eICU | Sodium bicarbonate therapy | 21414 | 3907 | 18806 | 2906 | 2608 | 1001 | 15.131 | 8 | 0.057 | Yes |  |
| eICU | Vasoactive therapy | 21414 | 3907 | 15668 | 2112 | 5746 | 1795 | 4.786 | 8 | 0.780 | Yes |  |

*Note.* Interaction P values are 8-df Wald tests from joint phenotype-by-subgroup models. The MIMIC-IV sodium bicarbonate therapy interaction was not reliably estimable because a treated high-chloride/high-bicarbonate cell contained no deaths.

**Supplementary Table 8B.** Exploratory subgroup-specific odds ratios.

| **Cohort** | **Subgroup** | **Subgroup level** | **Contrast** | **Reference** | **Stratum n** | **Stratum events** | **OR (95% CI)** | **P** |
| --- | --- | --- | --- | --- | --- | --- | --- | --- |
| MIMIC-IV | Acute kidney injury | No | Low Cl + Low HCO₃ | Normal Cl + Normal HCO₃ | 2244 | 151 | 2.506 (0.876–7.172) | 0.087 |
| MIMIC-IV | Acute kidney injury | Yes | Low Cl + Low HCO₃ | Normal Cl + Normal HCO₃ | 10924 | 2219 | 1.086 (0.841–1.403) | 0.525 |
| MIMIC-IV | Acute kidney injury | No | Low Cl + Normal HCO₃ | Normal Cl + Normal HCO₃ | 2244 | 151 | 2.437 (0.888–6.688) | 0.084 |
| MIMIC-IV | Acute kidney injury | Yes | Low Cl + Normal HCO₃ | Normal Cl + Normal HCO₃ | 10924 | 2219 | 1.460 (1.103–1.932) | 0.008 |
| MIMIC-IV | Acute kidney injury | No | Low Cl + High HCO₃ | Normal Cl + Normal HCO₃ | 2244 | 151 | 1.858 (0.701–4.924) | 0.213 |
| MIMIC-IV | Acute kidney injury | Yes | Low Cl + High HCO₃ | Normal Cl + Normal HCO₃ | 10924 | 2219 | 0.935 (0.692–1.262) | 0.659 |
| MIMIC-IV | Acute kidney injury | No | Normal Cl + Low HCO₃ | Normal Cl + Normal HCO₃ | 2244 | 151 | 1.248 (0.666–2.339) | 0.489 |
| MIMIC-IV | Acute kidney injury | Yes | Normal Cl + Low HCO₃ | Normal Cl + Normal HCO₃ | 10924 | 2219 | 0.949 (0.796–1.131) | 0.560 |
| MIMIC-IV | Acute kidney injury | No | Normal Cl + High HCO₃ | Normal Cl + Normal HCO₃ | 2244 | 151 | 1.354 (0.599–3.059) | 0.466 |
| MIMIC-IV | Acute kidney injury | Yes | Normal Cl + High HCO₃ | Normal Cl + Normal HCO₃ | 10924 | 2219 | 0.892 (0.703–1.132) | 0.347 |
| MIMIC-IV | Acute kidney injury | No | High Cl + Low HCO₃ | Normal Cl + Normal HCO₃ | 2244 | 151 | 1.321 (0.727–2.398) | 0.361 |
| MIMIC-IV | Acute kidney injury | Yes | High Cl + Low HCO₃ | Normal Cl + Normal HCO₃ | 10924 | 2219 | 0.899 (0.747–1.083) | 0.262 |
| MIMIC-IV | Acute kidney injury | No | High Cl + Normal HCO₃ | Normal Cl + Normal HCO₃ | 2244 | 151 | 0.809 (0.383–1.706) | 0.577 |
| MIMIC-IV | Acute kidney injury | Yes | High Cl + Normal HCO₃ | Normal Cl + Normal HCO₃ | 10924 | 2219 | 0.866 (0.686–1.093) | 0.225 |
| MIMIC-IV | Acute kidney injury | No | High Cl + High HCO₃ | Normal Cl + Normal HCO₃ | 2244 | 151 | 1.683 (0.475–5.960) | 0.420 |
| MIMIC-IV | Acute kidney injury | Yes | High Cl + High HCO₃ | Normal Cl + Normal HCO₃ | 10924 | 2219 | 1.157 (0.733–1.826) | 0.532 |
| MIMIC-IV | Septic shock | No | Low Cl + Low HCO₃ | Normal Cl + Normal HCO₃ | 8423 | 1270 | 0.978 (0.712–1.344) | 0.893 |
| MIMIC-IV | Septic shock | Yes | Low Cl + Low HCO₃ | Normal Cl + Normal HCO₃ | 4745 | 1100 | 1.405 (0.949–2.079) | 0.089 |
| MIMIC-IV | Septic shock | No | Low Cl + Normal HCO₃ | Normal Cl + Normal HCO₃ | 8423 | 1270 | 1.550 (1.121–2.145) | 0.008 |
| MIMIC-IV | Septic shock | Yes | Low Cl + Normal HCO₃ | Normal Cl + Normal HCO₃ | 4745 | 1100 | 1.467 (0.908–2.372) | 0.118 |
| MIMIC-IV | Septic shock | No | Low Cl + High HCO₃ | Normal Cl + Normal HCO₃ | 8423 | 1270 | 0.907 (0.651–1.264) | 0.566 |
| MIMIC-IV | Septic shock | Yes | Low Cl + High HCO₃ | Normal Cl + Normal HCO₃ | 4745 | 1100 | 1.315 (0.733–2.360) | 0.359 |
| MIMIC-IV | Septic shock | No | Normal Cl + Low HCO₃ | Normal Cl + Normal HCO₃ | 8423 | 1270 | 0.944 (0.766–1.163) | 0.586 |
| MIMIC-IV | Septic shock | Yes | Normal Cl + Low HCO₃ | Normal Cl + Normal HCO₃ | 4745 | 1100 | 1.001 (0.751–1.334) | 0.997 |
| MIMIC-IV | Septic shock | No | Normal Cl + High HCO₃ | Normal Cl + Normal HCO₃ | 8423 | 1270 | 0.836 (0.645–1.084) | 0.177 |
| MIMIC-IV | Septic shock | Yes | Normal Cl + High HCO₃ | Normal Cl + Normal HCO₃ | 4745 | 1100 | 1.439 (0.878–2.359) | 0.148 |
| MIMIC-IV | Septic shock | No | High Cl + Low HCO₃ | Normal Cl + Normal HCO₃ | 8423 | 1270 | 0.954 (0.764–1.193) | 0.682 |
| MIMIC-IV | Septic shock | Yes | High Cl + Low HCO₃ | Normal Cl + Normal HCO₃ | 4745 | 1100 | 0.892 (0.664–1.199) | 0.448 |
| MIMIC-IV | Septic shock | No | High Cl + Normal HCO₃ | Normal Cl + Normal HCO₃ | 8423 | 1270 | 0.917 (0.706–1.190) | 0.513 |
| MIMIC-IV | Septic shock | Yes | High Cl + Normal HCO₃ | Normal Cl + Normal HCO₃ | 4745 | 1100 | 0.697 (0.465–1.044) | 0.080 |
| MIMIC-IV | Septic shock | No | High Cl + High HCO₃ | Normal Cl + Normal HCO₃ | 8423 | 1270 | 1.393 (0.867–2.239) | 0.171 |
| MIMIC-IV | Septic shock | Yes | High Cl + High HCO₃ | Normal Cl + Normal HCO₃ | 4745 | 1100 | 0.611 (0.216–1.730) | 0.354 |
| MIMIC-IV | Sodium bicarbonate therapy | No | Low Cl + Low HCO₃ | Normal Cl + Normal HCO₃ | 11750 | 1737 | 1.264 (0.956–1.673) | 0.100 |
| MIMIC-IV | Sodium bicarbonate therapy | Yes | Low Cl + Low HCO₃ | Normal Cl + Normal HCO₃ | 1418 | 633 | 0.787 (0.424–1.458) | 0.446 |
| MIMIC-IV | Sodium bicarbonate therapy | No | Low Cl + Normal HCO₃ | Normal Cl + Normal HCO₃ | 11750 | 1737 | 1.518 (1.148–2.009) | 0.003 |
| MIMIC-IV | Sodium bicarbonate therapy | Yes | Low Cl + Normal HCO₃ | Normal Cl + Normal HCO₃ | 1418 | 633 | 1.505 (0.520–4.356) | 0.451 |
| MIMIC-IV | Sodium bicarbonate therapy | No | Low Cl + High HCO₃ | Normal Cl + Normal HCO₃ | 11750 | 1737 | 0.972 (0.723–1.307) | 0.852 |
| MIMIC-IV | Sodium bicarbonate therapy | Yes | Low Cl + High HCO₃ | Normal Cl + Normal HCO₃ | 1418 | 633 | 1.533 (0.409–5.740) | 0.526 |
| MIMIC-IV | Sodium bicarbonate therapy | No | Normal Cl + Low HCO₃ | Normal Cl + Normal HCO₃ | 11750 | 1737 | 0.957 (0.799–1.147) | 0.637 |
| MIMIC-IV | Sodium bicarbonate therapy | Yes | Normal Cl + Low HCO₃ | Normal Cl + Normal HCO₃ | 1418 | 633 | 0.866 (0.501–1.496) | 0.605 |
| MIMIC-IV | Sodium bicarbonate therapy | No | Normal Cl + High HCO₃ | Normal Cl + Normal HCO₃ | 11750 | 1737 | 0.926 (0.732–1.172) | 0.523 |
| MIMIC-IV | Sodium bicarbonate therapy | Yes | Normal Cl + High HCO₃ | Normal Cl + Normal HCO₃ | 1418 | 633 | 0.943 (0.347–2.558) | 0.908 |
| MIMIC-IV | Sodium bicarbonate therapy | No | High Cl + Low HCO₃ | Normal Cl + Normal HCO₃ | 11750 | 1737 | 0.927 (0.765–1.124) | 0.439 |
| MIMIC-IV | Sodium bicarbonate therapy | Yes | High Cl + Low HCO₃ | Normal Cl + Normal HCO₃ | 1418 | 633 | 0.792 (0.457–1.373) | 0.406 |
| MIMIC-IV | Sodium bicarbonate therapy | No | High Cl + Normal HCO₃ | Normal Cl + Normal HCO₃ | 11750 | 1737 | 0.855 (0.680–1.074) | 0.179 |
| MIMIC-IV | Sodium bicarbonate therapy | Yes | High Cl + Normal HCO₃ | Normal Cl + Normal HCO₃ | 1418 | 633 | 0.641 (0.265–1.553) | 0.325 |
| MIMIC-IV | Sodium bicarbonate therapy | No | High Cl + High HCO₃ | Normal Cl + Normal HCO₃ | 11750 | 1737 | 1.289 (0.837–1.986) | 0.250 |
| MIMIC-IV | Sodium bicarbonate therapy | Yes | High Cl + High HCO₃ | Normal Cl + Normal HCO₃ | 1418 | 633 | Not estimable | NE |
| MIMIC-IV | Vasoactive therapy | No | Low Cl + Low HCO₃ | Normal Cl + Normal HCO₃ | 4280 | 639 | 0.838 (0.529–1.327) | 0.451 |
| MIMIC-IV | Vasoactive therapy | Yes | Low Cl + Low HCO₃ | Normal Cl + Normal HCO₃ | 8888 | 1731 | 1.307 (0.972–1.757) | 0.077 |
| MIMIC-IV | Vasoactive therapy | No | Low Cl + Normal HCO₃ | Normal Cl + Normal HCO₃ | 4280 | 639 | 1.481 (0.981–2.236) | 0.062 |
| MIMIC-IV | Vasoactive therapy | Yes | Low Cl + Normal HCO₃ | Normal Cl + Normal HCO₃ | 8888 | 1731 | 1.557 (1.093–2.218) | 0.014 |
| MIMIC-IV | Vasoactive therapy | No | Low Cl + High HCO₃ | Normal Cl + Normal HCO₃ | 4280 | 639 | 0.814 (0.529–1.253) | 0.350 |
| MIMIC-IV | Vasoactive therapy | Yes | Low Cl + High HCO₃ | Normal Cl + Normal HCO₃ | 8888 | 1731 | 1.157 (0.787–1.702) | 0.458 |
| MIMIC-IV | Vasoactive therapy | No | Normal Cl + Low HCO₃ | Normal Cl + Normal HCO₃ | 4280 | 639 | 0.781 (0.584–1.045) | 0.096 |
| MIMIC-IV | Vasoactive therapy | Yes | Normal Cl + Low HCO₃ | Normal Cl + Normal HCO₃ | 8888 | 1731 | 1.072 (0.869–1.322) | 0.517 |
| MIMIC-IV | Vasoactive therapy | No | Normal Cl + High HCO₃ | Normal Cl + Normal HCO₃ | 4280 | 639 | 0.894 (0.651–1.229) | 0.491 |
| MIMIC-IV | Vasoactive therapy | Yes | Normal Cl + High HCO₃ | Normal Cl + Normal HCO₃ | 8888 | 1731 | 0.930 (0.670–1.292) | 0.666 |
| MIMIC-IV | Vasoactive therapy | No | High Cl + Low HCO₃ | Normal Cl + Normal HCO₃ | 4280 | 639 | 0.916 (0.676–1.241) | 0.572 |
| MIMIC-IV | Vasoactive therapy | Yes | High Cl + Low HCO₃ | Normal Cl + Normal HCO₃ | 8888 | 1731 | 0.955 (0.767–1.188) | 0.679 |
| MIMIC-IV | Vasoactive therapy | No | High Cl + Normal HCO₃ | Normal Cl + Normal HCO₃ | 4280 | 639 | 0.941 (0.664–1.334) | 0.733 |
| MIMIC-IV | Vasoactive therapy | Yes | High Cl + Normal HCO₃ | Normal Cl + Normal HCO₃ | 8888 | 1731 | 0.795 (0.598–1.056) | 0.113 |
| MIMIC-IV | Vasoactive therapy | No | High Cl + High HCO₃ | Normal Cl + Normal HCO₃ | 4280 | 639 | 0.819 (0.434–1.545) | 0.537 |
| MIMIC-IV | Vasoactive therapy | Yes | High Cl + High HCO₃ | Normal Cl + Normal HCO₃ | 8888 | 1731 | 1.676 (0.935–3.006) | 0.083 |
| eICU | Acute kidney injury | No | Low Cl + Low HCO₃ | Normal Cl + Normal HCO₃ | 9533 | 944 | 2.383 (1.632–3.480) | <0.001 |
| eICU | Acute kidney injury | Yes | Low Cl + Low HCO₃ | Normal Cl + Normal HCO₃ | 11881 | 2963 | 1.173 (0.935–1.471) | 0.169 |
| eICU | Acute kidney injury | No | Low Cl + Normal HCO₃ | Normal Cl + Normal HCO₃ | 9533 | 944 | 1.474 (0.954–2.277) | 0.081 |
| eICU | Acute kidney injury | Yes | Low Cl + Normal HCO₃ | Normal Cl + Normal HCO₃ | 11881 | 2963 | 1.331 (1.038–1.706) | 0.024 |
| eICU | Acute kidney injury | No | Low Cl + High HCO₃ | Normal Cl + Normal HCO₃ | 9533 | 944 | 1.469 (0.997–2.166) | 0.052 |
| eICU | Acute kidney injury | Yes | Low Cl + High HCO₃ | Normal Cl + Normal HCO₃ | 11881 | 2963 | 1.039 (0.818–1.320) | 0.755 |
| eICU | Acute kidney injury | No | Normal Cl + Low HCO₃ | Normal Cl + Normal HCO₃ | 9533 | 944 | 1.676 (1.295–2.169) | <0.001 |
| eICU | Acute kidney injury | Yes | Normal Cl + Low HCO₃ | Normal Cl + Normal HCO₃ | 11881 | 2963 | 1.054 (0.899–1.236) | 0.516 |
| eICU | Acute kidney injury | No | Normal Cl + High HCO₃ | Normal Cl + Normal HCO₃ | 9533 | 944 | 1.482 (1.120–1.961) | 0.006 |
| eICU | Acute kidney injury | Yes | Normal Cl + High HCO₃ | Normal Cl + Normal HCO₃ | 11881 | 2963 | 0.974 (0.804–1.180) | 0.786 |
| eICU | Acute kidney injury | No | High Cl + Low HCO₃ | Normal Cl + Normal HCO₃ | 9533 | 944 | 1.438 (1.126–1.838) | 0.004 |
| eICU | Acute kidney injury | Yes | High Cl + Low HCO₃ | Normal Cl + Normal HCO₃ | 11881 | 2963 | 0.979 (0.837–1.146) | 0.791 |
| eICU | Acute kidney injury | No | High Cl + Normal HCO₃ | Normal Cl + Normal HCO₃ | 9533 | 944 | 0.846 (0.622–1.150) | 0.285 |
| eICU | Acute kidney injury | Yes | High Cl + Normal HCO₃ | Normal Cl + Normal HCO₃ | 11881 | 2963 | 0.753 (0.615–0.921) | 0.006 |
| eICU | Acute kidney injury | No | High Cl + High HCO₃ | Normal Cl + Normal HCO₃ | 9533 | 944 | 0.969 (0.603–1.556) | 0.896 |
| eICU | Acute kidney injury | Yes | High Cl + High HCO₃ | Normal Cl + Normal HCO₃ | 11881 | 2963 | 0.923 (0.669–1.275) | 0.629 |
| eICU | Septic shock | No | Low Cl + Low HCO₃ | Normal Cl + Normal HCO₃ | 20400 | 3466 | 1.523 (1.238–1.874) | <0.001 |
| eICU | Septic shock | Yes | Low Cl + Low HCO₃ | Normal Cl + Normal HCO₃ | 1014 | 441 | 0.746 (0.388–1.432) | 0.378 |
| eICU | Septic shock | No | Low Cl + Normal HCO₃ | Normal Cl + Normal HCO₃ | 20400 | 3466 | 1.373 (1.101–1.713) | 0.005 |
| eICU | Septic shock | Yes | Low Cl + Normal HCO₃ | Normal Cl + Normal HCO₃ | 1014 | 441 | 1.871 (0.729–4.803) | 0.193 |
| eICU | Septic shock | No | Low Cl + High HCO₃ | Normal Cl + Normal HCO₃ | 20400 | 3466 | 1.193 (0.970–1.467) | 0.094 |
| eICU | Septic shock | Yes | Low Cl + High HCO₃ | Normal Cl + Normal HCO₃ | 1014 | 441 | 0.642 (0.193–2.143) | 0.472 |
| eICU | Septic shock | No | Normal Cl + Low HCO₃ | Normal Cl + Normal HCO₃ | 20400 | 3466 | 1.242 (1.079–1.431) | 0.003 |
| eICU | Septic shock | Yes | Normal Cl + Low HCO₃ | Normal Cl + Normal HCO₃ | 1014 | 441 | 0.787 (0.458–1.354) | 0.388 |
| eICU | Septic shock | No | Normal Cl + High HCO₃ | Normal Cl + Normal HCO₃ | 20400 | 3466 | 1.101 (0.938–1.292) | 0.241 |
| eICU | Septic shock | Yes | Normal Cl + High HCO₃ | Normal Cl + Normal HCO₃ | 1014 | 441 | 1.644 (0.609–4.442) | 0.327 |
| eICU | Septic shock | No | High Cl + Low HCO₃ | Normal Cl + Normal HCO₃ | 20400 | 3466 | 1.113 (0.970–1.277) | 0.125 |
| eICU | Septic shock | Yes | High Cl + Low HCO₃ | Normal Cl + Normal HCO₃ | 1014 | 441 | 0.733 (0.431–1.246) | 0.251 |
| eICU | Septic shock | No | High Cl + Normal HCO₃ | Normal Cl + Normal HCO₃ | 20400 | 3466 | 0.754 (0.635–0.896) | 0.001 |
| eICU | Septic shock | Yes | High Cl + Normal HCO₃ | Normal Cl + Normal HCO₃ | 1014 | 441 | 0.758 (0.372–1.544) | 0.446 |
| eICU | Septic shock | No | High Cl + High HCO₃ | Normal Cl + Normal HCO₃ | 20400 | 3466 | 0.872 (0.664–1.145) | 0.325 |
| eICU | Septic shock | Yes | High Cl + High HCO₃ | Normal Cl + Normal HCO₃ | 1014 | 441 | 2.039 (0.566–7.354) | 0.276 |
| eICU | Sodium bicarbonate therapy | No | Low Cl + Low HCO₃ | Normal Cl + Normal HCO₃ | 18806 | 2906 | 1.663 (1.331–2.078) | <0.001 |
| eICU | Sodium bicarbonate therapy | Yes | Low Cl + Low HCO₃ | Normal Cl + Normal HCO₃ | 2608 | 1001 | 0.858 (0.535–1.376) | 0.525 |
| eICU | Sodium bicarbonate therapy | No | Low Cl + Normal HCO₃ | Normal Cl + Normal HCO₃ | 18806 | 2906 | 1.428 (1.142–1.786) | 0.002 |
| eICU | Sodium bicarbonate therapy | Yes | Low Cl + Normal HCO₃ | Normal Cl + Normal HCO₃ | 2608 | 1001 | 1.079 (0.516–2.257) | 0.839 |
| eICU | Sodium bicarbonate therapy | No | Low Cl + High HCO₃ | Normal Cl + Normal HCO₃ | 18806 | 2906 | 1.195 (0.971–1.471) | 0.093 |
| eICU | Sodium bicarbonate therapy | Yes | Low Cl + High HCO₃ | Normal Cl + Normal HCO₃ | 2608 | 1001 | 0.734 (0.256–2.103) | 0.564 |
| eICU | Sodium bicarbonate therapy | No | Normal Cl + Low HCO₃ | Normal Cl + Normal HCO₃ | 18806 | 2906 | 1.252 (1.080–1.452) | 0.003 |
| eICU | Sodium bicarbonate therapy | Yes | Normal Cl + Low HCO₃ | Normal Cl + Normal HCO₃ | 2608 | 1001 | 0.946 (0.633–1.414) | 0.788 |
| eICU | Sodium bicarbonate therapy | No | Normal Cl + High HCO₃ | Normal Cl + Normal HCO₃ | 18806 | 2906 | 1.089 (0.926–1.281) | 0.302 |
| eICU | Sodium bicarbonate therapy | Yes | Normal Cl + High HCO₃ | Normal Cl + Normal HCO₃ | 2608 | 1001 | 1.717 (0.847–3.478) | 0.134 |
| eICU | Sodium bicarbonate therapy | No | High Cl + Low HCO₃ | Normal Cl + Normal HCO₃ | 18806 | 2906 | 1.072 (0.928–1.239) | 0.346 |
| eICU | Sodium bicarbonate therapy | Yes | High Cl + Low HCO₃ | Normal Cl + Normal HCO₃ | 2608 | 1001 | 0.959 (0.646–1.424) | 0.836 |
| eICU | Sodium bicarbonate therapy | No | High Cl + Normal HCO₃ | Normal Cl + Normal HCO₃ | 18806 | 2906 | 0.752 (0.632–0.896) | 0.001 |
| eICU | Sodium bicarbonate therapy | Yes | High Cl + Normal HCO₃ | Normal Cl + Normal HCO₃ | 2608 | 1001 | 0.803 (0.446–1.448) | 0.466 |
| eICU | Sodium bicarbonate therapy | No | High Cl + High HCO₃ | Normal Cl + Normal HCO₃ | 18806 | 2906 | 0.922 (0.704–1.207) | 0.553 |
| eICU | Sodium bicarbonate therapy | Yes | High Cl + High HCO₃ | Normal Cl + Normal HCO₃ | 2608 | 1001 | 0.647 (0.178–2.349) | 0.508 |
| eICU | Vasoactive therapy | No | Low Cl + Low HCO₃ | Normal Cl + Normal HCO₃ | 15668 | 2112 | 1.583 (1.226–2.043) | <0.001 |
| eICU | Vasoactive therapy | Yes | Low Cl + Low HCO₃ | Normal Cl + Normal HCO₃ | 5746 | 1795 | 1.255 (0.928–1.697) | 0.141 |
| eICU | Vasoactive therapy | No | Low Cl + Normal HCO₃ | Normal Cl + Normal HCO₃ | 15668 | 2112 | 1.413 (1.090–1.833) | 0.009 |
| eICU | Vasoactive therapy | Yes | Low Cl + Normal HCO₃ | Normal Cl + Normal HCO₃ | 5746 | 1795 | 1.366 (0.939–1.987) | 0.103 |
| eICU | Vasoactive therapy | No | Low Cl + High HCO₃ | Normal Cl + Normal HCO₃ | 15668 | 2112 | 1.212 (0.958–1.535) | 0.109 |
| eICU | Vasoactive therapy | Yes | Low Cl + High HCO₃ | Normal Cl + Normal HCO₃ | 5746 | 1795 | 1.039 (0.695–1.553) | 0.852 |
| eICU | Vasoactive therapy | No | Normal Cl + Low HCO₃ | Normal Cl + Normal HCO₃ | 15668 | 2112 | 1.220 (1.029–1.447) | 0.022 |
| eICU | Vasoactive therapy | Yes | Normal Cl + Low HCO₃ | Normal Cl + Normal HCO₃ | 5746 | 1795 | 1.184 (0.947–1.482) | 0.138 |
| eICU | Vasoactive therapy | No | Normal Cl + High HCO₃ | Normal Cl + Normal HCO₃ | 15668 | 2112 | 1.094 (0.910–1.315) | 0.338 |
| eICU | Vasoactive therapy | Yes | Normal Cl + High HCO₃ | Normal Cl + Normal HCO₃ | 5746 | 1795 | 1.128 (0.831–1.531) | 0.440 |
| eICU | Vasoactive therapy | No | High Cl + Low HCO₃ | Normal Cl + Normal HCO₃ | 15668 | 2112 | 1.091 (0.925–1.287) | 0.301 |
| eICU | Vasoactive therapy | Yes | High Cl + Low HCO₃ | Normal Cl + Normal HCO₃ | 5746 | 1795 | 1.070 (0.860–1.331) | 0.545 |
| eICU | Vasoactive therapy | No | High Cl + Normal HCO₃ | Normal Cl + Normal HCO₃ | 15668 | 2112 | 0.704 (0.572–0.866) | <0.001 |
| eICU | Vasoactive therapy | Yes | High Cl + Normal HCO₃ | Normal Cl + Normal HCO₃ | 5746 | 1795 | 0.868 (0.654–1.153) | 0.328 |
| eICU | Vasoactive therapy | No | High Cl + High HCO₃ | Normal Cl + Normal HCO₃ | 15668 | 2112 | 0.903 (0.657–1.242) | 0.532 |
| eICU | Vasoactive therapy | Yes | High Cl + High HCO₃ | Normal Cl + Normal HCO₃ | 5746 | 1795 | 0.905 (0.566–1.446) | 0.675 |

*Note.* Normal chloride plus normal bicarbonate is the reference phenotype. The MIMIC-IV sodium-bicarbonate-treated high-chloride/high-bicarbonate contrast was not estimable because that sparse cell contained no deaths. Other estimates from this subgroup should be interpreted cautiously.

**Supplementary Table 9A.** Secondary outcome availability and event rates.

| **Cohort** | **Outcome** | **Total n** | **Non-missing n** | **Missing n** | **Events** | **Event rate (%)** |
| --- | --- | --- | --- | --- | --- | --- |
| MIMIC-IV | In-hospital mortality | 25842 | 25842 | 0 | 4216 | 16.31 |
| MIMIC-IV | 28-day mortality | 25842 | 25842 | 0 | 4746 | 18.37 |
| MIMIC-IV | 30-day mortality | 25842 | 25842 | 0 | 4835 | 18.71 |
| MIMIC-IV | 90-day mortality | 25842 | 25842 | 0 | 6344 | 24.55 |
| MIMIC-IV | 365-day mortality | 25842 | 25842 | 0 | 8274 | 32.02 |
| eICU | In-hospital mortality | 37454 | 37045 | 409 | 5741 | 15.50 |
| eICU | In-hospital death by day 28 after hospital admission | 37454 | 37045 | 409 | 5456 | 14.73 |
| eICU | In-hospital death by day 30 after hospital admission | 37454 | 37045 | 409 | 5491 | 14.82 |
| eICU | In-hospital death by day 90 after hospital admission | 37454 | 37045 | 409 | 5726 | 15.46 |
| eICU | In-hospital death by day 365 after hospital admission | 37454 | 37045 | 409 | 5739 | 15.49 |

*Note.* Because eICU does not provide complete post-discharge mortality follow-up, the day-specific endpoints represent in-hospital deaths occurring by the corresponding day after hospital admission and should not be interpreted as conventional all-cause mortality after hospital discharge. For eICU, records with missing in-hospital mortality status were treated as missing for all derived day-specific endpoints. This correction changed descriptive availability denominators only; the complete-case Model 3b samples and estimates in Supplementary Tables 9B–9C were unchanged because the affected records did not enter those inferential models.

**Supplementary Table 9B.** Secondary outcome overall phenotype tests.

| **Cohort** | **Outcome** | **n** | **Events** | **LRT chi-square** | **df** | **Overall P** | **Converged** |
| --- | --- | --- | --- | --- | --- | --- | --- |
| MIMIC-IV | In-hospital mortality | 13168 | 2370 | 18.587 | 8 | 0.017 | Yes |
| MIMIC-IV | 28-day mortality | 13168 | 2462 | 19.098 | 8 | 0.014 | Yes |
| MIMIC-IV | 90-day mortality | 13168 | 3145 | 26.243 | 8 | <0.001 | Yes |
| eICU | In-hospital mortality | 21414 | 3907 | 52.567 | 8 | <0.001 | Yes |
| eICU | In-hospital death by day 28 | 21414 | 3747 | 50.089 | 8 | <0.001 | Yes |
| eICU | In-hospital death by day 90 | 21414 | 3897 | 51.892 | 8 | <0.001 | Yes |

*Note.* Complete-case Model 3b was fitted separately for each database-specific outcome. Overall P values are likelihood-ratio tests of the joint phenotype. For eICU, records with missing in-hospital mortality status were also missing for the corrected day-specific endpoints; the Model 3b analytic samples and estimates were unchanged.

**Supplementary Table 9C.** Secondary outcome phenotype-specific odds ratios.

| **Cohort** | **Outcome** | **Contrast** | **Reference** | **n** | **Events** | **OR (95% CI)** | **P** | **Converged** |
| --- | --- | --- | --- | --- | --- | --- | --- | --- |
| MIMIC-IV | In-hospital mortality | Low Cl + Low HCO₃ | Normal Cl + Normal HCO₃ | 13168 | 2370 | 1.136 (0.885–1.460) | 0.317 | Yes |
| MIMIC-IV | In-hospital mortality | Low Cl + Normal HCO₃ | Normal Cl + Normal HCO₃ | 13168 | 2370 | 1.519 (1.159–1.990) | 0.002 | Yes |
| MIMIC-IV | In-hospital mortality | Low Cl + High HCO₃ | Normal Cl + Normal HCO₃ | 13168 | 2370 | 0.989 (0.742–1.319) | 0.940 | Yes |
| MIMIC-IV | In-hospital mortality | Normal Cl + Low HCO₃ | Normal Cl + Normal HCO₃ | 13168 | 2370 | 0.963 (0.813–1.141) | 0.666 | Yes |
| MIMIC-IV | In-hospital mortality | Normal Cl + High HCO₃ | Normal Cl + Normal HCO₃ | 13168 | 2370 | 0.925 (0.736–1.162) | 0.502 | Yes |
| MIMIC-IV | In-hospital mortality | High Cl + Low HCO₃ | Normal Cl + Normal HCO₃ | 13168 | 2370 | 0.920 (0.770–1.099) | 0.356 | Yes |
| MIMIC-IV | In-hospital mortality | High Cl + Normal HCO₃ | Normal Cl + Normal HCO₃ | 13168 | 2370 | 0.841 (0.673–1.049) | 0.125 | Yes |
| MIMIC-IV | In-hospital mortality | High Cl + High HCO₃ | Normal Cl + Normal HCO₃ | 13168 | 2370 | 1.189 (0.773–1.827) | 0.430 | Yes |
| MIMIC-IV | 28-day mortality | Low Cl + Low HCO₃ | Normal Cl + Normal HCO₃ | 13168 | 2462 | 1.300 (1.017–1.661) | 0.036 | Yes |
| MIMIC-IV | 28-day mortality | Low Cl + Normal HCO₃ | Normal Cl + Normal HCO₃ | 13168 | 2462 | 1.488 (1.141–1.942) | 0.003 | Yes |
| MIMIC-IV | 28-day mortality | Low Cl + High HCO₃ | Normal Cl + Normal HCO₃ | 13168 | 2462 | 0.987 (0.746–1.305) | 0.924 | Yes |
| MIMIC-IV | 28-day mortality | Normal Cl + Low HCO₃ | Normal Cl + Normal HCO₃ | 13168 | 2462 | 0.979 (0.829–1.156) | 0.799 | Yes |
| MIMIC-IV | 28-day mortality | Normal Cl + High HCO₃ | Normal Cl + Normal HCO₃ | 13168 | 2462 | 0.944 (0.756–1.178) | 0.611 | Yes |
| MIMIC-IV | 28-day mortality | High Cl + Low HCO₃ | Normal Cl + Normal HCO₃ | 13168 | 2462 | 0.931 (0.782–1.107) | 0.417 | Yes |
| MIMIC-IV | 28-day mortality | High Cl + Normal HCO₃ | Normal Cl + Normal HCO₃ | 13168 | 2462 | 0.920 (0.743–1.140) | 0.448 | Yes |
| MIMIC-IV | 28-day mortality | High Cl + High HCO₃ | Normal Cl + Normal HCO₃ | 13168 | 2462 | 1.158 (0.758–1.770) | 0.498 | Yes |
| MIMIC-IV | 90-day mortality | Low Cl + Low HCO₃ | Normal Cl + Normal HCO₃ | 13168 | 3145 | 1.263 (0.996–1.603) | 0.054 | Yes |
| MIMIC-IV | 90-day mortality | Low Cl + Normal HCO₃ | Normal Cl + Normal HCO₃ | 13168 | 3145 | 1.627 (1.264–2.093) | <0.001 | Yes |
| MIMIC-IV | 90-day mortality | Low Cl + High HCO₃ | Normal Cl + Normal HCO₃ | 13168 | 3145 | 1.083 (0.838–1.400) | 0.542 | Yes |
| MIMIC-IV | 90-day mortality | Normal Cl + Low HCO₃ | Normal Cl + Normal HCO₃ | 13168 | 3145 | 0.960 (0.823–1.121) | 0.607 | Yes |
| MIMIC-IV | 90-day mortality | Normal Cl + High HCO₃ | Normal Cl + Normal HCO₃ | 13168 | 3145 | 1.084 (0.888–1.324) | 0.427 | Yes |
| MIMIC-IV | 90-day mortality | High Cl + Low HCO₃ | Normal Cl + Normal HCO₃ | 13168 | 3145 | 0.907 (0.772–1.065) | 0.234 | Yes |
| MIMIC-IV | 90-day mortality | High Cl + Normal HCO₃ | Normal Cl + Normal HCO₃ | 13168 | 3145 | 0.969 (0.799–1.174) | 0.748 | Yes |
| MIMIC-IV | 90-day mortality | High Cl + High HCO₃ | Normal Cl + Normal HCO₃ | 13168 | 3145 | 1.115 (0.752–1.654) | 0.588 | Yes |
| eICU | In-hospital mortality | Low Cl + Low HCO₃ | Normal Cl + Normal HCO₃ | 21414 | 3907 | 1.426 (1.169–1.739) | <0.001 | Yes |
| eICU | In-hospital mortality | Low Cl + Normal HCO₃ | Normal Cl + Normal HCO₃ | 21414 | 3907 | 1.399 (1.129–1.733) | 0.002 | Yes |
| eICU | In-hospital mortality | Low Cl + High HCO₃ | Normal Cl + Normal HCO₃ | 21414 | 3907 | 1.166 (0.952–1.430) | 0.138 | Yes |
| eICU | In-hospital mortality | Normal Cl + Low HCO₃ | Normal Cl + Normal HCO₃ | 21414 | 3907 | 1.207 (1.053–1.384) | 0.007 | Yes |
| eICU | In-hospital mortality | Normal Cl + High HCO₃ | Normal Cl + Normal HCO₃ | 21414 | 3907 | 1.100 (0.940–1.288) | 0.233 | Yes |
| eICU | In-hospital mortality | High Cl + Low HCO₃ | Normal Cl + Normal HCO₃ | 21414 | 3907 | 1.084 (0.949–1.238) | 0.233 | Yes |
| eICU | In-hospital mortality | High Cl + Normal HCO₃ | Normal Cl + Normal HCO₃ | 21414 | 3907 | 0.758 (0.641–0.895) | 0.001 | Yes |
| eICU | In-hospital mortality | High Cl + High HCO₃ | Normal Cl + Normal HCO₃ | 21414 | 3907 | 0.903 (0.693–1.176) | 0.448 | Yes |
| eICU | In-hospital death by day 28 | Low Cl + Low HCO₃ | Normal Cl + Normal HCO₃ | 21414 | 3747 | 1.445 (1.182–1.767) | <0.001 | Yes |
| eICU | In-hospital death by day 28 | Low Cl + Normal HCO₃ | Normal Cl + Normal HCO₃ | 21414 | 3747 | 1.387 (1.114–1.727) | 0.003 | Yes |
| eICU | In-hospital death by day 28 | Low Cl + High HCO₃ | Normal Cl + Normal HCO₃ | 21414 | 3747 | 1.239 (1.009–1.523) | 0.041 | Yes |
| eICU | In-hospital death by day 28 | Normal Cl + Low HCO₃ | Normal Cl + Normal HCO₃ | 21414 | 3747 | 1.233 (1.073–1.418) | 0.003 | Yes |
| eICU | In-hospital death by day 28 | Normal Cl + High HCO₃ | Normal Cl + Normal HCO₃ | 21414 | 3747 | 1.113 (0.948–1.308) | 0.190 | Yes |
| eICU | In-hospital death by day 28 | High Cl + Low HCO₃ | Normal Cl + Normal HCO₃ | 21414 | 3747 | 1.122 (0.980–1.284) | 0.097 | Yes |
| eICU | In-hospital death by day 28 | High Cl + Normal HCO₃ | Normal Cl + Normal HCO₃ | 21414 | 3747 | 0.778 (0.656–0.923) | 0.004 | Yes |
| eICU | In-hospital death by day 28 | High Cl + High HCO₃ | Normal Cl + Normal HCO₃ | 21414 | 3747 | 0.933 (0.714–1.221) | 0.614 | Yes |
| eICU | In-hospital death by day 90 | Low Cl + Low HCO₃ | Normal Cl + Normal HCO₃ | 21414 | 3897 | 1.427 (1.170–1.740) | <0.001 | Yes |
| eICU | In-hospital death by day 90 | Low Cl + Normal HCO₃ | Normal Cl + Normal HCO₃ | 21414 | 3897 | 1.400 (1.130–1.734) | 0.002 | Yes |
| eICU | In-hospital death by day 90 | Low Cl + High HCO₃ | Normal Cl + Normal HCO₃ | 21414 | 3897 | 1.167 (0.952–1.430) | 0.137 | Yes |
| eICU | In-hospital death by day 90 | Normal Cl + Low HCO₃ | Normal Cl + Normal HCO₃ | 21414 | 3897 | 1.200 (1.047–1.376) | 0.009 | Yes |
| eICU | In-hospital death by day 90 | Normal Cl + High HCO₃ | Normal Cl + Normal HCO₃ | 21414 | 3897 | 1.084 (0.926–1.269) | 0.318 | Yes |
| eICU | In-hospital death by day 90 | High Cl + Low HCO₃ | Normal Cl + Normal HCO₃ | 21414 | 3897 | 1.079 (0.945–1.233) | 0.262 | Yes |
| eICU | In-hospital death by day 90 | High Cl + Normal HCO₃ | Normal Cl + Normal HCO₃ | 21414 | 3897 | 0.758 (0.641–0.896) | 0.001 | Yes |
| eICU | In-hospital death by day 90 | High Cl + High HCO₃ | Normal Cl + Normal HCO₃ | 21414 | 3897 | 0.903 (0.694–1.176) | 0.450 | Yes |

*Note.* The reference group is normal chloride plus normal bicarbonate. For eICU, the day-specific endpoints represent in-hospital death occurring by the corresponding day after hospital admission and should not be interpreted as conventional post-discharge all-cause mortality. The corrected missing-outcome handling did not change the complete-case Model 3b phenotype-specific estimates.

**Supplementary Table 10.** Direct Model 2b comparison of chloride, bicarbonate, continuous interaction, CHBI encodings, and the 3 × 3 joint phenotype, with paired bootstrap confidence intervals.

| **Cohort** | **Model** | **n** | **Events** | **Parameters** | **AUROC (95% CI)** | **ΔAUROC vs base (95% CI)** | **Brier score (95% CI)** | **ΔBrier vs base (95% CI)** | **Log loss (95% CI)** | **ΔLog loss vs base (95% CI)** | **AIC** | **BIC** | **Converged** |
| --- | --- | --- | --- | --- | --- | --- | --- | --- | --- | --- | --- | --- | --- |
| MIMIC-IV | Base Model 2b | 25842 | 4216 | 23 | 0.7344 (0.7274–0.7420) | 0.0000 (0.0000–0.0000) | 0.1242 (0.1214–0.1268) | 0.0000 (0.0000–0.0000) | 0.3953 (0.3880–0.4019) | 0.0000 (0.0000–0.0000) | 20475.0 | 20662.7 | Yes |
| MIMIC-IV | + chloride | 25842 | 4216 | 24 | 0.7368 (0.7299–0.7440) | 0.0023 (0.0006–0.0039) | 0.1239 (0.1211–0.1265) | -0.0003 (-0.0005–-0.0001) | 0.3941 (0.3869–0.4009) | -0.0011 (-0.0018–-0.0005) | 20418.4 | 20614.2 | Yes |
| MIMIC-IV | + bicarbonate | 25842 | 4216 | 24 | 0.7523 (0.7458–0.7602) | 0.0179 (0.0143–0.0216) | 0.1215 (0.1188–0.1241) | -0.0027 (-0.0033–-0.0021) | 0.3874 (0.3799–0.3942) | -0.0079 (-0.0096–-0.0062) | 20071.0 | 20266.8 | Yes |
| MIMIC-IV | + chloride + bicarbonate | 25842 | 4216 | 25 | 0.7579 (0.7517–0.7654) | 0.0235 (0.0191–0.0278) | 0.1206 (0.1180–0.1230) | -0.0036 (-0.0042–-0.0029) | 0.3844 (0.3771–0.3910) | -0.0108 (-0.0126–-0.0089) | 19918.6 | 20122.6 | Yes |
| MIMIC-IV | + chloride + bicarbonate + interaction | 25842 | 4216 | 26 | 0.7593 (0.7529–0.7667) | 0.0248 (0.0204–0.0295) | 0.1205 (0.1179–0.1230) | -0.0037 (-0.0044–-0.0030) | 0.3837 (0.3766–0.3903) | -0.0115 (-0.0134–-0.0095) | 19885.2 | 20097.3 | Yes |
| MIMIC-IV | + CHBI | 25842 | 4216 | 24 | 0.7380 (0.7305–0.7457) | 0.0036 (0.0019–0.0054) | 0.1237 (0.1210–0.1263) | -0.0005 (-0.0007–-0.0002) | 0.3939 (0.3865–0.4005) | -0.0014 (-0.0021–-0.0007) | 20406.5 | 20602.3 | Yes |
| MIMIC-IV | + absolute CHBI | 25842 | 4216 | 24 | 0.7381 (0.7312–0.7456) | 0.0037 (0.0017–0.0059) | 0.1238 (0.1211–0.1263) | -0.0004 (-0.0007–-0.0001) | 0.3935 (0.3864–0.4002) | -0.0017 (-0.0025–-0.0009) | 20387.9 | 20583.8 | Yes |
| MIMIC-IV | + CHBI quartiles | 25842 | 4216 | 26 | 0.7404 (0.7331–0.7482) | 0.0060 (0.0040–0.0083) | 0.1234 (0.1208–0.1259) | -0.0008 (-0.0011–-0.0004) | 0.3926 (0.3854–0.3991) | -0.0026 (-0.0035–-0.0018) | 20345.5 | 20557.6 | Yes |
| MIMIC-IV | + 3 × 3 joint phenotype | 25842 | 4216 | 31 | 0.7524 (0.7459–0.7600) | 0.0180 (0.0144–0.0218) | 0.1219 (0.1192–0.1244) | -0.0023 (-0.0029–-0.0018) | 0.3872 (0.3800–0.3937) | -0.0080 (-0.0095–-0.0066) | 20075.3 | 20328.3 | Yes |
| eICU | Base Model 2b | 37041 | 5741 | 20 | 0.6208 (0.6133–0.6284) | 0.0000 (0.0000–0.0000) | 0.1280 (0.1259–0.1306) | 0.0000 (0.0000–0.0000) | 0.4194 (0.4142–0.4259) | 0.0000 (0.0000–0.0000) | 31112.1 | 31282.5 | Yes |
| eICU | + chloride | 37041 | 5741 | 21 | 0.6208 (0.6133–0.6283) | -0.0000 (-0.0001–0.0001) | 0.1280 (0.1259–0.1306) | -0.0000 (-0.0000–0.0000) | 0.4194 (0.4142–0.4259) | -0.0000 (-0.0000–0.0000) | 31114.0 | 31292.9 | Yes |
| eICU | + bicarbonate | 37041 | 5741 | 21 | 0.6624 (0.6548–0.6692) | 0.0416 (0.0350–0.0481) | 0.1244 (0.1223–0.1269) | -0.0036 (-0.0041–-0.0031) | 0.4087 (0.4031–0.4152) | -0.0107 (-0.0125–-0.0091) | 30322.6 | 30501.5 | Yes |
| eICU | + chloride + bicarbonate | 37041 | 5741 | 22 | 0.6654 (0.6577–0.6725) | 0.0446 (0.0375–0.0512) | 0.1240 (0.1220–0.1266) | -0.0040 (-0.0045–-0.0034) | 0.4075 (0.4020–0.4142) | -0.0119 (-0.0136–-0.0102) | 30234.9 | 30422.3 | Yes |
| eICU | + chloride + bicarbonate + interaction | 37041 | 5741 | 23 | 0.6678 (0.6604–0.6747) | 0.0470 (0.0399–0.0538) | 0.1239 (0.1218–0.1264) | -0.0041 (-0.0047–-0.0036) | 0.4068 (0.4014–0.4134) | -0.0127 (-0.0145–-0.0109) | 30180.6 | 30376.6 | Yes |
| eICU | + CHBI | 37041 | 5741 | 21 | 0.6390 (0.6312–0.6465) | 0.0182 (0.0142–0.0226) | 0.1268 (0.1247–0.1294) | -0.0012 (-0.0014–-0.0009) | 0.4156 (0.4102–0.4219) | -0.0038 (-0.0048–-0.0030) | 30828.9 | 31007.8 | Yes |
| eICU | + absolute CHBI | 37041 | 5741 | 21 | 0.6289 (0.6213–0.6363) | 0.0082 (0.0052–0.0108) | 0.1275 (0.1254–0.1302) | -0.0005 (-0.0006–-0.0003) | 0.4177 (0.4123–0.4242) | -0.0017 (-0.0023–-0.0011) | 30987.6 | 31166.5 | Yes |
| eICU | + CHBI quartiles | 37041 | 5741 | 23 | 0.6426 (0.6345–0.6496) | 0.0218 (0.0172–0.0261) | 0.1267 (0.1246–0.1293) | -0.0013 (-0.0015–-0.0010) | 0.4148 (0.4091–0.4213) | -0.0046 (-0.0055–-0.0037) | 30774.5 | 30970.5 | Yes |
| eICU | + 3 × 3 joint phenotype | 37041 | 5741 | 28 | 0.6617 (0.6543–0.6682) | 0.0409 (0.0345–0.0470) | 0.1253 (0.1231–0.1278) | -0.0027 (-0.0031–-0.0023) | 0.4096 (0.4043–0.4160) | -0.0098 (-0.0113–-0.0083) | 30399.9 | 30638.4 | Yes |

*Note.* These exploratory comparisons describe apparent performance on a shared complete-case development sample. Confidence intervals were obtained with a fixed-prediction paired nonparametric bootstrap of 500 resamples: candidate models were fitted once in the original sample and were not refitted within bootstrap resamples. The intervals therefore quantify sampling variability of the observed prediction metrics, not optimism-corrected internal validation or expected performance in new patients. Delta values are relative to the cohort-specific Model 2b baseline.

**Supplementary Table 11.** Nested likelihood-ratio tests for the direct Model 2b representation comparison.

| **Cohort** | **Comparison** | **LR chi-square** | **df** | **P** |
| --- | --- | --- | --- | --- |
| MIMIC-IV | + chloride vs Base Model 2b | 58.605 | 1 | <0.001 |
| MIMIC-IV | + bicarbonate vs Base Model 2b | 405.972 | 1 | <0.001 |
| MIMIC-IV | + chloride + bicarbonate vs Base Model 2b | 560.396 | 2 | <0.001 |
| MIMIC-IV | + chloride + bicarbonate vs + chloride | 501.791 | 1 | <0.001 |
| MIMIC-IV | + chloride + bicarbonate vs + bicarbonate | 154.425 | 1 | <0.001 |
| MIMIC-IV | + chloride + bicarbonate + interaction vs + chloride + bicarbonate | 35.404 | 1 | <0.001 |
| MIMIC-IV | + CHBI vs Base Model 2b | 70.505 | 1 | <0.001 |
| MIMIC-IV | + absolute CHBI vs Base Model 2b | 89.043 | 1 | <0.001 |
| MIMIC-IV | + CHBI quartiles vs Base Model 2b | 135.504 | 3 | <0.001 |
| MIMIC-IV | + 3 × 3 joint phenotype vs Base Model 2b | 415.665 | 8 | <0.001 |
| eICU | + chloride vs Base Model 2b | 0.039 | 1 | 0.843 |
| eICU | + bicarbonate vs Base Model 2b | 791.473 | 1 | <0.001 |
| eICU | + chloride + bicarbonate vs Base Model 2b | 881.177 | 2 | <0.001 |
| eICU | + chloride + bicarbonate vs + chloride | 881.138 | 1 | <0.001 |
| eICU | + chloride + bicarbonate vs + bicarbonate | 89.704 | 1 | <0.001 |
| eICU | + chloride + bicarbonate + interaction vs + chloride + bicarbonate | 56.253 | 1 | <0.001 |
| eICU | + CHBI vs Base Model 2b | 285.148 | 1 | <0.001 |
| eICU | + absolute CHBI vs Base Model 2b | 126.504 | 1 | <0.001 |
| eICU | + CHBI quartiles vs Base Model 2b | 343.574 | 3 | <0.001 |
| eICU | + 3 × 3 joint phenotype vs Base Model 2b | 728.219 | 8 | <0.001 |

*Note.* Likelihood-ratio tests are reported only for nested model comparisons. P values are two-sided.

**Supplementary Table 12A.** Phenotype counts and crude mortality under primary and alternative threshold schemes in the complete-case Model 3b analysis samples.

| **Cohort** | **Threshold scheme** | **Normal chloride range** | **Normal bicarbonate range** | **Phenotype** | **n** | **Deaths** | **Mortality (%)** |
| --- | --- | --- | --- | --- | --- | --- | --- |
| MIMIC-IV | Primary | 98–107 mmol/L | 22–26 mmol/L | High Cl + High HCO₃ | 279 | 35 | 12.54 |
| MIMIC-IV | Primary | 98–107 mmol/L | 22–26 mmol/L | High Cl + Low HCO₃ | 2441 | 472 | 19.34 |
| MIMIC-IV | Primary | 98–107 mmol/L | 22–26 mmol/L | High Cl + Normal HCO₃ | 2657 | 165 | 6.21 |
| MIMIC-IV | Primary | 98–107 mmol/L | 22–26 mmol/L | Low Cl + High HCO₃ | 416 | 91 | 21.88 |
| MIMIC-IV | Primary | 98–107 mmol/L | 22–26 mmol/L | Low Cl + Low HCO₃ | 607 | 269 | 44.32 |
| MIMIC-IV | Primary | 98–107 mmol/L | 22–26 mmol/L | Low Cl + Normal HCO₃ | 419 | 132 | 31.50 |
| MIMIC-IV | Primary | 98–107 mmol/L | 22–26 mmol/L | Normal Cl + High HCO₃ | 996 | 148 | 14.86 |
| MIMIC-IV | Primary | 98–107 mmol/L | 22–26 mmol/L | Normal Cl + Low HCO₃ | 2419 | 673 | 27.82 |
| MIMIC-IV | Primary | 98–107 mmol/L | 22–26 mmol/L | Normal Cl + Normal HCO₃ | 2934 | 385 | 13.12 |
| MIMIC-IV | Alternative A | 97–108 mmol/L | 21–27 mmol/L | High Cl + High HCO₃ | 108 | 19 | 17.59 |
| MIMIC-IV | Alternative A | 97–108 mmol/L | 21–27 mmol/L | High Cl + Low HCO₃ | 1605 | 358 | 22.31 |
| MIMIC-IV | Alternative A | 97–108 mmol/L | 21–27 mmol/L | High Cl + Normal HCO₃ | 2760 | 178 | 6.45 |
| MIMIC-IV | Alternative A | 97–108 mmol/L | 21–27 mmol/L | Low Cl + High HCO₃ | 283 | 57 | 20.14 |
| MIMIC-IV | Alternative A | 97–108 mmol/L | 21–27 mmol/L | Low Cl + Low HCO₃ | 412 | 196 | 47.57 |
| MIMIC-IV | Alternative A | 97–108 mmol/L | 21–27 mmol/L | Low Cl + Normal HCO₃ | 435 | 149 | 34.25 |
| MIMIC-IV | Alternative A | 97–108 mmol/L | 21–27 mmol/L | Normal Cl + High HCO₃ | 786 | 129 | 16.41 |
| MIMIC-IV | Alternative A | 97–108 mmol/L | 21–27 mmol/L | Normal Cl + Low HCO₃ | 2231 | 671 | 30.08 |
| MIMIC-IV | Alternative A | 97–108 mmol/L | 21–27 mmol/L | Normal Cl + Normal HCO₃ | 4548 | 613 | 13.48 |
| MIMIC-IV | Alternative B | 98–106 mmol/L | 22–25 mmol/L | High Cl + High HCO₃ | 701 | 68 | 9.70 |
| MIMIC-IV | Alternative B | 98–106 mmol/L | 22–25 mmol/L | High Cl + Low HCO₃ | 2787 | 553 | 19.84 |
| MIMIC-IV | Alternative B | 98–106 mmol/L | 22–25 mmol/L | High Cl + Normal HCO₃ | 2788 | 170 | 6.10 |
| MIMIC-IV | Alternative B | 98–106 mmol/L | 22–25 mmol/L | Low Cl + High HCO₃ | 501 | 114 | 22.75 |
| MIMIC-IV | Alternative B | 98–106 mmol/L | 22–25 mmol/L | Low Cl + Low HCO₃ | 607 | 269 | 44.32 |
| MIMIC-IV | Alternative B | 98–106 mmol/L | 22–25 mmol/L | Low Cl + Normal HCO₃ | 334 | 109 | 32.63 |
| MIMIC-IV | Alternative B | 98–106 mmol/L | 22–25 mmol/L | Normal Cl + High HCO₃ | 1283 | 194 | 15.12 |
| MIMIC-IV | Alternative B | 98–106 mmol/L | 22–25 mmol/L | Normal Cl + Low HCO₃ | 2073 | 592 | 28.56 |
| MIMIC-IV | Alternative B | 98–106 mmol/L | 22–25 mmol/L | Normal Cl + Normal HCO₃ | 2094 | 301 | 14.37 |
| eICU | Primary | 98–107 mmol/L | 22–26 mmol/L | High Cl + High HCO₃ | 600 | 95 | 15.83 |
| eICU | Primary | 98–107 mmol/L | 22–26 mmol/L | High Cl + Low HCO₃ | 4484 | 1016 | 22.66 |
| eICU | Primary | 98–107 mmol/L | 22–26 mmol/L | High Cl + Normal HCO₃ | 2493 | 298 | 11.95 |
| eICU | Primary | 98–107 mmol/L | 22–26 mmol/L | Low Cl + High HCO₃ | 1128 | 169 | 14.98 |
| eICU | Primary | 98–107 mmol/L | 22–26 mmol/L | Low Cl + Low HCO₃ | 1038 | 307 | 29.58 |
| eICU | Primary | 98–107 mmol/L | 22–26 mmol/L | Low Cl + Normal HCO₃ | 879 | 170 | 19.34 |
| eICU | Primary | 98–107 mmol/L | 22–26 mmol/L | Normal Cl + High HCO₃ | 2654 | 357 | 13.45 |
| eICU | Primary | 98–107 mmol/L | 22–26 mmol/L | Normal Cl + Low HCO₃ | 3907 | 937 | 23.98 |
| eICU | Primary | 98–107 mmol/L | 22–26 mmol/L | Normal Cl + Normal HCO₃ | 4231 | 558 | 13.19 |
| eICU | Alternative A | 97–108 mmol/L | 21–27 mmol/L | High Cl + High HCO₃ | 285 | 52 | 18.25 |
| eICU | Alternative A | 97–108 mmol/L | 21–27 mmol/L | High Cl + Low HCO₃ | 3364 | 817 | 24.29 |
| eICU | Alternative A | 97–108 mmol/L | 21–27 mmol/L | High Cl + Normal HCO₃ | 2686 | 351 | 13.07 |
| eICU | Alternative A | 97–108 mmol/L | 21–27 mmol/L | Low Cl + High HCO₃ | 794 | 129 | 16.25 |
| eICU | Alternative A | 97–108 mmol/L | 21–27 mmol/L | Low Cl + Low HCO₃ | 706 | 212 | 30.03 |
| eICU | Alternative A | 97–108 mmol/L | 21–27 mmol/L | Low Cl + Normal HCO₃ | 939 | 181 | 19.28 |
| eICU | Alternative A | 97–108 mmol/L | 21–27 mmol/L | Normal Cl + High HCO₃ | 2266 | 315 | 13.90 |
| eICU | Alternative A | 97–108 mmol/L | 21–27 mmol/L | Normal Cl + Low HCO₃ | 3732 | 966 | 25.88 |
| eICU | Alternative A | 97–108 mmol/L | 21–27 mmol/L | Normal Cl + Normal HCO₃ | 6642 | 884 | 13.31 |
| eICU | Alternative B | 98–106 mmol/L | 22–25 mmol/L | High Cl + High HCO₃ | 1133 | 160 | 14.12 |
| eICU | Alternative B | 98–106 mmol/L | 22–25 mmol/L | High Cl + Low HCO₃ | 5050 | 1137 | 22.51 |
| eICU | Alternative B | 98–106 mmol/L | 22–25 mmol/L | High Cl + Normal HCO₃ | 2673 | 327 | 12.23 |
| eICU | Alternative B | 98–106 mmol/L | 22–25 mmol/L | Low Cl + High HCO₃ | 1279 | 195 | 15.25 |
| eICU | Alternative B | 98–106 mmol/L | 22–25 mmol/L | Low Cl + Low HCO₃ | 1038 | 307 | 29.58 |
| eICU | Alternative B | 98–106 mmol/L | 22–25 mmol/L | Low Cl + Normal HCO₃ | 728 | 144 | 19.78 |
| eICU | Alternative B | 98–106 mmol/L | 22–25 mmol/L | Normal Cl + High HCO₃ | 3152 | 401 | 12.72 |
| eICU | Alternative B | 98–106 mmol/L | 22–25 mmol/L | Normal Cl + Low HCO₃ | 3341 | 816 | 24.42 |
| eICU | Alternative B | 98–106 mmol/L | 22–25 mmol/L | Normal Cl + Normal HCO₃ | 3020 | 420 | 13.91 |

*Note.* Counts and crude mortality percentages are based on the cohort-specific complete-case Model 3b samples (MIMIC-IV, n = 13,168; eICU, n = 21,414), not the full descriptive cohorts. Primary normal ranges were chloride 98–107 mmol/L and bicarbonate 22–26 mmol/L.

**Supplementary Table 12B.** Complete-case Model 3b phenotype associations under primary and alternative chloride-bicarbonate threshold schemes.

| **Cohort** | **Scheme** | **Cl cutpoints** | **HCO₃ cutpoints** | **n** | **Events** | **Global LR chi-square** | **Global P** | **Phenotype** | **OR (95% CI)** | **P** |
| --- | --- | --- | --- | --- | --- | --- | --- | --- | --- | --- |
| MIMIC-IV | Primary | 98–107 | 22–26 | 13168 | 2370 | 18.587 | 0.017 | Low Cl + Low HCO₃ | 1.136 (0.885–1.460) | 0.317 |
| MIMIC-IV | Primary | 98–107 | 22–26 | 13168 | 2370 | 18.587 | 0.017 | Low Cl + Normal HCO₃ | 1.519 (1.159–1.990) | 0.002 |
| MIMIC-IV | Primary | 98–107 | 22–26 | 13168 | 2370 | 18.587 | 0.017 | Low Cl + High HCO₃ | 0.989 (0.742–1.319) | 0.940 |
| MIMIC-IV | Primary | 98–107 | 22–26 | 13168 | 2370 | 18.587 | 0.017 | Normal Cl + Low HCO₃ | 0.963 (0.813–1.141) | 0.666 |
| MIMIC-IV | Primary | 98–107 | 22–26 | 13168 | 2370 | 18.587 | 0.017 | Normal Cl + High HCO₃ | 0.925 (0.736–1.162) | 0.502 |
| MIMIC-IV | Primary | 98–107 | 22–26 | 13168 | 2370 | 18.587 | 0.017 | High Cl + Low HCO₃ | 0.920 (0.770–1.099) | 0.356 |
| MIMIC-IV | Primary | 98–107 | 22–26 | 13168 | 2370 | 18.587 | 0.017 | High Cl + Normal HCO₃ | 0.841 (0.673–1.049) | 0.125 |
| MIMIC-IV | Primary | 98–107 | 22–26 | 13168 | 2370 | 18.587 | 0.017 | High Cl + High HCO₃ | 1.189 (0.773–1.827) | 0.430 |
| MIMIC-IV | Alternative A | 97–108 | 21–27 | 13168 | 2370 | 21.713 | 0.005 | Low Cl + Low HCO₃ | 1.082 (0.817–1.433) | 0.583 |
| MIMIC-IV | Alternative A | 97–108 | 21–27 | 13168 | 2370 | 21.713 | 0.005 | Low Cl + Normal HCO₃ | 1.663 (1.294–2.137) | <0.001 |
| MIMIC-IV | Alternative A | 97–108 | 21–27 | 13168 | 2370 | 21.713 | 0.005 | Low Cl + High HCO₃ | 0.928 (0.664–1.296) | 0.660 |
| MIMIC-IV | Alternative A | 97–108 | 21–27 | 13168 | 2370 | 21.713 | 0.005 | Normal Cl + Low HCO₃ | 0.992 (0.848–1.160) | 0.920 |
| MIMIC-IV | Alternative A | 97–108 | 21–27 | 13168 | 2370 | 21.713 | 0.005 | Normal Cl + High HCO₃ | 0.978 (0.776–1.232) | 0.848 |
| MIMIC-IV | Alternative A | 97–108 | 21–27 | 13168 | 2370 | 21.713 | 0.005 | High Cl + Low HCO₃ | 0.956 (0.799–1.143) | 0.621 |
| MIMIC-IV | Alternative A | 97–108 | 21–27 | 13168 | 2370 | 21.713 | 0.005 | High Cl + Normal HCO₃ | 0.873 (0.713–1.069) | 0.189 |
| MIMIC-IV | Alternative A | 97–108 | 21–27 | 13168 | 2370 | 21.713 | 0.005 | High Cl + High HCO₃ | 1.343 (0.744–2.423) | 0.327 |
| MIMIC-IV | Alternative B | 98–106 | 22–25 | 13168 | 2370 | 21.798 | 0.005 | Low Cl + Low HCO₃ | 1.107 (0.855–1.433) | 0.443 |
| MIMIC-IV | Alternative B | 98–106 | 22–25 | 13168 | 2370 | 21.798 | 0.005 | Low Cl + Normal HCO₃ | 1.591 (1.176–2.153) | 0.003 |
| MIMIC-IV | Alternative B | 98–106 | 22–25 | 13168 | 2370 | 21.798 | 0.005 | Low Cl + High HCO₃ | 0.986 (0.750–1.295) | 0.917 |
| MIMIC-IV | Alternative B | 98–106 | 22–25 | 13168 | 2370 | 21.798 | 0.005 | Normal Cl + Low HCO₃ | 0.948 (0.787–1.142) | 0.574 |
| MIMIC-IV | Alternative B | 98–106 | 22–25 | 13168 | 2370 | 21.798 | 0.005 | Normal Cl + High HCO₃ | 0.947 (0.760–1.180) | 0.627 |
| MIMIC-IV | Alternative B | 98–106 | 22–25 | 13168 | 2370 | 21.798 | 0.005 | High Cl + Low HCO₃ | 0.885 (0.737–1.064) | 0.193 |
| MIMIC-IV | Alternative B | 98–106 | 22–25 | 13168 | 2370 | 21.798 | 0.005 | High Cl + Normal HCO₃ | 0.773 (0.615–0.971) | 0.027 |
| MIMIC-IV | Alternative B | 98–106 | 22–25 | 13168 | 2370 | 21.798 | 0.005 | High Cl + High HCO₃ | 0.969 (0.702–1.337) | 0.848 |
| eICU | Primary | 98–107 | 22–26 | 21414 | 3907 | 52.567 | <0.001 | Low Cl + Low HCO₃ | 1.426 (1.169–1.739) | <0.001 |
| eICU | Primary | 98–107 | 22–26 | 21414 | 3907 | 52.567 | <0.001 | Low Cl + Normal HCO₃ | 1.399 (1.129–1.733) | 0.002 |
| eICU | Primary | 98–107 | 22–26 | 21414 | 3907 | 52.567 | <0.001 | Low Cl + High HCO₃ | 1.166 (0.952–1.430) | 0.138 |
| eICU | Primary | 98–107 | 22–26 | 21414 | 3907 | 52.567 | <0.001 | Normal Cl + Low HCO₃ | 1.207 (1.053–1.384) | 0.007 |
| eICU | Primary | 98–107 | 22–26 | 21414 | 3907 | 52.567 | <0.001 | Normal Cl + High HCO₃ | 1.100 (0.940–1.288) | 0.233 |
| eICU | Primary | 98–107 | 22–26 | 21414 | 3907 | 52.567 | <0.001 | High Cl + Low HCO₃ | 1.084 (0.949–1.238) | 0.233 |
| eICU | Primary | 98–107 | 22–26 | 21414 | 3907 | 52.567 | <0.001 | High Cl + Normal HCO₃ | 0.758 (0.641–0.895) | 0.001 |
| eICU | Primary | 98–107 | 22–26 | 21414 | 3907 | 52.567 | <0.001 | High Cl + High HCO₃ | 0.903 (0.693–1.176) | 0.448 |
| eICU | Alternative A | 97–108 | 21–27 | 21414 | 3907 | 46.442 | <0.001 | Low Cl + Low HCO₃ | 1.307 (1.047–1.632) | 0.018 |
| eICU | Alternative A | 97–108 | 21–27 | 21414 | 3907 | 46.442 | <0.001 | Low Cl + Normal HCO₃ | 1.403 (1.148–1.714) | <0.001 |
| eICU | Alternative A | 97–108 | 21–27 | 21414 | 3907 | 46.442 | <0.001 | Low Cl + High HCO₃ | 1.272 (1.021–1.586) | 0.032 |
| eICU | Alternative A | 97–108 | 21–27 | 21414 | 3907 | 46.442 | <0.001 | Normal Cl + Low HCO₃ | 1.268 (1.120–1.435) | <0.001 |
| eICU | Alternative A | 97–108 | 21–27 | 21414 | 3907 | 46.442 | <0.001 | Normal Cl + High HCO₃ | 1.137 (0.976–1.325) | 0.099 |
| eICU | Alternative A | 97–108 | 21–27 | 21414 | 3907 | 46.442 | <0.001 | High Cl + Low HCO₃ | 1.134 (0.999–1.288) | 0.052 |
| eICU | Alternative A | 97–108 | 21–27 | 21414 | 3907 | 46.442 | <0.001 | High Cl + Normal HCO₃ | 0.817 (0.705–0.948) | 0.008 |
| eICU | Alternative A | 97–108 | 21–27 | 21414 | 3907 | 46.442 | <0.001 | High Cl + High HCO₃ | 0.975 (0.692–1.375) | 0.886 |
| eICU | Alternative B | 98–106 | 22–25 | 21414 | 3907 | 46.514 | <0.001 | Low Cl + Low HCO₃ | 1.401 (1.139–1.722) | 0.001 |
| eICU | Alternative B | 98–106 | 22–25 | 21414 | 3907 | 46.514 | <0.001 | Low Cl + Normal HCO₃ | 1.387 (1.095–1.756) | 0.007 |
| eICU | Alternative B | 98–106 | 22–25 | 21414 | 3907 | 46.514 | <0.001 | Low Cl + High HCO₃ | 1.160 (0.947–1.420) | 0.152 |
| eICU | Alternative B | 98–106 | 22–25 | 21414 | 3907 | 46.514 | <0.001 | Normal Cl + Low HCO₃ | 1.189 (1.020–1.386) | 0.027 |
| eICU | Alternative B | 98–106 | 22–25 | 21414 | 3907 | 46.514 | <0.001 | Normal Cl + High HCO₃ | 1.038 (0.882–1.221) | 0.654 |
| eICU | Alternative B | 98–106 | 22–25 | 21414 | 3907 | 46.514 | <0.001 | High Cl + Low HCO₃ | 1.075 (0.932–1.240) | 0.319 |
| eICU | Alternative B | 98–106 | 22–25 | 21414 | 3907 | 46.514 | <0.001 | High Cl + Normal HCO₃ | 0.781 (0.657–0.929) | 0.005 |
| eICU | Alternative B | 98–106 | 22–25 | 21414 | 3907 | 46.514 | <0.001 | High Cl + High HCO₃ | 0.880 (0.708–1.094) | 0.250 |

*Note.* Complete-case Model 3b was used. The reference group is normal chloride plus normal bicarbonate under the corresponding threshold scheme. Global P values are likelihood-ratio tests. All eight nonreference cell-specific ORs are reported for each threshold scheme.

**Supplementary Table 13.** Simultaneous chloride and bicarbonate spline nonlinearity tests.

| **Cohort** | **Test** | **n** | **Events** | **LR chi-square** | **df** | **P** |
| --- | --- | --- | --- | --- | --- | --- |
| MIMIC-IV | Joint nonlinearity: chloride and bicarbonate | 13168 | 2370 | 24.264 | 6 | <0.001 |
| MIMIC-IV | Chloride nonlinearity conditional on linear bicarbonate | 13168 | 2370 | 8.259 | 3 | 0.041 |
| MIMIC-IV | Bicarbonate nonlinearity conditional on linear chloride | 13168 | 2370 | 15.275 | 3 | 0.002 |
| eICU | Joint nonlinearity: chloride and bicarbonate | 21414 | 3907 | 24.260 | 6 | <0.001 |
| eICU | Chloride nonlinearity conditional on linear bicarbonate | 21414 | 3907 | 1.936 | 3 | 0.586 |
| eICU | Bicarbonate nonlinearity conditional on linear chloride | 21414 | 3907 | 23.242 | 3 | <0.001 |

*Note.* Chloride and bicarbonate were modeled simultaneously using natural cubic splines with 4 degrees of freedom per exposure. Likelihood-ratio tests assess joint or exposure-specific departures from linearity.

**Supplementary Table 14.** MIMIC-IV-to-eICU external validation of alternative chloride-bicarbonate encodings.

| **Model** | **Training n** | **Training events** | **Validation n** | **Validation events** | **AUROC (95% CI)** | **ΔAUROC vs base (95% CI)** | **Brier score (95% CI)** | **ΔBrier vs base (95% CI)** | **Log loss (95% CI)** | **ΔLog loss vs base (95% CI)** | **Calibration intercept offset** | **Calibration slope** |
| --- | --- | --- | --- | --- | --- | --- | --- | --- | --- | --- | --- | --- |
| Portable base | 14739 | 2719 | 23824 | 4305 | 0.7886 (0.7815–0.7952) | 0.0000 (0.0000–0.0000) | 0.1234 (0.1209–0.1258) | 0.0000 (0.0000–0.0000) | 0.3938 (0.3872–0.4004) | 0.0000 (0.0000–0.0000) | -0.200 | 0.846 |
| + chloride | 14739 | 2719 | 23824 | 4305 | 0.7883 (0.7813–0.7953) | -0.0003 (-0.0019–0.0013) | 0.1228 (0.1202–0.1253) | -0.0006 (-0.0010–-0.0002) | 0.3925 (0.3857–0.3994) | -0.0012 (-0.0024–-0.0002) | -0.156 | 0.857 |
| + bicarbonate | 14739 | 2719 | 23824 | 4305 | 0.7887 (0.7816–0.7953) | 0.0000 (0.0000–0.0001) | 0.1234 (0.1209–0.1258) | -0.0000 (-0.0000–-0.0000) | 0.3937 (0.3872–0.4003) | -0.0001 (-0.0001–-0.0001) | -0.199 | 0.847 |
| + chloride + bicarbonate | 14739 | 2719 | 23824 | 4305 | 0.7898 (0.7827–0.7968) | 0.0012 (-0.0005–0.0028) | 0.1222 (0.1196–0.1247) | -0.0012 (-0.0016–-0.0008) | 0.3909 (0.3841–0.3977) | -0.0029 (-0.0041–-0.0017) | -0.135 | 0.867 |
| + chloride + bicarbonate + interaction | 14739 | 2719 | 23824 | 4305 | 0.7900 (0.7831–0.7970) | 0.0014 (-0.0005–0.0035) | 0.1221 (0.1196–0.1245) | -0.0013 (-0.0018–-0.0009) | 0.3904 (0.3836–0.3973) | -0.0033 (-0.0047–-0.0021) | -0.141 | 0.864 |
| + CHBI | 14739 | 2719 | 23824 | 4305 | 0.7870 (0.7803–0.7938) | -0.0016 (-0.0027–-0.0005) | 0.1238 (0.1212–0.1262) | 0.0004 (0.0001–0.0006) | 0.3950 (0.3882–0.4017) | 0.0012 (0.0005–0.0019) | -0.200 | 0.841 |
| + absolute CHBI | 14739 | 2719 | 23824 | 4305 | 0.7897 (0.7829–0.7965) | 0.0011 (0.0001–0.0022) | 0.1233 (0.1208–0.1257) | -0.0001 (-0.0004–0.0001) | 0.3931 (0.3865–0.3997) | -0.0007 (-0.0013–-0.0000) | -0.210 | 0.850 |
| + CHBI quartiles | 14739 | 2719 | 23824 | 4305 | 0.7877 (0.7809–0.7947) | -0.0009 (-0.0024–0.0006) | 0.1236 (0.1211–0.1260) | 0.0002 (-0.0001–0.0005) | 0.3942 (0.3877–0.4009) | 0.0005 (-0.0005–0.0014) | -0.206 | 0.847 |
| + 3 × 3 joint phenotype | 14739 | 2719 | 23824 | 4305 | 0.7896 (0.7825–0.7965) | 0.0010 (-0.0013–0.0031) | 0.1223 (0.1198–0.1248) | -0.0011 (-0.0016–-0.0006) | 0.3909 (0.3842–0.3977) | -0.0028 (-0.0043–-0.0015) | -0.159 | 0.870 |

*Note.* Coefficients were estimated in MIMIC-IV v3.1 and applied to eICU v2.0 without refitting. Continuous terms were standardized using MIMIC-IV training parameters. Confidence intervals and delta metrics were obtained from 500 paired bootstrap resamples of the eICU validation cohort. Calibration intercept offset represents calibration-in-the-large and was estimated with the logit of predicted probability entered as an offset (slope fixed at 1); calibration slope was estimated separately from a recalibration model with both intercept and slope free. Data-driven threshold comparisons and NRI/IDI analyses are provided in Supplementary Tables 17 and 19, respectively.

**Supplementary Table 15A.** Strict pre-exposure phenotype model summary.

| **Cohort** | **Model** | **n** | **Events** | **Global LR chi-square** | **Global P** |
| --- | --- | --- | --- | --- | --- |
| MIMIC-IV | Strict pre-exposure complete-case model | 8684 | 1034 | 24.951 | 0.002 |
| eICU | Strict pre-exposure complete-case model | 21194 | 3747 | 113.307 | <0.001 |

Note. The strict pre-exposure complete-case model included only covariate information available no later than completion of the paired chloride-bicarbonate exposure. Global P values are likelihood-ratio tests. The complete database-specific covariate and time-anchor definitions are provided in the Supplementary analytical methods.

**Supplementary Table 15B.** Strict pre-exposure phenotype-specific associations.

| **Cohort** | **n** | **Events** | **Global P** | **Phenotype** | **OR (95% CI)** | **P** |
| --- | --- | --- | --- | --- | --- | --- |
| MIMIC-IV | 8684 | 1034 | 0.002 | Low Cl + Low HCO₃ | 1.700 (1.143–2.528) | 0.009 |
| MIMIC-IV | 8684 | 1034 | 0.002 | Low Cl + Normal HCO₃ | 1.522 (0.986–2.349) | 0.058 |
| MIMIC-IV | 8684 | 1034 | 0.002 | Low Cl + High HCO₃ | 0.978 (0.636–1.504) | 0.918 |
| MIMIC-IV | 8684 | 1034 | 0.002 | Normal Cl + Low HCO₃ | 1.347 (1.057–1.717) | 0.016 |
| MIMIC-IV | 8684 | 1034 | 0.002 | Normal Cl + High HCO₃ | 1.113 (0.806–1.538) | 0.516 |
| MIMIC-IV | 8684 | 1034 | 0.002 | High Cl + Low HCO₃ | 1.396 (1.095–1.780) | 0.007 |
| MIMIC-IV | 8684 | 1034 | 0.002 | High Cl + Normal HCO₃ | 0.787 (0.580–1.069) | 0.126 |
| MIMIC-IV | 8684 | 1034 | 0.002 | High Cl + High HCO₃ | 1.055 (0.564–1.973) | 0.867 |
| eICU | 21194 | 3747 | <0.001 | Low Cl + Low HCO₃ | 1.787 (1.480–2.158) | <0.001 |
| eICU | 21194 | 3747 | <0.001 | Low Cl + Normal HCO₃ | 1.463 (1.191–1.798) | <0.001 |
| eICU | 21194 | 3747 | <0.001 | Low Cl + High HCO₃ | 1.226 (1.003–1.498) | 0.046 |
| eICU | 21194 | 3747 | <0.001 | Normal Cl + Low HCO₃ | 1.543 (1.359–1.753) | <0.001 |
| eICU | 21194 | 3747 | <0.001 | Normal Cl + High HCO₃ | 1.158 (0.997–1.345) | 0.056 |
| eICU | 21194 | 3747 | <0.001 | High Cl + Low HCO₃ | 1.467 (1.297–1.659) | <0.001 |
| eICU | 21194 | 3747 | <0.001 | High Cl + Normal HCO₃ | 0.857 (0.733–1.003) | 0.055 |
| eICU | 21194 | 3747 | <0.001 | High Cl + High HCO₃ | 1.094 (0.853–1.403) | 0.478 |

Note. The strict pre-exposure complete-case model used normal chloride plus normal bicarbonate as the reference group. Only covariate information available no later than completion of the paired exposure was eligible; see the Supplementary analytical methods for the full database-specific covariate and time-anchor definitions.

Supplementary Table 16. Categorical incremental-information and chloride-by-bicarbonate interaction tests.

| **Cohort** | **Comparison** | **n** | **Events** | **LR χ²** | **df** | **P** |
| --- | --- | --- | --- | --- | --- | --- |
| MIMIC-IV | Cl categories beyond HCO₃ | 13,168 | 2,370 | 9.76 | 2 | 0.008 |
| MIMIC-IV | HCO₃ categories beyond Cl | 13,168 | 2,370 | 1.12 | 2 | 0.571 |
| MIMIC-IV | Cl × HCO₃ interaction beyond additive main effects | 13,168 | 2,370 | 7.90 | 4 | 0.095 |
| eICU | Cl categories beyond HCO₃ | 21,414 | 3,907 | 29.97 | 2 | <0.001 |
| eICU | HCO₃ categories beyond Cl | 21,414 | 3,907 | 20.79 | 2 | <0.001 |
| eICU | Cl × HCO₃ interaction beyond additive main effects | 21,414 | 3,907 | 7.57 | 4 | 0.109 |

Note. All tests used the cohort-specific complete-case Model 3b adjustment set. The 2-df likelihood-ratio tests compare an additive categorical main-effect model with a model containing only the other biomarker category plus covariates. The 4-df interaction test compares additive chloride- and bicarbonate-category main effects with the corresponding full interaction model. Statistical significance of incremental main effects does not imply clinically meaningful improvement in prediction, and a nonsignificant interaction does not imply that the biomarkers are individually unassociated with outcome. For the incremental main-effect comparisons, apparent ΔAUROC/ΔBrier were +0.00024/−0.00013 for chloride beyond bicarbonate and +0.00007/−0.00003 for bicarbonate beyond chloride in MIMIC-IV; corresponding eICU values were +0.00101/−0.00020 and +0.00052/−0.00015. These are development-sample metrics and were not optimism corrected.

**Supplementary Table 17A.** External performance of the prespecified clinical and MIMIC-derived CART 3 × 3 representations.

| **Model** | **Training n** | **Validation n** | **AUROC** | **Brier** | **Log loss** | **Calibration slope** |
| --- | --- | --- | --- | --- | --- | --- |
| Portable base | 14,739 | 23,824 | 0.7886 | 0.1234 | 0.3938 | 0.846 |
| Clinical 3 × 3 | 14,739 | 23,824 | 0.7896 | 0.1223 | 0.3909 | 0.870 |
| CART-locked 3 × 3 | 14,739 | 23,824 | 0.7905 | 0.1220 | 0.3898 | 0.866 |

*Note.* CART cut points were derived from the full MIMIC-IV paired-exposure cohort (n=25,842; 4,216 deaths). Portable prediction models were subsequently fitted in the complete-case MIMIC-IV transport cohort (n=14,739; 2,719 deaths) and applied to the same eICU validation cohort (n=23,824; 4,305 deaths) without refitting. The clinical cut points were chloride 98 and 107 mmol/L and bicarbonate 22 and 26 mmol/L. Constrained MIMIC-IV CART identified chloride split points of 97.5 and 101.5 mmol/L and bicarbonate split points of 17.5 and 21.5 mmol/L; these split points were locked before eICU application. Across 500 bootstrap derivation resamples, the lower chloride split was 96.5, 97.5, or 98.5 mmol/L in 78.2% of resamples and the 101.5 mmol/L upper chloride split recurred in 63.0%; bicarbonate splits at 17.5 and 21.5 mmol/L recurred in 66.4% and 68.0%, respectively. This moderate rather than absolute stability supports treating the CART thresholds as sample-dependent prognostic sensitivity thresholds. Lower Brier score and log loss indicate better overall predictive accuracy.

**Supplementary Table 17B.** Paired external-validation comparison of CART-locked versus clinical 3 × 3 representations.

| **Metric** | **CART minus clinical** | **95% CI** |
| --- | --- | --- |
| ΔAUROC | 0.00093 | -0.00099 to 0.00288 |
| ΔBrier | -0.00036 | -0.00075 to 0.00006 |
| Δlog loss | -0.00113 | -0.00245 to 0.00004 |

*Note.* Differences were calculated in the fixed eICU validation cohort using 500 paired case-resampling bootstrap replicates. Positive ΔAUROC favors CART; negative ΔBrier or Δlog loss favors CART. Confidence intervals crossing zero indicate no clear performance difference. The analysis compares prognostic encodings and does not define treatment thresholds.

**Supplementary Table 17C.** Complete cell-specific Model 3b associations under the locked MIMIC-derived CART 3 × 3 threshold scheme.

| **Phenotype** | **MIMIC-IV OR (95% CI)** | **P** | **eICU OR (95% CI)** | **P** |
| --- | --- | --- | --- | --- |
| Lower Cl + Lower HCO₃ | 0.947 (0.676–1.327) | 0.752 | 1.510 (1.152–1.979) | 0.003 |
| Lower Cl + Middle HCO₃ | 1.522 (1.128–2.053) | 0.006 | 1.786 (1.415–2.254) | <0.001 |
| Lower Cl + Upper HCO₃ | 1.342 (1.091–1.651) | 0.005 | 1.404 (1.209–1.631) | <0.001 |
| Middle Cl + Lower HCO₃ | 1.198 (0.848–1.692) | 0.305 | 2.015 (1.549–2.622) | <0.001 |
| Middle Cl + Middle HCO₃ | 1.149 (0.874–1.510) | 0.320 | 1.463 (1.160–1.845) | 0.001 |
| Middle Cl + Upper HCO₃ | 1.129 (0.924–1.379) | 0.235 | 1.225 (1.057–1.421) | 0.007 |
| Upper Cl + Lower HCO₃ | 1.000 (0.826–1.210) | 0.998 | 1.552 (1.360–1.770) | <0.001 |
| Upper Cl + Middle HCO₃ | 0.977 (0.834–1.144) | 0.770 | 1.035 (0.919–1.166) | 0.571 |
| Upper Cl + Upper HCO₃ | 1.000 (reference) | — | 1.000 (reference) | — |

*Note.* CART split points were derived in MIMIC-IV and locked before eICU application: chloride 97.5 and 101.5 mmol/L and bicarbonate 17.5 and 21.5 mmol/L. Thus, lower/middle/upper categories correspond to ≤97.5, >97.5–101.5, and >101.5 mmol/L for chloride and ≤17.5, >17.5–21.5, and >21.5 mmol/L for bicarbonate. Database-specific complete-case Model 3b adjustment sets were used, consistent with the primary association analyses. Upper chloride plus upper bicarbonate was the prespecified reference for this CART sensitivity analysis. In MIMIC-IV (n=13,168; events=2,370), the global 8-df likelihood-ratio test was χ²=18.36, P=0.0187; in eICU (n=21,414; events=3,907), χ²=86.11, P<0.001. All eight nonreference cell-specific adjusted ORs are reported; the MIMIC-derived CART cut points are prognostic sensitivity thresholds and should not be interpreted as therapeutic boundaries.

**Supplementary Table 18.** Harmonized common-covariate phenotype estimates and cross-database heterogeneity.

| **Phenotype** | **MIMIC-IV OR (95% CI)** | **eICU OR (95% CI)** | **Pooled OR (95% CI)** | **I² (%)** | **Cohort-difference P** | **FDR q** |
| --- | --- | --- | --- | --- | --- | --- |
| Low Cl + Low HCO₃ | 1.43 (1.13–1.80) | 1.58 (1.31–1.90) | 1.52 (1.31–1.75) | 0.0 | 0.500 | 0.666 |
| Low Cl + Normal HCO₃ | 2.01 (1.56–2.57) | 1.42 (1.16–1.73) | 1.62 (1.39–1.89) | 78.1 | 0.032 | 0.084 |
| Low Cl + High HCO₃ | 1.58 (1.21–2.07) | 1.13 (0.93–1.36) | 1.26 (1.08–1.47) | 75.8 | 0.042 | 0.084 |
| Normal Cl + Low HCO₃ | 1.25 (1.07–1.46) | 1.35 (1.19–1.53) | 1.31 (1.19–1.44) | 0.0 | 0.456 | 0.666 |
| Normal Cl + High HCO₃ | 1.06 (0.86–1.32) | 1.06 (0.91–1.23) | 1.06 (0.94–1.20) | 0.0 | 0.964 | 0.964 |
| High Cl + Low HCO₃ | 0.98 (0.83–1.14) | 1.32 (1.16–1.49) | 1.18 (1.07–1.30) | 88.3 | 0.003 | 0.014 |
| High Cl + Normal HCO₃ | 0.49 (0.41–0.60) | 0.85 (0.73–0.99) | 0.69 (0.61–0.78) | 94.6 | <0.001 | <0.001 |
| High Cl + High HCO₃ | 0.94 (0.64–1.38) | 1.00 (0.78–1.27) | 0.98 (0.80–1.20) | 0.0 | 0.792 | 0.905 |

*Note.* Normal chloride plus normal bicarbonate is the reference. Both databases used the same adjustment structure: age, sex, harmonized race, Charlson index, SOFA, creatinine, blood urea nitrogen, lactate, septic shock, AKI, invasive ventilation, sodium bicarbonate therapy, and vasoactive therapy. Continuous covariates were standardized within cohort. Pooled estimates are inverse-variance fixed effects and should be interpreted cautiously with only two cohorts. FDR q values use Benjamini-Hochberg adjustment across the eight phenotype contrasts. The original patient-level phenotype-by-cohort interaction using database-specific Model 3b adjustment blocks was a global 8-df Wald test (χ²=12.41, P=0.134). Under harmonized adjustment, the global 8-df cross-cohort coefficient comparison was χ²=46.65, P<0.001.

**Supplementary Table 19.** External-validation integrated discrimination improvement and category-free net reclassification improvement.

| **Metric** | **Estimate** | **95% CI** |
| --- | --- | --- |
| IDI | -0.0035 | -0.0053 to -0.0017 |
| Continuous NRI | -0.1149 | -0.1492 to -0.0826 |
| NRI event component | -0.1336 | -0.1627 to -0.1052 |
| NRI non-event component | 0.0187 | 0.0063 to 0.0320 |

*Note.* Metrics compare the MIMIC-IV-trained portable base model with the same model plus the clinical 3 × 3 phenotype when applied to eICU without refitting. Confidence intervals are from 500 paired case-resampling bootstrap replicates with fixed predictions. Positive IDI/NRI would indicate improvement; the observed negative values do not support improved discrimination/reclassification. Primary AUROC, Brier, and calibration results remain those reported in Table 5 and Supplementary Table 14.

**Supplementary Table 20A.** Dual-database 24-hour low-low phenotype transition analysis.

| **Cohort** | **Transition** | **n** | **Deaths** | **Mortality (%)** | **Adjusted OR (95% CI)** | **P** | **Global P** |
| --- | --- | --- | --- | --- | --- | --- | --- |
| MIMIC-IV | Never low-low | 13,173 | 2,018 | 15.3 | Reference | — | — |
| MIMIC-IV | Resolved low-low | 504 | 134 | 26.6 | 1.33 (1.08–1.65) | 0.008 | <0.001 |
| MIMIC-IV | Persistent low-low | 360 | 156 | 43.3 | 2.67 (2.13–3.34) | <0.001 | <0.001 |
| MIMIC-IV | Incident low-low | 226 | 106 | 46.9 | 3.45 (2.60–4.57) | <0.001 | <0.001 |
| eICU | Never low-low | 7,560 | 1,365 | 18.1 | Reference | — | — |
| eICU | Resolved low-low | 438 | 80 | 18.3 | 1.08 (0.84–1.39) | 0.551 | <0.001 |
| eICU | Persistent low-low | 181 | 55 | 30.4 | 2.19 (1.57–3.04) | <0.001 | <0.001 |
| eICU | Incident low-low | 116 | 53 | 45.7 | 4.10 (2.81–5.99) | <0.001 | <0.001 |

*Note.* Early status used the earliest exact-time chloride-bicarbonate pair at 0–6 hours; later status used the latest exact-time pair at 12–24 hours. Patients had to remain in the ICU through 24 hours. Low-low was chloride <98 mmol/L plus bicarbonate <22 mmol/L. Models adjusted only fixed/admission covariates to reduce post-baseline adjustment. The outcome was subsequent in-hospital mortality. These landmark analyses are exploratory and do not establish that resolution of the phenotype reduces mortality. Transition models adjusted only fixed/admission covariates and therefore did not comprehensively account for evolving early illness severity.

**Supplementary Table 20B.** Descriptive cross-cohort synthesis of 24-hour low-low transitions.

| **Contrast** | **MIMIC-IV OR (95% CI)** | **eICU OR (95% CI)** | **Pooled OR (95% CI)** | **I² (%)** | **Heterogeneity P** |
| --- | --- | --- | --- | --- | --- |
| Resolved low-low versus Never low-low | 1.33 (1.08–1.65) | 1.08 (0.84–1.39) | 1.22 (1.04–1.44) | 35.8 | 0.212 |
| Persistent low-low versus Never low-low | 2.67 (2.13–3.34) | 2.19 (1.57–3.04) | 2.50 (2.08–3.01) | 0.0 | 0.330 |
| Incident low-low versus Never low-low | 3.45 (2.60–4.57) | 4.10 (2.81–5.99) | 3.67 (2.93–4.60) | 0.0 | 0.473 |

*Note.* Pooled estimates use inverse-variance fixed effects and are provided as a descriptive cross-cohort summary. I² is unstable with only two cohorts. Transition models used database-specific fixed/admission covariates as described in the Supplementary analytical methods.

**Supplementary Table 21A.** Dual-database continuous chloride and bicarbonate change models in the 24-hour landmark cohorts.

| **Cohort** | **n** | **Term** | **Adjusted OR (95% CI)** | **P** | **Joint Δ LR χ²** | **Joint Δ P** |
| --- | --- | --- | --- | --- | --- | --- |
| MIMIC-IV | 14,263 | Early chloride, per 1 SD | 0.79 (0.75–0.83) | <0.001 | 258.94 | <0.001 |
| MIMIC-IV | 14,263 | Early bicarbonate, per 1 SD | 0.63 (0.60–0.66) | <0.001 | 258.94 | <0.001 |
| MIMIC-IV | 14,263 | Δ chloride (12–24 h minus 0–6 h), per 1 SD | 0.96 (0.90–1.01) | 0.103 | 258.94 | <0.001 |
| MIMIC-IV | 14,263 | Δ bicarbonate (12–24 h minus 0–6 h), per 1 SD | 0.66 (0.63–0.70) | <0.001 | 258.94 | <0.001 |
| eICU | 8,295 | Early chloride, per 1 SD | 0.85 (0.79–0.90) | <0.001 | 225.09 | <0.001 |
| eICU | 8,295 | Early bicarbonate, per 1 SD | 0.59 (0.55–0.64) | <0.001 | 225.09 | <0.001 |
| eICU | 8,295 | Δ chloride (12–24 h minus 0–6 h), per 1 SD | 0.83 (0.78–0.89) | <0.001 | 225.09 | <0.001 |
| eICU | 8,295 | Δ bicarbonate (12–24 h minus 0–6 h), per 1 SD | 0.59 (0.55–0.63) | <0.001 | 225.09 | <0.001 |

*Note.* Delta equals the later 12–24-hour value minus the early 0–6-hour value. Early chloride, early bicarbonate, delta chloride, and delta bicarbonate were entered simultaneously with the same fixed/admission covariates used in the transition analysis. The joint delta likelihood-ratio test compares the model containing both change terms with the model containing early values and covariates only. Scaling is cohort specific: SDs for delta chloride were 4.72 mmol/L in MIMIC-IV and 4.79 mmol/L in eICU; SDs for delta bicarbonate were 3.39 and 4.28 mmol/L, respectively.

**Supplementary Table 21B.** Descriptive cross-cohort comparison of continuous first-day chloride and bicarbonate changes.

| **Change term** | **MIMIC-IV OR (95% CI)** | **P** | **eICU OR (95% CI)** | **P** | **I² (%)** | **Heterogeneity P** |
| --- | --- | --- | --- | --- | --- | --- |
| Δ chloride (12–24 h minus 0–6 h), per 1 SD | 0.96 (0.90–1.01) | 0.103 | 0.83 (0.78–0.89) | <0.001 | 90.1 | 0.001 |
| Δ bicarbonate (12–24 h minus 0–6 h), per 1 SD | 0.66 (0.63–0.70) | <0.001 | 0.59 (0.55–0.63) | <0.001 | 84.4 | 0.011 |

*Note.* One-SD scaling is cohort specific, so these cross-cohort comparisons and any pooled estimates are descriptive. The bicarbonate-change association was directionally consistent but differed in magnitude; the chloride-change association was weaker and not statistically significant in MIMIC-IV but significant in eICU. Because the effect scale differs by cohort, I² and heterogeneity P values should be interpreted only as descriptive diagnostics rather than formal like-for-like heterogeneity tests.

**Supplementary Table 21C.** Timing-adjusted sensitivity analysis of continuous first-day chloride and bicarbonate changes.

| **Cohort** | **n** | **Events** | **Term** | **Adjusted OR (95% CI)** | **P** |
| --- | --- | --- | --- | --- | --- |
| MIMIC-IV | 14,263 | 2,414 | Δ chloride (12–24 h minus 0–6 h), per 1 SD | 0.949 (0.899–1.002) | 0.060 |
| MIMIC-IV | 14,263 | 2,414 | Δ bicarbonate (12–24 h minus 0–6 h), per 1 SD | 0.659 (0.626–0.694) | <0.001 |
| eICU | 8,295 | 1,553 | Δ chloride (12–24 h minus 0–6 h), per 1 SD | 0.826 (0.773–0.882) | <0.001 |
| eICU | 8,295 | 1,553 | Δ bicarbonate (12–24 h minus 0–6 h), per 1 SD | 0.588 (0.547–0.632) | <0.001 |

*Note.* Timing-adjusted sensitivity models additionally accounted for early measurement time and the interval between the early and later measurements. These analyses remain exploratory and do not support causal interpretation.

**Supplementary Figures**

**Supplementary Figure 1.** Distribution of CHBI in MIMIC-IV v3.1 and eICU v2.0. Binned aggregate counts are shown for CHBI in both cohorts. The figure is based on aggregate binned outputs and does not contain patient-level data.

**Supplementary Figure 2.** Crude in-hospital mortality across CHBI quartiles. Bars show crude in-hospital mortality percentages by CHBI quartile in MIMIC-IV v3.1 and eICU v2.0. Values above bars are mortality percentages. These crude, unadjusted percentages may differ from the multivariable-adjusted estimates shown in the forest plots.

**Supplementary Figure 3.** Complete-case Model 3b forest plot for CHBI exposure specifications. Odds ratios and 95% confidence intervals are shown for CHBI per 1-SD increment, absolute CHBI per 1-SD increment, CHBI quartiles, and chloride-bicarbonate joint phenotypes. Reference categories are Q2 for CHBI quartiles and normal chloride plus normal bicarbonate for joint phenotypes.

**Supplementary Figure 4.** Predictive mean matching multiple-imputation forest plot. Model 3b odds ratios and 95% confidence intervals were pooled across 20 donor-based predictive mean matching imputed datasets using Rubin’s rules. The reference group was normal chloride plus normal bicarbonate.

**Supplementary Figure 5.** Exposure-definition sensitivity analysis forest plot. The primary high-risk contrast, low chloride plus low bicarbonate versus normal chloride plus normal bicarbonate, is shown across laboratory pairing and early exposure-window sensitivity analyses. Global phenotype P values correspond to the global phenotype test.

**Supplementary Figure 6.** Subgroup analysis forest plot. The primary high-risk contrast, low chloride plus low bicarbonate versus normal chloride plus normal bicarbonate, is shown across prespecified subgroups. Interaction P values are 8-df Wald tests from joint phenotype-by-subgroup models and are displayed once per subgroup. NE indicates not estimable. The MIMIC-IV sodium bicarbonate therapy interaction was not reliably estimable because a sparse treated high-chloride/high-bicarbonate cell contained no deaths.

**Supplementary Figure 7.** Secondary outcome forest plot. The primary high-risk contrast, low chloride plus low bicarbonate versus normal chloride plus normal bicarbonate, is shown for MIMIC-IV in-hospital, 28-day, and 90-day mortality and for eICU in-hospital mortality and in-hospital death by days 28 and 90. Because eICU did not capture post-discharge deaths, the latter endpoints are not conventional all-cause 28-day or 90-day mortality. Global phenotype P values correspond to the global phenotype test.

**Supplementary Figure 8.** Simultaneous spline-adjusted chloride association in MIMIC-IV v3.1. Chloride and bicarbonate were modeled simultaneously using natural cubic splines with 4 degrees of freedom per exposure and complete-case Model 3b covariates. Bicarbonate was fixed at 22 mmol/L, and chloride 106 mmol/L was the reference value. The shaded region indicates the 95% confidence interval.

**Supplementary Figure 9.** Simultaneous spline-adjusted bicarbonate association in MIMIC-IV v3.1. Chloride and bicarbonate were modeled simultaneously using natural cubic splines with 4 degrees of freedom per exposure and complete-case Model 3b covariates. Chloride was fixed at 106 mmol/L, and bicarbonate 22 mmol/L was the reference value. The shaded region indicates the 95% confidence interval.

**Supplementary Figure 10.** Simultaneous spline-adjusted chloride association in eICU v2.0. Chloride and bicarbonate were modeled simultaneously using natural cubic splines with 4 degrees of freedom per exposure and complete-case Model 3b covariates. Bicarbonate was fixed at 22 mmol/L, and chloride 105 mmol/L was the reference value. The shaded region indicates the 95% confidence interval.

**Supplementary Figure 11.** Simultaneous spline-adjusted bicarbonate association in eICU v2.0. Chloride and bicarbonate were modeled simultaneously using natural cubic splines with 4 degrees of freedom per exposure and complete-case Model 3b covariates. Chloride was fixed at 105 mmol/L, and bicarbonate 22 mmol/L was the reference value. The shaded region indicates the 95% confidence interval.
